# Preventing Acute Malnutrition among Young Children in Crises: A Prospective Intervention Study in Niger

**DOI:** 10.1371/journal.pmed.1001714

**Published:** 2014-09-02

**Authors:** Céline Langendorf, Thomas Roederer, Saskia de Pee, Denise Brown, Stéphane Doyon, Abdoul-Aziz Mamaty, Lynda W.-M. Touré, Mahamane L. Manzo, Rebecca F. Grais

**Affiliations:** 1 Epicentre, Paris, France; 2 Policy and Strategy Division, World Food Programme, Rome, Italy; 3 World Food Programme, Niamey, Niger; 4 Médecins Sans Frontières, Paris, France; 5 Epicentre, Niger; 6 Regional Department of the Ministry of Public Health, Maradi, Niger; Centre for Global Child Health, Hospital for Sick Children, Toronto, Canada and Center of Excellence in Women and Child Health, The Aga Khan University, Karachi, Pakistan

## Abstract

**Background:**

Finding the most appropriate strategy for the prevention of moderate acute malnutrition (MAM) and severe acute malnutrition (SAM) in young children is essential in countries like Niger with annual “hunger gaps.” Options for large-scale prevention include distribution of supplementary foods, such as fortified-blended foods or lipid-based nutrient supplements (LNSs) with or without household support (cash or food transfer). To date, there has been no direct controlled comparison between these strategies leading to debate concerning their effectiveness. We compared the effectiveness of seven preventive strategies—including distribution of nutritious supplementary foods, with or without additional household support (family food ration or cash transfer), and cash transfer only—on the incidence of SAM and MAM among children aged 6–23 months over a 5-month period, partly overlapping the hunger gap, in Maradi region, Niger. We hypothesized that distributions of supplementary foods would more effectively reduce the incidence of acute malnutrition than distributions of household support by cash transfer.

**Methods and Findings:**

We conducted a prospective intervention study in 48 rural villages located within 15 km of a health center supported by Forum Santé Niger (FORSANI)/Médecins Sans Frontières in Madarounfa. Seven groups of villages (five to 11 villages) were allocated to different strategies of monthly distributions targeting households including at least one child measuring 60 cm–80 cm (at any time during the study period whatever their nutritional status): three groups received high-quantity LNS (HQ-LNS) or medium-quantity LNS (MQ-LNS) or Super Cereal Plus (SC+) with cash (€38/month [US$52/month]); one group received SC+ and family food ration; two groups received HQ-LNS or SC+ only; one group received cash only (€43/month [US$59/month]). Children 60 cm–80 cm of participating households were assessed at each monthly distribution from August to December 2011. Primary endpoints were SAM (weight-for-length Z-score [WLZ]<−3 and/or mid-upper arm circumference [MUAC]<11.5 cm and/or bipedal edema) and MAM (−3≤WLZ<−2 and/or 11.5≤MUAC<12.5 cm). A total of 5,395 children were included in the analysis (615 to 1,054 per group). Incidence of MAM was twice lower in the strategies receiving a food supplement combined with cash compared with the cash-only strategy (cash versus HQ-LNS/cash adjusted hazard ratio [HR] = 2.30, 95% CI 1.60–3.29; cash versus SC+/cash HR = 2.42, 95% CI 1.39–4.21; cash versus MQ-LNS/cash HR = 2.07, 95% CI 1.52–2.83) or with the supplementary food only groups (HQ-LNS versus HQ-LNS/cash HR = 1.84, 95% CI 1.35–2.51; SC+ versus SC+/cash HR = 2.53, 95% CI 1.47–4.35). In addition, the incidence of SAM was three times lower in the SC+/cash group compared with the SC+ only group (SC+ only versus SC+/cash HR = 3.13, 95% CI 1.65–5.94). However, non-quantified differences between groups, may limit the interpretation of the impact of the strategies.

**Conclusions:**

Preventive distributions combining a supplementary food and cash transfer had a better preventive effect on MAM and SAM than strategies relying on cash transfer or supplementary food alone. As a result, distribution of nutritious supplementary foods to young children in conjunction with household support should remain a pillar of emergency nutritional interventions. Additional rigorous research is vital to evaluate the effectiveness of these and other nutritional interventions in diverse settings.

**Trial registration:**

ClinicalTrials.gov NCT01828814

*Please see later in the article for the Editors' Summary*

## Abstract 2

Céline Langendorf and colleagues conducted a pragmatic intervention study in Niger to assess whether distributions of supplementary foods in addition to household support by cash transfer effectively reduced malnutrition in children aged 6 to 23 months.

*Please see later in the article for the Editors' Summary*

## Editors' Summary

**Background:**

Good nutrition during childhood is essential for health and survival. Undernourished children are more susceptible to infection and are more likely to die from common ailments such as diarrhoea than well-nourished children. Malnutrition can be chronic or acute. Chronic (long-term) malnutrition causes stunting. A stunted child has a low height for his or her age when compared to the World Health Organization Child Growth Standards, which chart the growth of a reference population. By contrast, acute malnutrition causes wasting. A wasted child has a low weight for his or her height. Acute malnutrition, which is responsible for more than 800,000 deaths among children under 5 years old every year (12.6% of all deaths in this age group), often follows earthquakes or other emergencies but also occurs in countries such as Niger that regularly experience a “hunger gap,” a shortage of food in the months before the annual harvest. Interventions designed to prevent acute malnutrition include distributions of supplementary food such as lipid-based nutrient supplements (LNS) and fortified cereal products (for example, super cereal plus [SC+]) targeting children and household support in the form of food or cash transfers, which allow households to buy their own food.

**Why Was This Study Done?:**

Governments and donor agencies need to know which preventative strategy is most effective, particularly among children under 2 years old who are most vulnerable to acute malnutrition. Here, the researchers compare the effectiveness of seven preventative strategies—including the distribution of nutritious supplementary foods with and without additional household support (food or cash transfer) and cash transfer alone—on the incidence (occurrence) of moderate and severe acute malnutrition among children aged 6–23 months living in 48 villages in the Madarounfa district of Niger between August and December, 2011. Niger regularly experiences a hunger gap between June and October; 12.9% of the population of the Madarounfa district was at risk of severe food insecurity at the time of the study.

**What Did the Researchers Do and Find?:**

The researchers allocated the villages to receive high or medium quantity LNS plus cash, SC+ plus cash or a family food ration, high quantity LNS only, SC+ only, or cash only; the cash transfer was sufficient to meet the energy needs of an average household. The interventions were distributed once a month to households that included at least one child measuring 60–80 cm in length at any time during the study period (length is used as a proxy for age when the precise age of children is unknown). Eligible children in each participating household were assessed for moderate and severe acute malnutrition as defined by the World Health Organization by measuring their weight, length, and mid-upper arm circumference at each distribution. The incidence of moderate acute malnutrition was about twice as high among children who received the cash-only intervention as among those who received any of the food supplement plus cash. For example, the incidence of moderate acute malnutrition was 3.33 cases per 100 child-months among children who received the SC+ plus cash intervention but 7.97 cases per 100 child-months among those who received the cash-only intervention. Moreover, the incidence of severe acute malnutrition was about three times higher among the children who received the SC+ only intervention than among those who received the SC+ plus cash intervention.

**What Do These Findings Mean?:**

These findings suggest that, in this region of Niger, interventions that combined the distribution of supplementary food and a cash transfer prevented acute malnutrition more effectively than strategies that relied on cash transfer or supplementary food distribution alone. Unmeasured differences between the groups that affect the incidence of malnutrition may be responsible for some of the differences between intervention groups, and the incidences of malnutrition may have been different if the study period had covered the whole hunger gap. Moreover, these findings may not be generalizable to other regions of Niger or to other countries that experience an annual hunger gap. Despite these limitations, which highlight the need for further research into the effectiveness of nutritional interventions, these findings suggest that the distribution of supplementary foods to young children in conjunction with household support should remain an important part of nutritional interventions during the hunger gap and other emergencies.

**Additional Information:**

Please access these websites via the online version of this summary at http://dx.doi.org/10.1371/journal.pmed.1001714.

- This study is further discussed in a *PLOS Medicine* Perspective by Marco Kerac and Andrew Seal
- More information about this prospective intervention study is available from ClinicalTrials.gov
- The charity UNICEF, which protects the rights of children and young people around the world, provides detailed statistics on child undernutrition; it has detailed information about malnutrition and the humanitarian situation in Niger; a 2012 article on malnutrition among children in Niger includes a personal story about a child with severe acute malnutrition in Maradi
- The World Health Organization Child Growth Standards are available (in several languages)
- The United Nations Millennium Development Goals provides information on ongoing world efforts to reduce hunger and child mortality
- The World Food Programme is the world's largest humanitarian agency fighting hunger worldwide; its website provides information about malnutrition in Niger, including a visual presentation on wide-scale targeted food distribution in the country
- “Starved for Attention” is an international multimedia campaign launched in 2010 by Médecins Sans Frontiéres (MSF) and the VII Photo agency to rewrite the story of childhood malnutrition; information about MSF's work in Niger to tackle malnutrition is available

## Introduction

Undernutrition covers a range of disorders including impaired growth and micronutrient deficiencies. Of these disorders, acute malnutrition, or wasting, is an attributable cause of 12.6% of the 6.9 million deaths among children under 5 years old, accounting for more than 800,000 deaths annually [1]. Marasmus is the most common form of acute malnutrition in nutritional crises and, in its severe form, can lead to death if untreated [2]. A wasted child may be classified as either moderately or severely acutely malnourished on the basis of body measurements. Mid-upper arm circumference (MUAC) and weight-for-height Z-score (WHZ) are the indicators used to classify a child with wasting. Moderate acute malnutrition (MAM) is defined as a WHZ<−2 WHZ but ≥−3 WHZ using the WHO Child Growth Standards. Severe acute malnutrition (SAM) is defined as MUAC<11.5 cm and/or WHZ<−3 and/or bilateral pitting edema. Global acute malnutrition (GAM) is the sum of MAM and SAM at the population level [3].

One way to reduce the burden of acute malnutrition is to prevent its emergence among children under age two, the most vulnerable group [4]. Although considerable progress has been made in treating SAM [2],[5],[6], prevention has received far less research attention and there is little evidence that can serve as solid argumentation for malnutrition prevention policies and programs [7]. Prevention of acute malnutrition aims both to prevent non-malnourished children from becoming MAM and for children classified with MAM to worsen and become SAM. Limited data suggest that community-wide preventive approaches have a greater impact on reducing childhood acute malnutrition than programs targeting already acutely malnourished children [8],[9]. However, much less is known about which prevention strategies work best in a given context, especially when targeting large populations with limited resources and integrated approaches that combine multiple interventions with different aims.

Preventive strategies are of the utmost importance during chronic or acute nutritional crises, defined as slow-onset crises such as when drought, crop failure or economic crises may erode livelihoods and undermine food supply thereby leading to vulnerabilities of households to meet their food needs [10]. In practice, nutritional programming during chronic or acute food crises may include large-scale distributions (“blanket distributions”) that specifically target the most vulnerable children with nutritious supplementary foods (e.g., fortified blended flours or ready-to-use foods) formulated for their specific nutritional requirements and/or provision of overall household support in the form of food or cash [10]–[12]. Distributions based on nutritious supplementary foods have been evaluated in several countries, using a variety of products that differ in terms of energy value, composition, and packaging. Among these, lipid-based nutrient supplements (LNSs) have been shown to reduce the incidence and prevalence of SAM [9],[13],[14] and to improve growth rates in children who were not malnourished or were moderately malnourished [15]–[19]. Super Cereal Plus (SC+), one of a new generation of fortified blended flours incorporating powdered milk, sugar, oil, and a modified mineral and vitamin premix, became available in early 2011 [20]. A clinical trial in Malawi showed non-inferiority of SC+ for treating MAM in children aged 6–59 months, compared with two LNSs [21]. Because of the large sample size that would be required, only very few studies of nutritious supplementary foods have assessed the effects of preventive distribution strategies on mortality [17],[19],[22].

Another preventive strategy, cash transfer, aims to strengthen food security for vulnerable households by giving families enough purchasing power to consume an adequate and balanced diet, maintain a good standard of hygiene, access health services, and invest in their own means of food production in addition to their children's growth and development. Programs of some international agencies and organizations rely exclusively on cash transfer and do not distribute food supplements, and some donors express a preference for cash transfers, not only to support livelihoods, but also to improve nutrition. While conditional cash transfer to vulnerable households has shown a long-term positive impact on growth and on malnutrition-related mortality in children aged 0–5 years, especially in Latin America [23]–[29], there is little conclusive evidence that cash transfer has a direct effect on preventing SAM and MAM [23],[30].

Filling the evidence gap regarding the best preventive strategies for a given context requires studies that directly compare the effectiveness of these different strategies alone and in combination. However, to date, to our knowledge, there have been no direct comparisons of different preventive strategies, including cash transfer in a single population and context, using the same methodology. Such knowledge is especially needed in high-burden countries of the Sahel (Niger, Chad, Mali), which regularly experience a “hunger gap” in the months before the annual harvest to guide humanitarian responses during nutritional or food crises. The extension of the period between the depletion of stored food and the new harvest, or the “hunger gap,” usually necessitates additional assistance. The timing and duration of the hunger gap varies from year to year depending on the timing and duration of the annual rains. During this time, young children are most vulnerable to insufficient food quantity and quality leading to increased prevalence of acute malnutrition.

Here, we hypothesized that distributions of supplementary foods, specifically adapted to meet the nutritional needs of children 6 to 23 months, would more effectively reduce the incidence of acute malnutrition among this group than distributions of household support by cash transfer. We present findings from a study comparing the effectiveness of seven preventive strategies—including distribution of nutritious supplementary foods (LNS or SC+), with or without additional household support (family food ration or cash transfer) and cash transfer only, on the incidence of SAM and MAM among children 6–23 months old over a 5-month period (partly overlapping the hunger gap) in Maradi region, Niger. The study was designed as a prospective intervention study to examine the effectiveness of interventions in normal practice. Our aim was to maximize applicability of results to usual care settings, and rely on unarguably important outcomes such as mortality and severe morbidity [31].

## Materials and Methods

### Ethical Considerations

The study protocol was approved by the National Ethical Committee of Niger's Ministry of Public Health and by the Comité de Protection des Personnes, Ile-de-France XI, France. Approval from the heads of all selected villages was received prior to starting the study. Study objectives and procedures were explained to heads of households or child caregivers before inclusion. An informed consent statement was read aloud in the local dialect before consenting adults signed or were fingerprinted. Participation in the study was not a pre-condition for obtaining free medical services and preventive distributions. It was clearly stated that participants were free to withdraw from the study at any time. All nutritional treatment was also provided free of charge irrespective of participation in the study. All children meeting admission criteria to a nutritional program were referred for care. This study was registered retrospectively on *clinicaltrials.gov* as NCT01828814. The delay was because of a lack of consensus with respect to whether this study required registration. We chose to register this study following WHO guidance on trials that state that “any research study that prospectively assigns human participants or groups of humans to one or more health-related interventions to evaluate the effects on health outcomes” was largely sufficient to justify registration. The protocol in French is available as Protocol S1.

### Study Setting

The “hunger gap” in Niger generally occurs from June to October, coinciding with the rainy season, when the incidence of both diarrheal disease and malaria is typically elevated. Each year since 2010, more than 300,000 children under age five have been admitted to therapeutic feeding programs (TFPs) for SAM, the majority of them during the hunger gap [32],[33]. Within this age group, a June 2011 national nutrition survey found, that the prevalence of SAM was 5-fold higher among children aged 6–23 months than among 24–59 month-olds (4.2% versus 0.8%, respectively) [34].

Our study was conducted in Madarounfa health district, in the southern part of Maradi region (Figure S1). In Maradi, the prevalence of SAM, GAM, and stunting (length-for-age Z-score (LAZ)<−2) in the target population (6–23 months) was estimated in June 2011 as 3.9%, 21.4%, and 59.5%, respectively [34]. The mostly rural Madarounfa health district has approximately 405,000 inhabitants, with the majority of households living on about 500 francs de la Communauté Financière Africaine (FCFA) per day (€0.75 [US$1]) [35]. On the basis of an Early Warning System survey conducted in January 2011, the district was declared to be at severe risk of food insecurity (12.9% of the population at risk of severe food insecurity in Madarounfa district versus 6.6% in rural areas nationally) [36]. Since 2008, five health areas in the district have been receiving medical and nutritional support from a joint project between Forum Santé Niger (FORSANI, a local NGO) and Médecins Sans Frontières (MSF), which runs five health centers with TFP and one intensive care hospital.

### Study Design

This study was designed as a prospective intervention study comparing the nutritional status of children 6–23 months assigned to one of seven different intervention groups, each receiving either one of several nutritious supplementary foods (high-quantity LNS [HQ-LNS] or medium-quantity LNS [MQ-LNS] or SC+) with or without additional household support (family food ration or cash transfer), or cash transfer only. These seven interventions are described in Table 1.

**Table 1:** Composition of monthly distributions in the seven intervention groups, August–December 2011.

Intervention Group	Nutritious Supplementary Foods for Children 6–23 Months	Household Support		Total Monthly Cost of All Transfers (Excluding Operational Costs) in Euros (US$)
		Cash Transfer	Family Food Ration	
HQ-LNS/cash	Supplementary Plumpy, 500 kcal/day, 92 g/day	25,000 FCFA (€38 = US$52)	—	45.27 (62.03)
MQ-LNS/cash	Plumpy'Doz, 250 kcal/day, 46 g/day	25,000 FCFA (€38 = US$52)	—	41.52 (56.86)
SC+/cash	Super Cereal Plus, 820 kcal/day, 200 g/day	25,000 FCFA (€38 = US$52)	—	43.70 (59.86)
SC+/food ration	Super Cereal Plus, 820 kcal/day, 200 g/day	—	50 kg cereals, 7.5 kg pulses, 2.5 kg oil	37.70 (52.17)
HQ-LNS	Supplementary Plumpy 500 kcal/day, 92 g/day	—	—	7.27 (10.03)
SC+	Super Cereal Plus, 820 kcal/day, 200 g/day	—	—	5.70 (7.86)
Cash only	—	28,000 FCFA (€43 = US$59)	—	3.52 (4.86)

The primary study outcome was the first event of SAM (weight-for-length Z-score [WLZ]<−3 and/or MUAC<11.5 cm and/or bipedal edema) or MAM (−3≤WLZ<−2 and/or 11.5≤MUAC<12.5 cm) according to the 2006 World Health Organization Child Growth Standards [6],[37].

*A priori* pairwise comparisons of primary interest were: cash only versus any other strategy; nutritious supplementary food only versus the same nutritious supplementary food combined with household support (cash or food ration); strategies combining cash and nutritious supplementary food versus each other; supplementary food only (SC+ versus HQ-LNS); strategies combining SC+ and both household supports. Over 5 months, we aimed to enrol between 500–800 eligible children in each group. A minimal sample size of 500 children per study group was required to detect a 20% difference in average WLZ (after a 5-month intervention) between the supplementary food (HQ-LNS) and cash transfer versus cash-only group, with 80% power at the 2-sided 5% level and a standard deviation of 1 for the WLZ within each group, a level 1 autoregressive-type covariance structure, a correlation of 0.5 between each measurement, and 10% loss to follow-up [14]. This sample size would provide 80% power to detect a difference of 30% in the incidence of SAM of at least 2 per 100 child-months between two groups, considering a 2-sided 95% confidence level [38].

Secondary outcomes included mortality and reported consummation of distributed food supplements during the 5-month follow-up. The proportion of monthly rations consumed within households or shared outside households and report of main consumers within households (whether the targeted child or someone else within the nuclear family or household) was recorded. In addition, linear growth and rate of MAM and SAM relapse and recovery over 18 months of follow-up continues in order to examine the potential long-term effectiveness of continual distributions in this population.

### Study Population

**Figure 1:**
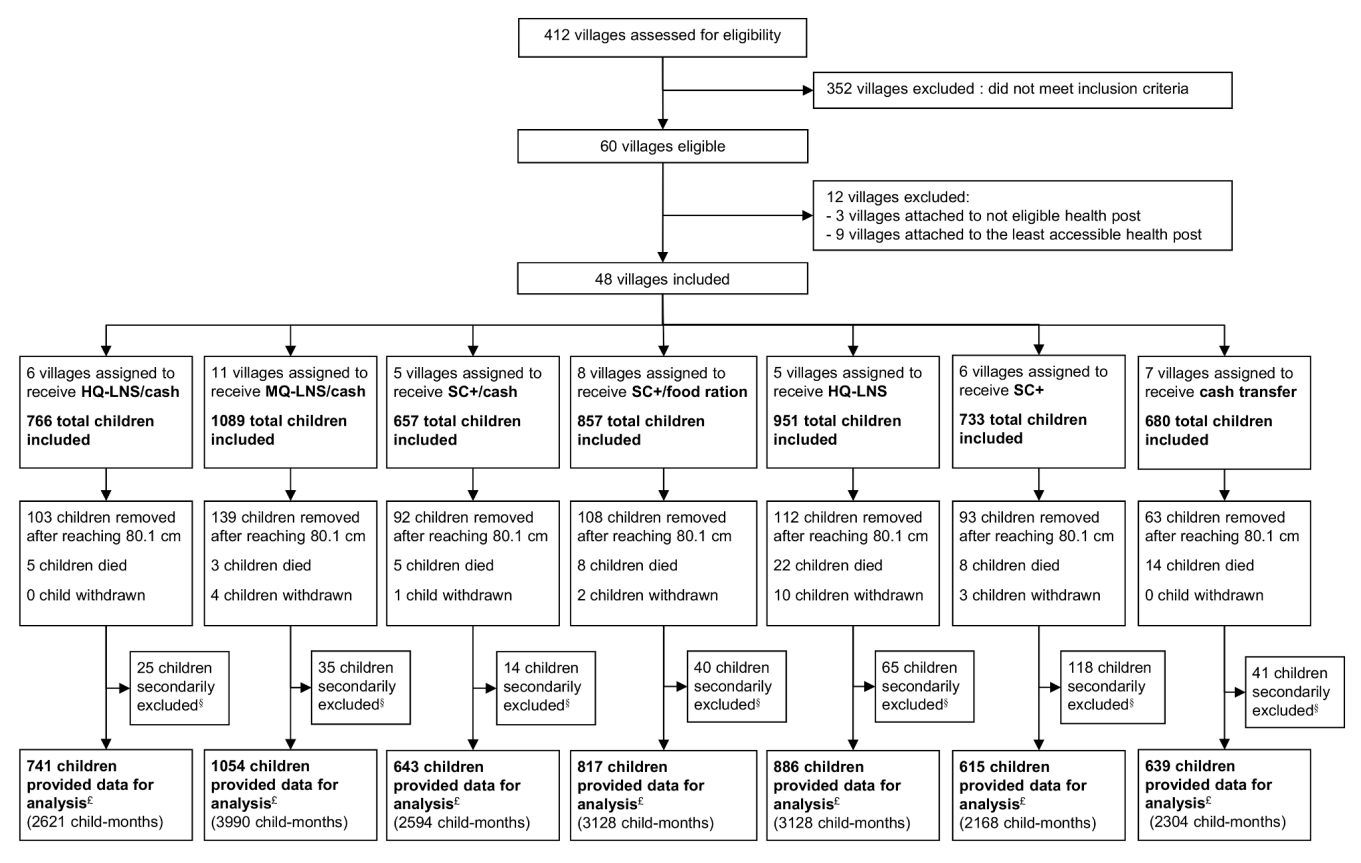
Intervention assignment and study flow of participants. ^§^Did not meet the inclusion criteria (were not living in a study village or were not the child initially enrolled). ^£^All included children minus secondarily excluded children.

Madarounfa health district encompasses a total of 412 villages and hamlets, based on the official list provided by local health authorities. We considered only villages located in a rural area and within 15 km of a FORSANI/MSF-supported health center that provided care free of charge. In addition to ensuring access to the same comprehensive primary care and nutrition program, selection of this health district also allowed us to ensure that no other nutritional interventions or general food distributions would be implemented in the study area during the study period. Of all villages, 60 met these criteria. From these 60 villages, we created seven groups of geographically close villages, each group with 500–800 children aged 6–23 months according to the most recent Niger census. Twelve of these villages were not included as they were not needed to meet the sample size and presented access concerns (Figure 1). A total of 48 villages and hamlets were included.

After consent from village leaders, all households of the selected villages were visited at home prior to any distribution for a baseline enrolment visit. The representative of each eligible child (mother or primary caregiver) was invited to provide consent for participation. Child inclusion criteria were: residing in the village and measuring >60.0 cm and ≤80.0 cm in length irrespective of their nutritional status. Children meeting admission criteria to SAM treatment programs at the initial baseline visit were referred for care.

Since precise age was usually unknown, length was taken as a proxy of age, as per common practice in large-scale nutritional interventions [9],[22],[39]. Because of the high prevalence of stunting, we selected 60.0 cm and 80.0 cm as the proxy length for children 6 months and 23 months old, respectively. From August to November 2011, monthly visits were conducted in each village to include children reaching a length of 60.1 cm. They were invited to participate to distributions from the month following their inclusion. The only exclusion criterion was known severe food allergy. Each representative received a follow-up booklet and a beneficiary card.

All villages within each group were assigned to the same nutritional intervention, to reduce the likelihood of contamination between groups through the sharing of supplemental foods or cash, including among households of neighboring villages.

Group assignment was random for four intervention groups using a computer generated random sequence (MS Excel, Microsoft Corporation). We forced the assignment of the MQ-LNS+ cash transfer to the group of villages within the same health area as villages eligible to receive the same nutritious supplementary food through a planned distribution program so as not interfere with existing programming. Two geographically proximate groups were forced to receive short-term distributions. Neither children and their families nor the field study team were blinded to the different interventions, even for the three nutritious supplementary foods (since they differ in appearance, taste, and preparation required).

### Interventions

Seven intervention groups (Table 1) were implemented from August to December 2011. Six groups each received a nutritious supplementary food, either HQ-LNS 500 kcal/day (Supplementary Plumpy, Nutriset), MQ-LNS 250 kcal/day (Plumpy'Doz, Nutriset), or Super Cereal Plus 820 kcal/day (Michiels and Cerfar). The amount of the daily rations followed standard practices in large-scale nutrition programs that aim to prevent an increase of wasting for example in lean seasons or during an emergency (92 g/day of HQ-LNS; 46 g/day of MQ-LNS; 200 g/day of SC+), to facilitate comparison of results to those from interventions as they are implemented in practice. The composition of each of these rations is described in Table 2.

**Table 2:** Nutrient composition of the nutritious supplementary foods used in this study per daily ration.

Nutrient Composition	MQ-LNS = Plumpy'Doz	HQ-LNS = Supplementary Plumpy	SC+ = Super Cereal Plus
	Daily Ration = 46 g	Daily Ration = 92 g	Daily Ration = 200 g
Energy (Kcal)	247	500	820
Protein (g)	5.9	12.5	24.8
Fat (g)	16	32.9	7.6
Calcium (mg)	387	276	260
Phosphorus (mg)	275	276	400
Potassium (mg)	310	511	800
Magnesium (mg)	60	84.6	—
Zinc (mg)	9.0	12.9	10
Copper (mg)	0.3	1.6	—
Iron (mg)	9	10.6	8
Iodine (µg)	90	92	80
Selenium (µg)	17	27.6	—
Vitamin A (µg)	400	840	3,328
Folic acid (µg)	160	193	120
Thiamine (mg)	0.5	0.55	0.3
Riboflavin (mg)	0.5	1.66	0.9
Niacin (mg)	6	4.88	9.6
Pantothenic acid (mg)	2.0	2.85	13.4
Vitamin B6 (mg)	0.5	0.55	3.4
Biotin (µg)	—	60	—
Vitamin B12 (µg)	0.9	1.7	4
Vitamin C (mg)	30	49	200
Vitamin D (µg)	—	15	8
Vitamin E (mg)	6	18.4	16.6
Vitamin K (µg)	—	19.3	200

Three groups also received household support in the form of a cash transfer. The cash transfer value of 25,000 FCFA (€38 [US$52]) per month was recommended by the Government of Niger and is similar to the amounts distributed by other international organizations in the region [30],[40]. It was calculated to be sufficient for vulnerable households (seven persons) to buy basic food commodities that meet their energy requirements, based on a recommended intake of 2,100 kcal/person/day. In the group that received only a monthly cash transfer (28,000 FCFA = €43 = US$59), the additional 3,000 FCFA (€5 [US$7]) were intended to cover the nutritional requirements of the under-two-year-old child who was not receiving a nutritious supplementary food. One group received household support in the form of food, comprising cereals, pulses, and oil.

The food/cash distribution procedure was based on those used by national and international organizations for large-scale nutrition programs in Niger.

Because previous studies in the same context have shown the effectiveness of large-scale distribution interventions of supplementary food, we decided not to have a formal control group (without any additional nutritional or cash support) [14],[22]. In addition, as our target population included children with MAM, international and national guidelines recommend addressing MAM (either in prevention of SAM or in specific treatment programs) as a component of community-based management of acute malnutrition [41],[42].

The first distribution took place on the 2 August 2011 and thereafter every 30 days up to the 13 of December 2011, resulting in five monthly distributions. Each distribution site was visited by teams in charge of giving out the monthly ration of nutritious supplementary foods and household support for each intervention group. All distribution sites were located within 3 km of their respective villages. Food supplements and direct cash transfers were given only to mothers or caregivers accompanied by the enrolled child and who presented the child's follow-up booklet and beneficiary card, both issued at enrolment. Interventions were not distributed at any other point in time. Prior to receiving each distribution, groups of ten caregivers at a time, took part in an education session. These sessions were led by a dedicated educational assistant and one to two women from the community (e.g., a village matriarch, women's representative, or midwife). With the help of visual aids, they gave caregivers information on topics such as breastfeeding, feeding children appropriately for their age group (e.g., enriched porridge and semi-solid snacks from 6 months of age then solid enriched foods from 12 months of age), food health and safety, prevention of malaria, vaccinations, and access to primary care based on Essential Nutrition Actions [43]. They also provided training on correct use of the nutritious supplementary foods distributed to each intervention group. Groups receiving cash transfer were given nutritional information on purchasing food for the targeted child, which included education on diet diversity (e.g., addition of enriched food such as oil, animal food, egg, beans, fruits, and vegetables to breast milk and local porridge).

Included children were followed up monthly at each distribution during which anthropometric measurements were recorded for each child. Included children who reached 80.1 cm were dropped from further follow-up after receiving a final distribution. Children classified as SAM were transferred to the nearest TFP, accompanied by a study nurse. At subsequent visits to distribution sites, children enrolled in a TFP (and therefore receiving ready-to-use therapeutic food as per national guidelines) received the household support but not the food supplement. Once these children were discharged from the treatment program, they again began receiving the assigned food supplement at the next distribution. Children who failed to appear at any distribution site were traced at home in the subsequent 10 days.

### Data Collection

At the initial recruitment visit at the child's home as well as subsequently at the site of distribution (follow-up visits were conducted immediately prior to distribution), field teams of trained nutrition assistants and nurses conducted anthropometric measurements (length, weight, MUAC) using standardized methods and calibrated instruments, as previously described [22]. If an exact birth date was unknown, we estimated child age using a calendar listing special events in the community. Nutritional edema was assessed by applying gentle thumb pressure on the dorsum of the feet and assessing for residual depression. Two different nutrition assistants took each child's anthropometric measurements, and the average of the two measures was recorded. If the two values deviated (±0.5 cm, ±0.2 kg), a third nutrition assistant took measurements and the average of the two closest values was used. An independent team conducted quality control assessments of anthropometric data every 2 months on a randomly selected sample of 10% of the children.

At each distribution visit, caregivers responded to a standardized questionnaire administered by a study team member about consumption or other use during the previous month of nutritious supplementary foods received. We solicited information about the quantity of the food consumed within the household, stored, exchanged or given away outside the household. We also asked each caregiver which household members mainly consumed the nutritious supplementary food over the previous month (multiple responses allowed). A household was defined as individuals who pool all or some of their resources, eat their main meal together, acknowledge the authority of the same person as head of household, and live together in/cohabit the same dwelling. A nuclear family was defined as a caregiver and the children under his/her charge. A household may include several nuclear families.

When a child failed to appear for the distribution, a study team member went to the house within the subsequent 10 days. S/he questioned family members about the reasons for their absence and, if the child was at home, took anthropometric measurements. In the case of death, a medical staff member subsequently visited the household to conduct a verbal autopsy (adapted from WHO guidelines [44]), to determine the main cause of death.

### Data Analysis

Analyses were by “intention-to-treat” considering all villages, households, and children as having received the allocated strategy. We calculated the incidence of SAM among children free of SAM at baseline, and the incidence of MAM among children free of SAM and MAM at baseline. Incidence and mortality rates were estimated per 100 child-months (person-time). Mortality events included all reports for which the cause for absence from surveillance visits was reported to be death by a family member or the head of village. We examined the distribution of baseline characteristics within each intervention group using generalized estimating equations with robust standard errors.

Next, we explored the association between supplementation strategy and the incidence of SAM, MAM, and mortality. Among children free from the outcome at baseline, we estimated hazard ratios (HRs) and 95% CIs using marginal Cox proportional hazards models with time from enrolment to the first event (SAM, MAM, or death). All 95% CIs were computed using the corrected robust estimator of variance instead of the more classic sandwich estimator, as it has been shown to underestimate the variance in a recurrent event data setting with a small number of independent geographical units (here, the village) [45]. Children contributed person-time to the analysis from enrolment until the first occurrence of the outcome (SAM, MAM, or death) or the end of follow-up.

To control for measured confounding given the relatively large number of potential confounders and limited events, we used propensity score adjustment [46]–[49]. Additional details of this method applied to the analysis of large-scale nutritional interventions have been published previously [11],[12]. Propensity score methods allow for the minimization of the effects of observed confounding when estimating the occurrence of an event using observational data. We estimated the propensity score on the entire sample using a logistic regression in which we estimated the probability of receiving a supplementation strategy given baseline characteristics that were *a priori* considered to be potential confounders or were associated with the supplementation strategy in univariate analyses at *p*<0.20 (Fisher's exact for qualitative variables, Fisher's ANOVA for normally distributed quantitative variables, and Mann-Whitney for others) [50]. Covariates included those in Table 3 with the exception of breastfeeding status as the overwhelming majority of children were breastfed at the time of evaluation. Scores were then computed for the full cohort using a logistic regression modeling this probability [51]. Indicators for quartile categories of the propensity score were included as independent variables in each outcome model. Each propensity score was divided into quartile categories using stratification. When considering the potential confounding effects of the covariates investigated here, there was no difference when using traditional multivariate versus propensity score adjustment. As the propensity score sample does not consist of independent observations, we used a marginal survival model with robust standard errors. Potential interactions were assessed with Cox models using partial likelihood ratio tests for malnutrition and mortality outcomes. For multiple pairwise comparisons, we used the Tukey's range test and for multiple comparisons within the same arm, the Holm–Bonferroni procedure was used [52],[53].

**Table 3:** Characteristics of children in each intervention group at inclusion August 2011.

Characteristics of Children	HQ-LNS/Cash	MQ-LNS/Cash	SC+/Cash	SC+/Food Ration	HQ-LNS	SC+	Cash	Total
	*N* = 542	*N* = 790	*N* = 539	*N* = 652	*N* = 710	*N* = 513	*N* = 430	*N* = 4,176
Age [median (IQR)]	15.6 (9.8–22.2)	15.0 (10.8–21.7)	15.9 (10.8–21.8)	16.3 (10.7–21.7)	15.3 (10.3–22.3)	17.4 (11.2–21.7)	14.6 (10.5–20.5)	15.7 (10.6–21.7)
Below 6 months [*n* (%)]	30 (5.5)	19 (2.4)	28 (5.2)	33 (5.1)	43 (6.1)	26 (5.1)	26 (6.1)	205 (4.9)
6–12 months [*n* (%)]	157 (29.0)	244 (30.9)	137 (25.4)	166 (25.4)	188 (26.5)	123 (23.9)	114 (26.5)	1,129 (27.0)
12–24 months [*n* (%)]	275 (50.7)	428 (54.2)	281 (52.1)	352 (54.0)	355 (50.0)	279 (54.4)	228 (53.0)	2,198 (52.6)
Above 24 months [*n* (%)]	79 (14.6)	93 (11.8)	92 (17.1)	97 (14.9)	123 (17.3)	85 (16.6)	56 (13.0)	625 (15.0)
Missing data [*n* (%)]	1 (0.2)	6 (0.8)	1 (0.2)	4 (0.6)	1 (0.1)	0 (0.0)	6 (1.4)	19 (0.4)
Male [*n* (%)]	291 (53.7)	351 (44.4)	266 (49.4)	294 (45.1)	361 (50.8)	252 (49.1)	186 (43.3)	2,001 (47.9)
Weight (kg) [mean ± SD]	8.0±1.4	7.9±1.2	8.0±1.2	7.8±1.2	7.7±1.3	7.9±1.3	7.7±1.3	7.9±1.3
Length (cm) [mean ± SD]	71.9±5.7	71.8±5.2	72.3±5.4	72.0±5.4	71.7±5.5	72.2±5.6	71.4±5.4	72.0±5.4
MUAC (cm) [mean ± SD]	13.4±1.1	13.4±1.1	13.3±1.0	13.2±1.1	13.1±1.1	13.2±1.2	13.2±1.1	13.2±1.1
Weight/height Z-score [mean ± SD]	−1.06±1.09	−1.18±1.08	−1.07±1.02	−1.24±1.08	−1.33±1.08	−1.26±1.19	−1.35±1.12	−1.21±1.02
MAM [*n* (%)]	137 (25.4)	194 (24.8)	144 (26.8)	189 (29.1)	228 (32.4)	140 (27.5)	119 (27.7)	1,151 (27.7)
SAM [*n* (%)]	22 (4.0)	37 (4.7)	18 (3.3)	37 (5.7)	44 (6.2)	37 (7.2)	35 (8.1)	230 (5.5)
Stunting [*n* (%)]	315 (58.1)	527 (66.7)	319 (59.2)	410 (62.9)	436 (61.4)	311 (60.6)	255 (59.3)	2,573 (61.6)
Severe stunting [*n* (%)]	141 (26.0)	227 (28.7)	138 (25.6)	194 (29.7)	214 (30.1)	161 (31.4)	128 (29.8)	1,203 (28.8)
Breastfeeding [*n* (%)]	345 (64.6)	507 (64.2)	362 (67.1)	420 (64.4)	458 (64.5)	299 (58.3)	295 (68.6)	2,686 (64.3)
Caregiver's age (mean [min-max])	26.6 [15–60]	27.3 [13–70]	27.2 [15–55]	28.1 [12–60]	26.7 [14–60]	27.2 [15–55]	26.2 [14–50]	27.1 [12–70]
Household size (mean [min-max])	5.4 [2–13]	5.6 [2–25]	5.3 [2–14]	5.5 [2–11]	5.3 [2–10]	5.5 [2–16]	5.3 [2–12]	5.4 [2–25]

IQR, interquartile range.

All data were collected on standardized forms and entered twice into EpiData version 2.1 (EpiData Association). Analyses were conducted using STATA version 12.1 (StataCorp).

## Results

A total of 4,514 children were enrolled for the first distribution in August 2011 after their caregivers gave informed consent at home. Caregivers of all eligible children agreed to participate. During the course of the study, we secondarily excluded 338 children after discovering that they did not meet the inclusion criteria (were not living in a study village or were not the child initially enrolled). Including all children who aged into (*n* = 4,685) or out (*n* = 710) of the study between August and November 2011, a total of 5,395 children from the 48 villages provided data for analysis. Over the study period, 20 children were withdrawn (Figure 1). The groups showed significant differences at baseline in the prevalence of MAM (*p* = 0.038), SAM (*p* = 0.008), stunting (*p* = 0.041) and the sex ratio (*p* = 0.003).

At the first distribution in August 2011, between 24.8% and 32.4% of children in each group were diagnosed with MAM, and 3.3% to 8.1% with SAM. About two-thirds of the children were stunted (LAZ<−2), with half of this group showing severe stunting (LAZ<−3). Two-thirds were being breastfed (Table 3).

Not all children received the same number of distributions, mostly linked to the fact that children were enrolled continuously over the study period. Overall, 69.1% of study children received four or five distributions (from 62.2% to 74.0% in the different intervention groups). More than 95% of children were present at all their planned distributions (from 93.3% to 98.8%, depending on intervention group). Over the study period, we registered 67 families, equally distributed across groups, with two included siblings in the study.

### Incidence of Acute Malnutrition

The lowest MAM incidences were observed in the SC+/cash group (3.33 per 100 child-months), followed by the HQ-LNS/cash group (3.73 per 100 child-months) and the MQ-LNS/cash group (4.28 per 100 child-months). This incidence was also the case for SAM. The lowest SAM incidence was found in the SC+/cash group (0.82 per 100 child-months), followed by the MQ-LNS/cash group (0.98 per 100 child-months), and the HQ-LNS/cash group (1.31 per 100 child-months). The cash-only group had the highest MAM incidence (7.97 per 100 child-months) and the HQ-LNS group the highest SAM incidence (2.24 per 100 child-months) (Table 4).

**Table 4:** Incidence of moderate acute malnutrition and severe acute malnutrition and mortality in each intervention group, August–December 2011.

Intervention Group	First Events/Total Child-Months Incidence and Mortality Rates per 100 Child-Months (95% CI)		
	MAM	SAM	Mortality
HQ-LNS/cash	56/1,501	28/2,132	5/2,621
	3.73 (2.87–4.85)	1.31 (0.91–1.90)	0.19 (0.08–0.46)
MQ-LNS/cash	91/2,128	31/3,150	3/3,990
	4.28 (3.48–5.25)	0.98 (0.69–1.40)	0.08 (0.02–0.23)
SC+/cash	47/1,410	17/2,069	5/2,594
	3.33 (2.50–4.43)	0.82 (0.51–1.32)	0.19 (0.08–0.46)
SC+/food ration	72/1,615	43/2,486	8/3,128
	4.46 (3.54–5.61)	1.73 (1.28–2.33)	0.26 (0.13–0.51)
HQ-LNS	94/1,612	58/2,589	22/3,128
	5.83 (4.76–7.14)	2.24 (1.73–2.89)	0.70 (0.46–1.07)
SC+	80/1,128	40/1,747	8/2,168
	7.09 (5.69–8.83)	2.29 (1.68–3.12)	0.37 (0.18–0.74)
Cash	91/1,141	33/1,846	14/2,304
	7.97 (6.49–9.79)	1.79 (1.27–2.51)	0.61 (0.36–1.03)

Tables 5 and S1 present the adjusted and unadjusted comparative risks of MAM and SAM as a function of the different strategies. Adjusted risk estimates were similar to those unadjusted. All strategies that combined a nutritious supplementary food with household support (cash or family food ration) showed a 2-fold lower MAM incidence compared with the cash only. In addition, the strategies including SC+/cash and MQ-LNS/cash had a lower incidence of SAM than the cash-only group (*p* = 0.011 and *p* = 0.005, respectively). The HQ-LNS/cash strategy had a lower MAM incidence than the HQ-LNS strategy (*p* = 0.001 and *p*<0.001, respectively) and the SC+/cash strategy had a lower MAM and SAM incidences than the SC+ only group (*p*<0.001). We did not detect any difference between the strategies relying on nutritious supplementary food-only (HQ-LNS and SC+) in terms of preventing MAM (*p* = 0.127) or SAM (*p* = 0.818). Moreover, we did not show any significant difference among strategies combining nutritious supplementary food and cash transfer for preventing MAM or SAM. However, the SC+/cash strategy was more preventive from SAM than SC+/food ration strategy (*p* = 0.015). We did not observe differences in MAM or SAM incidence between cash only and HQ-LNS (*p* = 0.056 and *p* = 0.533, respectively) or SC+ (*p* = 0.647 and *p* = 0.376, respectively) interventions.

**Table 5:** Adjusted comparative risk of moderate acute malnutrition and severe acute malnutrition and mortality as a function of different prevention strategies, August–December 2011.

Compared Strategies	MAM	*p*-Value	SAM	*p*-Value	Mortality	*p*-Value
	Adjusted HR (95% CI)^a^		Adjusted HR (95% CI)^a^		Adjusted HR (95% CI)^a^	
**Nutritious supplementary food only**						
SC+ vs. HQ-LNS (ref.)	1.27 (0.93–1.73)	0.127	1.06 (0.63–1.78)	0.818	0.55 (0.23–1.32)	0.182
**Cash only vs. nutritious supplementary food only**						
Cash vs. HQ-LNS (ref.)	1.39 (0.99–1.94)	0.056	0.84 (0.49–1.44)	0.533	0.81 (0.40–1.66)	0.571
Cash vs. SC+ (ref.)	1.09 (0.76–1.55)	0.647	0.78 (0.46–1.35)	0.376	1.74 (0.88–3.47)	0.114
**Cash only vs. nutritious supplementary food+household support**						
Cash vs. HQ-LNS/cash (ref.)	**2.30 (1.60–3.29)**	**<0.001**	1.34 (0.67–2.66)	0.406	**4.61 (1.60–13.33)**	**0.005**
Cash vs. SC+/cash (ref.)	**2.42 (1.39–4.21)**	**0.002**	**2.50 (1.24–5.05)**	**0.011**	3.08 (0.89–10.65)	0.076
Cash vs. MQ-LNS/cash (ref.)	**2.07 (1.52–2.83)**	**<0.001**	**2.12 (1.26–3.58)**	**0.005**	**7.77 (1.70–35.55)**	**0.008**
Cash vs. SC+/food ration (ref.)	**1.84 (1.29–2.60)**	**0.001**	1.15 (0.67–1.99)	0.604	2.27 (0.69–7.44)	0.175
**Nutritious supplementary food only vs. nutritious supplementary food+household support**						
HQ-LNS vs. HQ-LNS/cash (ref.)	**1.84 (1.35–2.51)**	**<0.001**	1.69 (0.88–3.25)	0.115	**5.72 (1.96–16.72)**	**0.001**
SC+ vs. SC+/cash (ref.)	**2.53 (1.47–4.35)**	**0.001**	**3.13 (1.65–5.94)**	**<0.001**	2.15 (0.56–8.30)	0.266
SC+ vs. SC+/food ration (ref.)	**1.72 (1.26–2.35)**	**0.001**	1.46 (0.93–2.29)	0.102	1.51 (0.40–5.72)	0.540
**Nutritious supplementary food+household support**						
MQ-LNS/cash vs. HQ-LNS/cash (ref.)	1.09 (0.80–1.48)	0.604	0.63 (0.32–1.21)	0.165	0.57 (0.11–3.06)	0.511
SC+/cash vs. HQ-LNS/cash (ref.)	0.86 (0.48–1.55)	0.626	0.53 (0.24–1.16)	0.165	1.37 (0.32–5.87)	0.675
SC+/cash vs. MQ-LNS/cash (ref.)	0.69 (0.42–1.13)	0.143	0.84 (0.44–1.60)	0.591	2.23 (0.42–11.84)	0.345
SC+/food ration vs. SC+/cash (ref.)	1.49 (0.88–2.52)	0.140	**2.16 (1.16–4.02)**	**0.015**	1.43 (0.31–6.74)	0.648

Bold indicates statistical significance (*p*<0.05).

a From marginal Cox proportional hazards models, where the outcome variable is time until first event. Predicators in the adjusted model included supplementation strategy and indicators for quartiles of the estimated propensity score. We estimated the propensity score on the entire sample using a logistic regression in which we estimated the probability of receiving a supplementation strategy given baseline characteristics that were *a priori* considered to be potential confounders (Table 3) or were associated with the supplementation strategy in univariate analyses at *p*<0.20. Breastfeeding was not included as a potential confounder as the overwhelming majority of children were breastfed at the time of evaluation.

### Mortality

We recorded 65 deaths across all groups. Mortality varied from 0.08 per 100 child-months (MQ-LNS/cash group) to 0.70 per 100 child-months (HQ-LNS group) (Table 4). The deaths occurred mainly between August and October 2011. The average age of children who died was 16.0 months (standard deviation [SD] = 6.9 months; min = 6.3; max = 38.9). The average period between the first attended distribution and death was 69.2 days (SD = 35.9 days; min = 21; max = 162). Of the 65 deaths recorded, 59 were documented by verbal autopsy. The cause of the remaining six deaths could not be documented because the families were either travelling or had moved. A total of 21 documented deaths (36%) occurred at home and 36 (61%) in a health facility. Two deaths (3%) occurred on the road between home and health facility. A total of 57/59 families accessed health care prior to the death of their child. The most frequent cause of death reported by the mothers was fever (76%), which may likely to have been caused by malaria as August to October is the malaria peak in Niger, followed by gastroenteritis (14%).

A comparison across intervention groups indicated that mortality in the HQ-LNS/cash and MQ-LNS/cash strategies was greatly reduced compared to the cash-only strategy (*p* = 0.005 and *p* = 0.008, respectively). Despite a non-significant difference, there was a suggestive trend in mortality reduction for the SC+/cash group compared to cash-only group (*p* = 0.076). The HQ-LNS/cash strategy showed lower mortality than the HQ-LNS strategy (*p* = 0.001). However, we did not detect any difference in mortality between the SC+/cash and SC+ strategies, or between SC+/food ration and SC+ strategies (Tables 5 and S1).

### Use and Consumption of Nutritious Supplementary Foods

The majority of nutritious supplementary food rations (77%–93%) distributed between August and November 2011 was reported to have been consumed within the nuclear family of the beneficiary child (Table 6), according to information provided by caregivers. Less than 10% of the rations were shared within the wider household (i.e., beyond members of the nuclear family). Around one-third of households (29%) were composed of at least two beneficiary nuclear families. SC+ distributed with the family food ration was the most stored within the household (around 0.6% total ration distributed meaning around 37 g per household per month). The proportion of rations consumed within the nuclear family was lower among the intervention groups that received household support (HQ-LNS/cash versus HQ-LNS only, *p*<0.001; SC+/cash versus SC+, *p*<0.001; SC+/family food ration versus SC+ only, *p*<0.001). The households that received SC+/cash gave away a higher proportion of the rations to people outside the household than did either the household that received HQ-LNS/cash (*p*<0.001) or MQ-LNS/cash (*p*<0.001). There was no difference between the SC+ and HQ-LNS groups with respect to the proportion of rations kept within the household (*p* = 0.940) and that not kept within the household (*p* = 0.120).

**Table 6:** Proportion of total ration of nutritious supplementary foods used within households, August–November 2011.

Use of Nutritious Supplementary Foods within Households	HQ-LNS/Cash	MQ-LNS/Cash	SC+/Cash	SC+/Food Ration	HQ-LNS	SC+
**Kept within households**	92.6	92.2	85.5	93.4	95.8	95.9
Consumed within nuclear family	86.6	84.2	76.6	87.5	92.9	91.7
Consumed within household, beyond nuclear family	5.9	7.8	8.7	5.3	2.8	4.0
Stored	0.1	0.2	0.2	0.6	0.1	0.2
**Not kept within households**	7.4	7.8	14.5	6.6	4.3	4.1
Given away outside household	7.4	7.7	14.4	6.6	2.9	4.0
Sold/exchanged	0.0	0.1	0.1	0.0	1.3	0.1

Proportion of nutritious supplementary foods kept or not kept within the household as reported by caregivers and additional detail in each of these categories (rows). Columns represent each of the strategies. The sum in each column of “kept within households” and “not kept within households” totals 100.

Of the food supplements consumed within the household, the targeted child was the sole consumer in 30%–51% of households depending on the intervention group (Table 7). SC+ distributed with the family food ration was more consumed by the targeted child only compared to SC+/cash transfer (*p*<0.001) or alone (*p*<0.001). When received without household support, SC+ and HQ-LNS were most consumed within the household and frequently shared within members. SC+ was reported to be consumed more frequently by women (mothers, pregnant women, or breast-feeding mothers) than the LNS (SC+ versus HQ-LNS *p*<0.001), while LNS was more frequently shared with other children under 5 years (SC+ versus HQ-LNS *p*<0.001).

**Table 7:** Proportion of caregivers responding concerning consumption of distributed nutritious supplementary foods within households from August–November 2011.

Main Consumers of Nutritious Supplementary Foods within Households	HQ-LNS/Cash	MQ-LNS/Cash	SC+/Cash	SC+/Food Ration	HQ-LNS	SC+
Targeted child only	30.0	34.9	36.9	50.8	34.8	34.6
All children <5 years	56.0	61.5	30.8	24.8	63.9	59.3
Mothers, breastfeeding mothers, or pregnant women	19.5	19.0	31.3	18.2	11.3	22.1
Children >5 years and adults	26.4	16.0	27.0	15.6	13.3	16.3

The categories are non-exclusive and represent the proportion of caregivers responses with respect to consumption. A caregiver could respond positively to the last three categories.

## Discussion

### Comparative Effectiveness of the Interventions

This prospective interventional study assessed the effectiveness of several nutritional and household support strategies used to prevent acute malnutrition in 6–23 month old children comparing seven different interventions in the same region and under standardized conditions for 5 months partly overlapping the annual hunger gap. We showed that large-scale distributions combining a nutritious supplementary food with cash transfer that increased household purchasing power are potentially more effective at preventing acute malnutrition and mortality than strategies relying on either cash transfer or nutritious supplementary food alone.

The incidence of MAM in groups receiving both nutritious supplementary food (SC+, MQ-LNS, HQ-LNS) and cash was half that seen in the group that received cash only, although the latter received €5 (US$7) more per month to cover the additional cost of buying locally available nutritious foods for the targeted child. This finding suggests that direct provision of nutritious supplementary food for a young child confers greater benefit than a comparable amount as cash in terms of “nutrition security” for young children, at least among households that received a cash transfer that bolstered household purchasing power. The effect of distributing nutritious food supplements (HQ-LNS or SC+) without household support was roughly the same as providing cash only (€43/month [US$59/month]) in terms of preventing MAM, SAM, and mortality, while the cost of the nutritious food supplements (€3.5–€7.3/month [US$4.8–US$10.1/month]), excluding transport, storage, and distribution) was considerably lower than the value of the cash provided.

In March 2012 (3 months after the last cash transfer), a qualitative study was conducted by an independent team among mothers of children who received distributions in two villages from each intervention group. (Langendorf et al., unpublished). This qualitative study consisted of focus group discussions and individual interviews and included a total of 212 participants. In the selected villages having received cash transfers (only or added to supplementary foods), the discussions were focused on the use of distributed cash. It showed that most caretakers reported spending approximately 20% of the money received to purchase nutritious foods earmarked for the child included in the study (e.g., milk, chicken, fish, liver, eggs, beans, oranges, bananas). Thus, households used their additional resources to improve the nutritional quality of the diet of young children, which may reflect and reinforce the importance of the nutrition information sessions provided by the study team to mothers and to the community.

However, children of households that were beneficiaries of a strategy with cash and a nutritious supplementary food were better protected against MAM, SAM, and mortality, than children of households that received only cash, even though they received more cash (43 instead of €38/month [US$52 instead of US$59/month]), and they reported buying specific locally available nutritious foods for the children. In other words, it seems that the nutritional value of the diet that depended on locally available foods, as purchased by households that could afford these foods and were informed about good choices and good child feeding practices, was lower than that of children that received a nutritious supplementary food in addition to their regular diet. Considering that the study was conducted in a food insecure area and during the lean season, this finding is unsurprising, even when household purchasing power is improved, availability of nutritious foods is limited. In fact, for children aged 6–23 months, it is very difficult to meet nutrient requirements when their diet includes very few animal source or fortified foods, and fortified foods are most cost-effective at meeting nutrient requirements compared with natural foods [54]–[56]. In some contexts, provision of nutritious supplementary foods may be the only feasible strategy for preventing malnutrition among the youngest children.

It is important to note that within nuclear families, reports of consumption of nutritious supplementary foods were lower when accompanied by cash transfer. This result suggests that adding cash transfer resulted in less nutritious supplementary food being consumed by the targeted child but emphasizes the possible nutritional benefit of completing the targeted children diet with specific locally available nutritious foods, affordable thanks to cash transfer. As another kind of household support, adding family food ration to SC+ showed improved prevention of MAM and mortality than SC+ alone. This finding could be explained by the fact that SC+, when protected by family food ration is shared less within the household and more frequently consumed by the targeted child only. This finding supports the role of family food rations to limit intra-household sharing of the child's nutritious food ration [57].

Our findings suggest that the choice of a specific type of nutritious supplementary food among those recommended by the World Food Programme (WFP), i.e., whether HQ or MQ-LNS or SC+, was less important in terms of prevention from acute malnutrition, as we did not detect differences in effectiveness between the HQ-LNS/cash, MQ-LNS/cash, or SC+/cash strategies, or between HQ-LNS and SC+ strategies. The finding that the incidence of acute malnutrition was not correlated with the caloric value of the nutritious supplementary foods (820, 500 or 250 kcal/day) may point to their micronutrient density as an important factor, and to how much of each food typically gets consumed by the targeted child. MQ-LNS has a higher micronutrient density than HQ-LNS and SC+. However, the daily ration of the different products provides roughly the same absolute amount of micronutrients. The daily ration of 200 g SC+ is equivalent to 1 litre of porridge, considerably more than the 92 g/day of HQ-LNS or 46 g/day of MQ-LNS. Thus, children need to eat much less MQ-LNS than HQ-LNS or SC+ to absorb an equivalent amount of micronutrients. As a result, considering food supplement sharing among household members, children who consumed partial ration of MQ-LNS were likely to be provided with a roughly similar amount of micronutrients than were children consuming partial ration of SC+ or HQ-LNS.

### Cost Estimates

Cost estimates for large-scale programs are fraught with difficulty and often not evaluated prospectively. However, information on program costs is a key indicator for public health decision-makers and program managers. Here, although we did not assess costs prospectively (especially programmatic costs) as costing was not an objective of this study, we present some estimates. In this study, purchase prices for a monthly ration per child of MQ-LNS, SC+, or HQ-LNS were €3.52 (US$4.86), €5.70 (US$7.86), and €7.27 (US$10.03), respectively. In order to include operational costs (transport, handling, storage, distribution), the purchase price was inflated by 50% following WFP average estimates. Thus, the overall average cost of nutritious supplementary food-only strategies was about €8.25 (US$11.38)/child/month (€5.28 [US$7.28] with MQ-LNS; €8.55 [US$11.79] with SC+; €10.91 [US$15.05] with HQ-LNS). These figures are consistent with a recent report of the WFP-Niger [58] and informal data from MSF activities in Mali.

According to another direct cash transfer program conducted in Niger, overhead and implementation costs were estimated at 22% of overall budget [40]; in other contexts, this proportion was estimated at 10%–15% [59]. In consequence, cash distributions necessitate additional means. Therefore, in our study, cash transfer-only strategy (€43/household/month = US$59/household/month) come at a higher cost than distribution of nutritious supplementary foods (around €8/child/month = US$11/child/month including operational costs) to achieve the same outcome in terms of malnutrition prevention. However, we cannot assume that effect on malnutrition prevention will deteriorate at lower levels of cash transfer than €43 (US$59) per beneficiary per month.

### Limitations of This Study

This study has several limitations. First, we did not collect data on all possible factors that could have influenced the incidence of malnutrition and mortality. However, we did not notice important events during the study period (e.g., disease outbreaks, flooding) that specifically affected the included villages. We adjusted our estimates on known baseline characteristics, but differences, which we did not quantify, may remain between intervention groups, villages, families, and children and may affect the impact of the interventions. However, by conducting the study in one geographic location and setting specific criteria for eligibility of villages, the risk of unquantified differences differentially affecting the impact of specific interventions was reduced.

We implemented several procedures to minimize measurement biases, including staff training, close supervision of staff, systematic double measurement, and external quality control. We also used a local calendar of events to limit bias relating to misclassification of children's age. The results obtained on use and consumption of the supplementary foods in a household may have been biased by social desirability.

Although village or even individual randomization would be ideal, we chose a prospective interventional study with randomization of groups when possible for several reasons. First, taking into consideration the synergistic effect of food or cash distributions within a large community (groups of villages) represents the closest possible scenario to the actual implementation of large-scale distributions, and thereby maximizes applicability of the study's results and lessons learned. Second, concerns about contamination between individuals and villages, often present with respect to nutritional interventions, have been a significant barrier in previous studies leading to the need to consider alternate designs. Studies exploring the potential impact of cash transfers also need to be considerate of context and participants and some designs may not be possible despite their explanatory benefits.

The 4-month length of follow-up in this study was too short to permit drawing firm conclusions about young children's growth or acute malnutrition relapse and recovery as planned in the study protocol. However, it is important to recognize that nutritional interventions in crises are usually implemented over a short duration (3–6 months) as recommended by international guidance. As a result, in order to provide meaningful information to guide operational decision-making, results over relatively short periods (months) are most pertinent. Long-term follow-up of children receiving supplementation may respond to additional questions about the potential benefits of different strategies over time, stunting, for example and in our case, will be accomplished through 10 months of additional follow-up in a sub-cohort.

The study period did not overlap exactly with the hunger gap in the study area, which varies annually depending on the rains, at which point malnutrition generally decreases. Thus, the incidences of malnutrition may have been different (probably higher) if the study had been conducted during the complete hunger gap. It was not possible to start the study earlier (as initially intended) because of logistical constraints linked to the novelty of the different interventions and negotiations between the multiple operating partners. However, when analyses are restricted to the first 3 months of the study (the hunger gap), qualitative interpretation of the results remain the same.

### Generalizability of These Findings

The outcomes of nutritional interventions like these are highly dependent on the health and socio-economic environment of the study area. In this study setting, high coverage of comprehensive, free pediatric primary care in functional health structures (including TFP) guaranteed timely care for all enrolled children who were ill and/or developed SAM. Therefore, the effect size of these interventions may be different in a setting with less access to comprehensive primary care.

In this case, the study population was familiar with nutritious supplementary foods targeting young children. Many therapeutic nutritional programs and large-scale distributions of nutritious foods have been implemented in the area over the past several years. Perhaps similarly, care takers reported high acceptability of all nutritious supplementary foods (according to a parallel qualitative study and in agreement with previous studies [60],[61]). Therefore, the results may be different in other settings where intervention adherence or product acceptability would be lower. Furthermore, the impact of the cash transfer strategy very much depends on what difference the cash can make, in terms of household food security, dietary diversity, access to health care, hygiene, time spent on caring etc., which is highly context dependent.

However, in order to maximize applicability and reproducibility of the study's results to other community settings, this study used large-scale distribution methods that were as close as possible to FORSANI/MSF protocols and practice. We did not attempt to control parameters that may influence beneficiaries' behavior (and, in turn, study results), such as the quantity of the nutritious supplementary food actually consumed by target children or the amount of cash actually spent to feed them. Such control is rarely operationally feasible within large-scale interventions in emergency contexts, so this approach would have been less informative in terms of the real-world effectiveness of these interventions in difficult contexts.

While strategies combining nutritious food supplements for the youngest child with cash to support the household seem to be most efficacious in preventing acute malnutrition and possibly mortality, they are also very costly because of the high amount of cash distributed. Cash only and nutritious supplementary foods only were equally effective, but the latter cost less. Therefore, prevention of malnutrition and mortality in high burden contexts may best be done by distributing nutritious supplementary foods in the form of blanket distribution (i.e., distribution to all children under 2 years without targeting specific groups) at a relatively large scale. In addition, concurrent distribution of cash should be considered to be carried out in a more targeted manner, when feasible to identify households according to socio-economic status, to especially support the most vulnerable households. The latter would be a social safety net type intervention, for all vulnerable households, which could be made more nutrition sensitive by adding special nutritious foods for the most vulnerable members such as children under 2 years old and pregnant and lactating women.

Formal cost-effectiveness studies would be required to look at what option would be more feasible, i.e., smaller amounts of cash transfers to add to distribution of nutritious supplementary food that could still be beneficial in terms of additional reduction of the risk of malnutrition and mortality. Furthermore, it would be relevant to compare impacts of interventions for preventing malnutrition and mortality on additional outcomes such as morbidity, childhood cognitive and motor development, and household food security, over the medium and long term.

### Conclusions

As prevention of malnutrition is crucial in countries facing recurrent nutritional crises and where financial resources are limited, our study results suggest that blanket distribution of nutritious supplementary foods to children under 2 years of age, associated with targeted cash transfer to the most vulnerable households, could be a cost-effective strategy in the short term. These results can be used to guide donor decisions, particularly in the high-burden Sahel region of sub-Saharan Africa, as well as the operational strategies of agencies in their programmatic response to crises. However, additional rigorous research is vital to evaluate the effectiveness of these strategies, particularly concerning the choice of supplementary foods and other nutritional interventions in diverse settings. Ensuing additional studies exploring supplementary foods, cash, and other modalities are essential given the overall burden of childhood acute malnutrition in sub-Saharan Africa. Although studies of this type present unique methodological challenges, without them, the evidence base to draw on for operational guidance will remain sparse.

## Supporting Information

Figure S1 **Map of Niger showing Maradi region (pink) and Madarounfa district (red) where the study was implemented in August 2011.** Credit: adapted from http://commons.wikimedia.org/wiki/File%3AMaradi_in_Niger.svg. This figure is licensed under the Creative Commons Attribution-Share Alike 2.0 Generic license (http://creativecommons.org/licenses/by-sa/2.0/legalcode).
(TIF)

Table S1 **Unadjusted comparative risk of moderate acute malnutrition (MAM) and severe acute malnutrition (SAM) and mortality as a function of different prevention strategies, August–December 2011.**
(DOCX)

Protocol S1 **Study protocol (in French).**
(PDF)

Checklist S1 **CONSORT checklist.**
(DOC)

## Abbreviations

FCFA: franc de la Communauté Financière Africaine
FORSANI: Forum Santé Niger
HQ-LNS: high-quantity lipid-based nutrient supplement
HR: hazard ratio
LAZ: length-for-age Z-score
LNS: lipid-based nutrient supplement
MAM: moderate acute malnutrition
MQ-LNS: medium-quantity lipid-based nutrient supplement
MSF: Médecins Sans Frontières
MUAC: mid-upper arm circumference
SD: standard deviation
SAM: severe acute malnutrition
SC+: Super Cereal Plus
TFP: therapeutic feeding program
WHZ: weight-for-height Z-score
WLZ: weight-for-length Z-score
