# Supplementary figures and images for: Preventing Acute Malnutrition among Young Children in Crises: A Prospective Intervention Study in Niger
Source: PLoS Med. 2014 Sep 2;11(9):e1001714. doi: 10.1371/journal.pmed.1001714 (PMC4152259; doi:10.1371/journal.pmed.1001714)

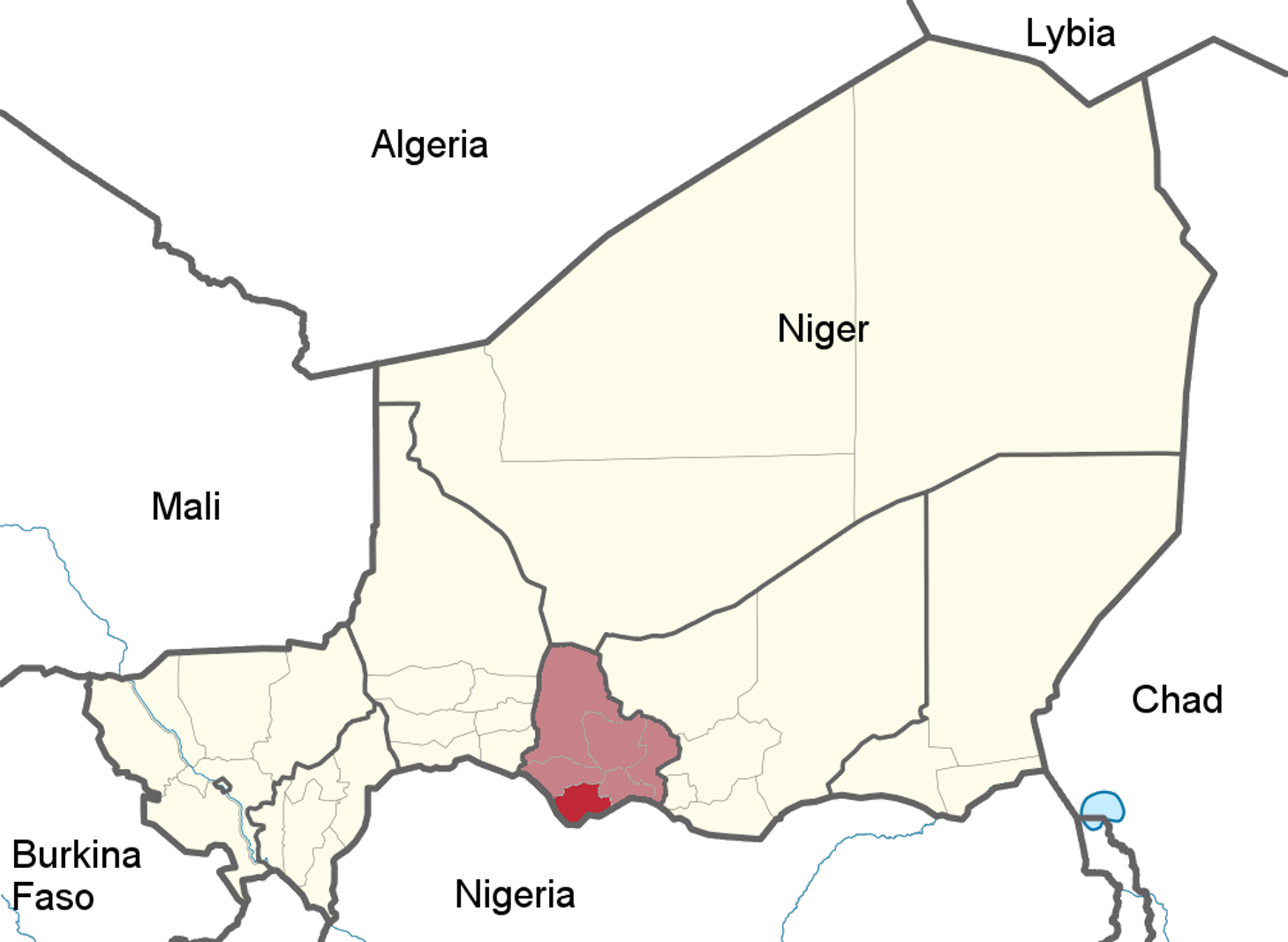

Supplement: Figure S1 — Map of Niger showing Maradi region (pink) and Madarounfa district (red) where the study was implemented in August 2011. Credit: adapted from http://commons.wikimedia.org/wiki/File%3AMaradi_in_Niger.svg. This figure is licensed under the Creative Commons Attribution-Share Alike 2.0 Generic license (http://creativecommons.org/licenses/by-sa/2.0/legalcode). (TIF) [file pmed.1001714.s001.tif]
