# Supplementary material for: Preventing Acute Malnutrition among Young Children in Crises: A Prospective Intervention Study in Niger
Source: PLoS Med. 2014 Sep 2;11(9):e1001714. doi: 10.1371/journal.pmed.1001714 (PMC4152259; doi:10.1371/journal.pmed.1001714)
Supplement: Table S1 — Unadjusted comparative risk of moderate acute malnutrition (MAM) and severe acute malnutrition (SAM) and mortality as a function of different prevention strategies, August–December 2011. (DOCX) [file pmed.1001714.s002.docx]

Table S1. Unadjusted comparative risk of Moderate Acute Malnutrition (MAM) and Severe Acute Malnutrition (SAM) and mortality as a function of different prevention strategies, August-December 2011

|  | **MAM** |  | **SAM** |  | **Mortality** |  |
| --- | --- | --- | --- | --- | --- | --- |
|  | **Unadjusted HR (95% CI)** | **P** | **Unadjusted HR (95% CI)** | **P** | **Unadjusted HR (95% CI)** | **P** |
| **Nutritious supplementary food only** | | | | | | |
| SC+ vs. HQ-LNS (ref.) | 1.30 (0.93 – 1.62) | 0.147 | 1.01 (0.62 – 1.64) | 0.965 | 0.53 (0.21 – 1.29) | 0.161 |
| **Cash only vs. nutritious supplementary food only** | | | | | | |
| Cash vs. HQ-LNS (ref.) | 1.32 (0.97 – 1.80) | 0.073 | 0.79 (0.45 – 1.39) | 0.418 | 0.76 (0.38 – 1.54) | 0.450 |
| Cash vs. SC+ (ref.) | 1.08 (0.76 – 1.53) | 0.679 | 0.79 (0.45 – 1.36) | 0.388 | 1.45 (0.60 – 3.50) | 0.406 |
| **Cash only vs. nutritious supplementary food + household support** | | | | | | |
| Cash vs. HQ-LNS/cash (ref.) | **2.51 (1.68 – 3.76)** | **<0.001** | 1.46 (0.73 – 2.92) | 0.730 | **4.02 (1.39 – 11.64)** | **0.010** |
| Cash vs. SC+/cash (ref.) | **3.04 (1.74 – 5.30)** | **<0.001** | **2.59 (1.26 – 5.33)** | **0.010** | 3.41 (0.96 – 12.19) | 0.059 |
| Cash vs. MQ-LNS/cash (ref.) | **2.13 (1.57 – 2.88)** | **<0.001** | **2.21 (1.25 – 3.92)** | **0.007** | **8.05 (1.77 – 36.61)** | **0.007** |
| Cash vs. SC+/food ration (ref.) | **1.85 (1.32 – 2.59)** | **<0.001** | 1.13 (0.66 – 1.95) | 0.653 | 2.15 (0.65 – 7.11) | 0.209 |
| **Nutritious supplementary food only vs. nutritious supplementary food + household support** | | | | | | |
| HQ-LNS vs. HQ-LNS/cash (ref.) | **1.90 (1.35 – 2.67)** | **<0.001** | 1.84 (0.97 – 3.48) | 0.073 | **5.28 (1.67 – 16.67)** | **0.005** |
| SC+ vs. SC+/cash (ref.) | **2.82 (1.64 – 4.85)** | **<0.001** | **3.30 (1.71 – 6.36)** | **<0.001** | 2.35 (0.59 – 9.34) | 0.225 |
| SC+ vs. SC+/food ration (ref.) | **1.72 (1.26 – 2.34)** | **0.001** | 1.44 (0.92 – 2.27) | 0.112 | 1.48 (0.39 – 5.62) | 0.563 |
| **Nutritious supplementary food + household support** | | | | | | |
| MQ-LNS/cash vs. HQ-LNS/cash (ref.) | 1.18 (0.85 – 1.65) | 0.604 | 0.66 (0.34– 1.29) | 0.153 | 0.50 (0.09 – 2.79) | 0.429 |
| SC+/cash vs. HQ-LNS/cash (ref.) | 0.83 (0.47 – 1.47) | 0.518 | 0.56 (0.25 – 1.25) | 0.201 | 1.18 (0.26 – 5.33) | 0.831 |
| SC+/cash vs. MQ-LNS/cash (ref.) | 0.70 (0.42 – 1.16) | 0.165 | 0.85 (0.45 – 1.62) | 0.626 | 2.36(0.45 – 12.48) | 0.313 |
| SC+/food ration vs. SC+/cash (ref.) | 1.64 (0.97 – 2.78) | 0.063 | **2.29 (1.21 – 4.33)** | **0.010** | 1.59 (0.33 – 7.68) | 0.567 |

HQ-LNS, high-quantity lipid-based nutrient supplement; MQ-LNS, medium-quantity lipid-based nutrient supplement; SC+, Super Cereal Plus; HR, hazard ratio; CI, confidence interval.
