# Supplementary material for: Preventing Acute Malnutrition among Young Children in Crises: A Prospective Intervention Study in Niger
Source: PLoS Med. 2014 Sep 2;11(9):e1001714. doi: 10.1371/journal.pmed.1001714 (PMC4152259; doi:10.1371/journal.pmed.1001714)
Supplement: Protocol S1 — Study protocol (in French). (PDF) [file pmed.1001714.s003.pdf]

Centre Collaborateur de l'OMS
pour la Recherche en Epidémiologie
et la Réponse aux Maladies Emergentes

TELEPHONE : 00 33 (0)1 40 21 28 48
FAX: 00 33 (0)1 40 21 28 03
E-MAIL : EPIMAIL@EPICENTRE.MSF.ORG
WEB: HTTP://WWW.EPICENTRE.MSF.ORG

ASSOCIATION LOI 1901.

Evaluation de différentes stratégies de distributions préventives de la malnutrition au Niger

---

District de Madarounfa

Région de Maradi

Juin 2011 – Novembre 2012

Juin 2011

**Titre de l'étude**	Evaluation de différentes stratégies de distributions préventives de la malnutrition au Niger
**Promoteur**	Epicentre
**Principal investigateur**	Céline Langendorf<br>Epicentre<br>Tel : +33 1 40 21 55 12<br>E-mail : celine.langendorf@epicentre.msf.org
**Co-investigateurs**	Grais Rebecca, Epicentre<br>E-mail : rebecca.grais@epicentre.msf.org<br><br>Kanta Issa Malam, Forsani<br>E-mail : forsani_org@yahoo.fr<br><br>Mamaty Abdul-Aziz, Epicentre, Maradi<br>E-mail : abdul-aziz.mamaty@epicentre.msf.org<br><br>Manzo Mahamane Laouali, DRSP, Maradi, Niger<br>E-mail : manzolic@yahoo.fr
**Equipe de développement du protocole**	Ballo Moise, Programme Alimentaire Mondial Niger<br>Boureima Hassane, District sanitaire de Madarounfa<br>de Pee Saskia, Programme Alimentaire Mondial<br>Fuli Rachel, Programme Alimentaire Mondial Niger<br>Guero Maïmouna, Direction de la Nutrition, MSP Niger<br>Rigal Jean, Médecins Sans Frontières-OCP<br>Rodrigues Marie-Noëlle, Médecins Sans Frontières-OCP<br>Roederer Thomas, Epicentre<br>Toure Mandjouh, Epicentre, Niger
**Partenaires opérationnels**	Programme Alimentaire Mondial<br>FORSANI<br>ASUSU
**Partenaires financiers**	Médecins Sans Frontières<br>Programme Alimentaire Mondial

Table des matières

1. CONTEXTE 6
1.1. Malnutrition dans les pays en developpement 6
1.2. Malnutrition au Niger 7
1.3. De la prise en charge curative a la prevention de la malnutrition 8
1.4. Description des interventions en 2011-2012 11
2. RATIONNEL 13
3. OBJECTIFS 14
3.1. Objectif principal 14
3.2. Objectifs secondaires 15
4. METHODES 15
4.1. Type d'etude 15
4.2. Groupes d'intervention 16
4.3. Zone de l'etude 17
4.4. Population cible 17
4.5. Criteres d'inclusion des enfants dans la cohorte 18
4.6. Criteres d'exclusion des enfants dans la cohorte 18
4.7. Echantillonnage : nombre de sujets 19
4.8. Criteres de selection des villages 19
4.9. Attribution du groupe d'intervention 20
4.10. Criteres de jugement 20
4.11. Definitions 21
5. DEROULEMENT DE L'ETUDE 23
5.1. Phase preparatoire 23
5.2. Inclusion des villages 23
5.3. Selection des enfants 24
5.4. Inclusion des enfants 24
5.5. Distributions preventives 25
5.6. Frequence de suivi des enfants 26
5.7. Donnees collectees 26
6. TRAITEMENT DES DONNEES 28
6.1. Saisie des donnees 28
6.2. Analyse des donnees 28
6.3. Plan d'analyse 28
7. LIMITES ATTENDUES 29
8. CONSIDERATIONS ETHIQUES 30
8.1. Autorisations 30
8.2. Consentement eclaire 30
8.3. Confidentialite 31

8.4. Archivage 31
8.5. Benefices pour le patient 31
9. CALENDRIER DE L'ETUDE 31
10. PARTENARIAT 33
11. RESSOURCES 33
11.1. Ressources humaines 33
11.2. Ressources logistiques 34
11.3. Ressources financieres 34
12. COMMUNICATION DES RESULTATS 34
13. REFERENCES 35
ANNEXES 38

Abréviations

ARC	Assistant de Recherche Clinique
ASPE	Aliments de Supplémentation Prêts à l'Emploi
ATPE	Aliments Thérapeutiques Prêts à l'Emploi
CCSAP	Cellule de Coordination du Système d'Alerte Précoce
CIOMS	Conseil des Organisations Internationales des Sciences Médicales
CRENAS	Centre de Réhabilitation et d'Education Nutritionnelle Ambulatoire Sévère
CRENI	Centre de Réhabilitation et d'Education Nutritionnelle Intensive
CSB	Corn-Soy Blend
CSI	Centre de Santé Intégré
DRSP	Direction Régionale de la Santé Publique
FAO	Organisation des Nations Unies pour l'Alimentation et l'Agriculture
FORSANI	Forum Santé Niger
IC	Intervalle de confiance
LNS	Suppléments nutritionnels à base de lipides
Kcal	Kilocalories
INS	Institut National de la Statistique
MAG	Malnutrition Aiguë Globale
MAS	Malnutrition Aiguë Sévère
MAM	Malnutrition Aiguë Modérée
MSF	Médecins Sans Frontières
MSF-OCP	Médecins Sans Frontières Operational Centre Paris
ONG	Organisation Non Gouvernementale
ONU	Organisation des Nations Unies
OMD	Objectif du Millenium pour le Développement
OMS	Organisation Mondiale de la Santé
PAM	Programme Alimentaire Mondial
PB	Périmètre brachial
P/T	Indice Poids pour Taille
SAP	Système d'Alerte Précoce
UNICEF	Fonds des Nations Unies pour l'Enfance

1. Contexte

1.1. Malnutrition dans les pays en développement

Selon les dernières estimations de l'Organisation des Nations Unies pour l'Alimentation et l'Agriculture (FAO), le nombre d'habitants sous-alimentés dans le monde est estimé à 1 milliard. Selon les dernières estimations du Fonds des Nations Unies pour l'Enfance (UNICEF), le nombre d'enfants de moins de 5 ans souffrant d'un retard de croissance dans les pays en développement s'élève à 195 millions, soit un enfant sur trois [1]. Plus d'un quart des enfants de moins de 5 ans souffrirait d'insuffisance pondérale, dans les pays en développement. Dans ces pays, 13% des enfants de moins de 5 ans seraient malnutris et 5% sévèrement malnutris (soit 26 millions d'enfants). La grande majorité de ces enfants (80%) est répartie dans 20 pays.

La malnutrition est reconnue comme le facteur de risque le plus important de morbidité et de mortalité, et constitue un problème de santé publique majeur (1). Plusieurs études estiment que la malnutrition est la cause sous-jacente de 55% de l'ensemble des décès survenant dans le monde parmi les enfants de moins de cinq ans [2,3]**.** Chaque année, la malnutrition contribue au décès de 8 millions d'enfants [1]. Il est reconnu que le risque de décès est supérieur chez les enfants sévèrement malnutris. Néanmoins, si l'on considère le taux élevé de mortalité due à la malnutrition modérée au regard de sa haute prévalence dans le monde, le nombre des décès attribuables à la malnutrition modérée est supérieure à celui attribuable à la malnutrition sévère. Ces considérations placent la prévention de la malnutrition des enfants comme une priorité majeure des actions de lutte contre la mortalité des enfants [4].

Chaque année, la Malnutrition Aiguë Sévère (MAS) - définie par un rapport poids / taille <-3 z-score (standards OMS 2005) et/ou présence d'œdèmes bilatéraux nutritionnels - touche plus de 20 millions d'enfants dans le monde. La Malnutrition Aiguë Modérée (MAM) - définie par un rapport poids / taille (P/T) entre -3 et -2 z-score - quant à elle, concerne plus de 36 millions d'enfants avec une probabilité d'évoluer vers une MAS [5].

En 2000, le Sommet des Nations Unies a accordé une place prioritaire à l'éradication de la faim. Le premier Objectif du Millenium pour le Développement (OMD) consiste à réduire de moitié, entre 1990 et 2015, l'extrême pauvreté et la proportion des personnes souffrant de la faim. Malgré une diminution du nombre de personnes sous alimentées dans le monde entre 2009 et 2010, il reste plus élevé qu'avant les récentes crises économiques. Des progrès ont été accomplis dans certaines régions mais le rythme de diminution montre d'importantes disparités. En Afrique subsaharienne, la prévalence de la sous-alimentation est la plus élevée au monde (30%). Bien que celle-ci se soit légèrement infléchie au cours des dernières années, le nombre absolu des personnes sous-alimentées continue à croître en raison de l'augmentation de la population [6].

Le quatrième OMD est la réduction de deux tiers de la mortalité des enfants de moins de 5 ans entre 1990 et 2015. Des efforts considérables pour atteindre cette cible ont déjà été entrepris. Au plan mondial, le nombre total de décès d'enfants de moins de 5 ans est passé de 12,5 millions en 1990 à 8,8 millions en 2008. Depuis 1990, le taux de mortalité des moins de cinq ans dans les pays en développement a baissé de 28%, passant de 100 décès pour 1 000 naissances vivantes à 72 en 2008. En Afrique subsaharienne, même si la mortalité des moins de 5 ans a baissé de 22 % depuis 1990, les améliorations restent insuffisantes pour atteindre la cible. L'Afrique subsaharienne présente toujours les plus hauts taux de mortalité infantile. En 2008, un enfant sur 7 y mourait avant l'âge de 5 ans [7].

1.2. Malnutrition au Niger

Le Niger fait partie des pays de la région sahélienne en situation d'insécurité alimentaire récurrente [8]. Les récoltes ne se font qu'une fois par an et sont précédées d'une période de soudure s'étalant de mai/juin aux mois de septembre/octobre.

Le Niger a connu une crise alimentaire, pastorale et nutritionnelle aiguë en 2010, qui a affecté la moitié de la population totale. Cette crise a été marquée par une perte considérable d'animaux, un déficit céréalier et fourrager très important.

La campagne agropastorale de 2010-2011, même si débutée dans un contexte de crise généralisée, annonce un excédent prévisionnel en céréales locales et en fourrage de plus d'un million de tonnes. Ces résultats laissent envisager une situation alimentaire favorable pour l'année 2011. Néanmoins, des semences tardives et d'importantes inondations ont entrainé des déficits de production céréalière et fourragère dans toutes les régions du pays.

Selon les résultats de l'enquête nationale conjointe du Système d'Alerte Précoce (SAP) sur la vulnérabilité à l'insécurité alimentaire des ménages, 2,6 millions de personnes (soit 17,3% d'une population estimée à 15,7 millions [1]) sont en insécurité alimentaire en janvier 2011, environ la moitié étant touchée par l'insécurité alimentaire sévère.

Les régions présentant les taux d'insécurité alimentaire sévère en milieu rural les plus élevés sont Tahoua, Zinder et Maradi avec des taux d'insécurité alimentaire sévère variant ente 6,6 à 8,8 % de la population. Les départements les plus affectés par l'insécurité alimentaire sévère sont : Tahoua (23,6%), Abalak (14,0%), Tchintabaraden (14,0%), Tillabéri (13,2%), Madarounfa (12,9%), Mirriah (12,2%), Téra (11,2%), Guidan Roumji (10,8%), Madaoua (10,6%).

La flambée du prix du pétrole et des denrées alimentaires conjuguée à une série de soulèvements sociaux dans les pays arabes et une situation politique chaotique en Côte d'Ivoire constitue un facteur de risque pour l'accès des populations aux produits de première nécessité. Cette situation pourrait exposer les ménages les plus pauvres à l'insécurité alimentaire. Des effets importants des flux migratoires en provenance de la Libye, de la Côte d'Ivoire, de l'Egypte et du Nigeria sur la sécurité alimentaire pourraient se faire ressentir surtout dans les régions d'Agadez, de Tahoua, et de Zinder.

Le nombre d'enfants traités pour la malnutrition sévère au Niger chaque année depuis 2005 se mesure par centaine de milliers. Les diminutions de la disponibilité et de l'accès alimentaire ne font qu'aggraver une situation déjà critique pour les jeunes enfants nigériens. Les prévalences de la malnutrition globale (43% en 2008) et du retard de croissance des enfants de moins de 5 ans (55% en 2008) ne se sont pas améliorées dans les dernières années d'après les enquêtes démographiques et sanitaires nationales [9]. Le taux de mortalité estimé parmi les enfants de moins de 5 ans a diminué de façon significative entre 1990 (300/1000 naissances vivantes) et 2009 (160/1000 naissances vivantes), mettant en relief les efforts nationaux réalisés pour atteindre les OMD. Néanmoins, le Niger reste encore au 13ème rang des pays à plus fort taux de mortalité infanto-juvénile [9].

[1] Institut National de la Statistique - Niger, *le Niger en chiffres* (projection 2011).

De janvier à avril 2011, 59 395 cas de malnutrition sévère et 86 634 cas de malnutrition modérée d'enfants de moins de 5 ans ont été enregistrés au niveau national. Les régions les plus affectées sont Maradi, Zinder et Tahoua (source : Comité National de Gestion des Epidémies, avril 2011). A Maradi, entre janvier et avril 2011, on dénombre 29 390 cas de malnutrition aiguë sévère (contre 25 767 sur la même période 2010) et 30 938 cas de malnutrition aiguë modérée.

D'ici la fin 2011, les Nations Unies estiment que le nombre d'enfants nigériens de moins de 5 ans atteints de malnutrition aiguë sévère s'élèvera à 200 000 (contre plus de 340 000 cas enregistrés en 2010).

1.3. De la prise en charge curative à la prévention de la malnutrition

- **Les stratégies curatives**

Les suppléments nutritionnels à base de lipides (Lipid-based Nutrient Supplements - LNS) sont disponibles depuis le début des années 2000. Ils ont montré leur efficacité dans la croissance des enfants [10] et la prévention du retard de croissance [11,12].

Les **Aliments Thérapeutiques Prêts à l'Emploi** (ATPE) font partie de la gamme des LNS. Ils sont consommables sans préparation, faciles à avaler par l'enfant, faciles à stocker, et leur propriété anhydre limite la prolifération bactérienne. Utilisés en curatif chez les enfants de moins de 5 ans, ils ont prouvé leur efficacité dans le traitement de la malnutrition aiguë modérée et sévère, et la réduction de la probabilité de rechute ou de décès [13-17]. De plus, les ATPE utilisés dans le traitement de la malnutrition modérée ont montré de meilleurs résultats en terme de taux de guérison que les traitements standards à base de farines fortifiées [18,19]. Ainsi, unanimement reconnus par le Programme Alimentaire Mondial (PAM), l'UNICEF et les ONG, ils permettent aujourd'hui une prise en charge communautaire de la malnutrition aiguë sévère [13,20-22].

- **Les stratégies préventives**

La malnutrition précoce peut entrainer un retard du développement psychomoteur du jeune enfant. Ceci peut avoir des répercutions négatives sur la santé de l'enfant, sa scolarité puis ses capacités professionnelles augmentant sa vulnérabilité à la pauvreté. De plus en plus d'études tendent à montrer que les dommages physiologiques causés par la malnutrition précoce (entre la vie fœtale et l'âge de 24 mois) sont irréversibles.
Cette période pendant laquelle une nutrition correcte est primordiale, pendant la grossesse et avant l'âge de 2 ans, est donc la fenêtre critique d'opportunité ("window of opportunity") pour prévenir la malnutrition et offrir à l'enfant la meilleure chance de survie et optimiser sa croissance et son développement [1].

Bien que des progrès importants dans la prise en charge curative de la malnutrition aiguë aient été réalisés, la prévention de la malnutrition reste encore un défi. Des approches préventives de la malnutrition basées sur une supplémentation alimentaire ont récemment été évaluées dans différents pays en développement. Certaines études ont montré leur meilleure efficacité (ou équivalente) à réduire la malnutrition par rapport aux programmes thérapeutiques ciblant uniquement les enfants malnutris [23,24]. Si les stratégies de prévention visent à diminuer le nombre d'enfants affectés par la malnutrition sévère et modérée (et par conséquent la mortalité infantile qui en est liée), elles permettraient également de réduire le nombre d'enfants pris en charge dans les programmes thérapeutiques, exigeants en terme de ressources et de personnel.

L’apport en nutriments par les ATPE est très important par rapport aux besoins des enfants modérément malnutris et leur coût élevé ne permet pas une distribution à large échelle. Pourtant, ces produits utilisés en préventif ont montré un impact sur la diminution de l’incidence de la MAG et de la MAS [25-27].

De nouveaux produits de supplémentation de la gamme LNS mieux adaptés à la prévention de la malnutrition ont été mis au point, principalement les **Aliments de Supplémentation Prêts à l’Emploi** (ASPE) [15,19,28] . Pouvant être distribués en petite quantité, il est également supposé que les ASPE ont moins de risque d’être partagés au sein de la famille par rapport aux farines enrichies ce qui réduirait le besoin de rations de protection familiale (fréquemment distribuées dans les programmes de supplémentation).

Les distributions préventives à base de pâtes enrichies prêtes à l’emploi (ASPE) ont montré leur intérêt et leur efficacité dans divers pays en développement [10,17,19,28]. En effet, les ASPE semblent améliorer la croissance des enfants non malnutris et modérément malnutris [10,28].

Une diminution durable de l’incidence du retard de croissance sévère parmi des enfants supplémentés pendant 12 mois par un ASPE a été observée en comparaison à des enfants recevant une farine enrichie. Cet effet bénéfique des ASPE sur le gain de poids et taille est maintenu après la période de distribution [11,12].

La distribution préventive d’ASPE à 250 kcal/j à des enfants de 6-36 mois pendant la période de soudure a montré une réduction de l’incidence et la prévalence de la MAS [24].

Nécessitant un suivi à long terme sur un effectif important, peu d’études se sont intéressées à l’effet des distributions préventives sur la mortalité. Au Malawi, un suivi de 3 ans n’a pas montré de différences significatives des taux de mortalité entre les distributions d’ASPE et les farines enrichies [12].

Les résultats de ces études restent néanmoins difficiles à interpréter en raison des différents paramètres utilisés : les suppléments alimentaires utilisés comme aliments de comparaison (CSB ou autres), les types et les quantités de suppléments alimentaires délivrées, la période et la durée de distribution, la population cible. En outre, ces études n’ont pas mesuré les apports alimentaires complémentaires consommés par les bénéficiaires pendant l’intervention. Seules quelques études laissent penser que les ASPE sont bien acceptés [24], complétant sans remplacer probablement la consommation d’autres aliments et de lait maternel [10,29,30].

Les **farines enrichies** type « Corn-Soy Blend (CSB) », mélanges de soja et de farine de maïs enrichis en micronutriments [31], largement utilisées dans les programmes de supplémentation sont très peu adaptés aux besoins spécifiques des enfants les plus à risque (6 à 23 mois). On note en effet l’absence de lait, un faible apport énergétique, une quantité importante de facteurs antinutritionnels, et l’absence de certains micronutriments essentiels. Ces produits ont montré peu d’efficacité en matière de stimulation de la croissance des enfants [19,31-33]. De plus, ils s’avèrent peu appropriés au principe de distributions ciblées (rations difficiles à partager notamment). En janvier 2010, un nouveau mélange de micronutriments est incorporé au CSB qui prend alors le nom de CSB+, destiné aux enfants de plus de 2 ans [34].

Pour répondre spécifiquement aux besoins nutritionnels des enfants de 6 à 23 mois et les enfants souffrant de malnutrition modérée, une nouvelle génération de farines enrichies (CSB++) est développée début 2011. Les modifications apportées concernent notamment l'incorporation de lait en poudre, de sucre et d'huile dans la farine, la révision du mélange de fortification en minéraux et vitamines ainsi qu'un mode de raffinage du soja et du maïs permettant de mieux neutraliser les facteurs antinutritionnels que peuvent comporter ces aliments. Les rations sont aussi emballées dans des paquets individuels comportant l'équivalent d'une semaine de suppléments ce qui facilitent leur distribution (portions de 3 à 6 kg par mois (100 à 200g par jour) contre 25 kg pour le CSB). Contrairement aux ASPE, la consommation du CSB++ nécessite une préparation sous forme de bouillie (ajout d'eau propre et cuisson pendant 5 à 10 minutes).

Avant de généraliser l'utilisation de ce nouveau produit à la place des anciens mélanges, il est nécessaire d'obtenir plus d'informations quant à son efficacité sur la prévention de la malnutrition des enfants de moins de 2 ans.

- **Les transferts d'argent liquide**

Les transferts ciblés d'argent liquide constituent un autre axe d'intervention qui vise à améliorer la situation de populations vulnérables, principalement en réduisant la pauvreté chronique ou transitoire et en prévenant l'appauvrissement lié aux périodes de crises.

Le principe repose principalement sur le renforcement de la sécurité alimentaire des ménages les plus vulnérables, leur offrant la possibilité de consommer un régime alimentaire adéquat et équilibré, de maintenir leur hygiène, d'accéder aux services de santé et de faciliter leur investissement sur leurs propres moyens de production et le développement de leurs enfants.

Au-delà de l'aspect socio-économique, les programmes incluant le transfert d'argent jouent un rôle important sur l'amélioration de l'alimentation des ménages.

A Mexico, le transfert d'argent liquide et la distribution de ration de protection en vivres aux ménages vulnérables ont entrainé une augmentation de la consommation énergétique, une diversification alimentaire et un apport amélioré en micro et macronutriments en comparaison à un groupe sans intervention [35]. Les effets de la ration de protection en vivres étaient plus importants que le transfert d'argent.

Au Niger, un projet pilote de transfert d'argent liquide aux ménages vulnérables [36] a décrit un changement dans le profil de dépenses (augmentation des dépenses liées à la nourriture et l'eau) et une réduction au recours à des mécanismes d'adaptation nuisibles. L'alimentation, même si elle se diversifie et couvre les besoins énergétiques minimum, elle ne comble pas tous les besoins en micronutriments. Concernant le statut nutritionnel des enfants de moins de 5 ans, cette intervention n'a pas mis en évidence un impact significatif sur la réduction de l'incidence de la MAS et MAG à court terme. En 2010, dans la même zone du Niger, des distributions d'argent liquide ont complété des distributions de suppléments alimentaires pour les enfants de 6 à 23 mois sans montrer une amélioration de leur état de santé.

Pourtant, certains autres programmes de transfert d'argent en liquide aux ménages vulnérables auraient montré un impact sur la réduction de la prévalence de la malnutrition et du retard de croissance chez les enfants de 0 à 5 ans [37] (Mexico, Brésil, Nicaragua, Colombie [38], Brésil, Zambie). D'autres n'auraient pas pu mettre en évidence d'impact sur le statut nutritionnel des enfants (Honduras, Niger) [37]. Il est à noter que ces résultats ne sont basés que sur le monitoring de programme (avec des outils plus ou moins standardisés) et ne sont pas des études proprement dites. La causalité directe des distributions d'argent ne peut pas être clairement établie. De plus, les modalités de distribution d'argent varient beaucoup selon les projets, même au sein d'un même pays. Les différences principales concernent la durée de l'intervention, le montant de l'argent distribué adapté ou non au nombre de membres dans le ménage et les critères de sélection des ménages.

Ainsi, de façon générale, peu de données existent permettant d'établir un lien d'association entre la prévention de la malnutrition aiguë chez les jeunes enfants et le transfert d'argent liquide aux populations vulnérables. Des investigations complémentaires sont à mener.

En réponse à l'urgence de la crise alimentaire et nutritionnelle qui sévit au Niger en cette période de soudure, il paraît essentiel de mettre en place une évaluation et un suivi de différentes stratégies de prévention de la malnutrition. Le suivi du statut nutritionnel des enfants bénéficiaires permettra de décrire l'impact des différentes stratégies de prévention de la malnutrition, encore peu documentées à ce jour [34].

1.4. Description des interventions en 2011-2012

Les interventions du projet commun Forsani/MSF-OCP, dans le district de Madarounfa seront pour les années 2011-2012:

- Prise en charge de la MAS compliquée en hospitalisation : CRENI de Madarounfa ;
- Hospitalisation pédiatrique : hôpital de district de Madarounfa ;
- Prise en charge de la MAS en ambulatoire et du paludisme : CRENAS de Safo, Tofa, Gabi, Dan Issa, Madarounfa ;
- Prise en charge globale de la santé de l'enfant de moins de 5 ans : cases de santé et centre de santé intégré de 3 aires de santé (Safo, Gabi, Tofa) : consultations pédiatriques, prise en charge précoce des cas de paludisme, vaccination, malnutrition.
- Forsani/MSF-OCP adopte une stratégie de prévention de malnutrition en organisant une distribution d'ASPE (Plumpy'Doz®) à 8 600 enfants de 6 à 23 mois pendant 12 mois au niveau des cases de santé des aires de santé de Safo, Gabi, Tofa.

Les interventions du PAM au Niger seront pour l'année 2011 (avril – septembre) :

- Prévention de la MAM et MAS : Distribution ciblée pour un maximum de 500 000 enfants de 6-23 mois dans 164 zones vulnérables (CSB++ ou Plumpy'Doz®), de mai à septembre.
- Interventions de support de la sécurité alimentaires des ménages vulnérables dans le cadre du plan de soutien 2011 du Dispositif National de Prévention et Gestion des Crises Alimentaires (DNPGCA). Au sein de ces 164 zones vulnérables, le PAM avec ses propres ressources et le Dispositif avec d'autres ressources, mettront en œuvre des interventions ciblées sur les ménages vulnérables :
    - 122 zones avec accès à un marché alimentaire fonctionnel
        - Argent contre travail (avril – juin)
        - Argent ou vivres sans condition (distributions gratuites ciblées) pendant la saison agricole (juillet - septembre) pour les ménages en insécurité alimentaire sévère seulement
    - 42 zones sans accès à un marché alimentaire fonctionnel
        - Ration alimentaire contre travail (avril – juin)
        - Vivres sans condition (distributions gratuites ciblées) pendant la saison agricole (juillet - septembre) pour les ménages en insécurité alimentaire sévère seulement

2. Rationnel

La malnutrition aiguë est endémique au Niger. Elle se caractérise par un pic de l'incidence pendant la période de soudure (4-5 mois) plus ou moins important selon les années.

Parmi les enfants de 6 à 59 mois traditionnellement pris en charge dans les programmes nutritionnels au Niger, la tranche d'âge la plus à risque de malnutrition aiguë est celle des 6 à 23 mois, suivie des 24 à 35 mois.

En période de soudure, la distribution préventive de certains suppléments alimentaires type ASPE pour les enfants a montré un impact intéressant sur la réduction de l'incidence de la malnutrition sévère et globale ainsi qu'une réduction de l'affluence des enfants dans les programmes thérapeutiques. Nous nous interrogeons actuellement sur les possibilités d'optimisation de ces résultats et la place à donner à des interventions complémentaires de protection de la sécurité alimentaire au sein des ménages.

De plus, l'endémicité de la malnutrition aiguë au Niger interroge sur l'impact d'une stratégie de prévention de la malnutrition à plus long terme, basée sur des distributions de suppléments alimentaires tout au long de l'année. L'objectif visé serait une réduction de la mortalité des enfants mais également une diminution du retard de croissance par un apport continu de macro et micro nutriments adaptés au développement de l'enfant.

Plusieurs types de produits différents (en termes de valeur énergétique, de composition et de conditionnement – cf. annexe 1) sont disponibles actuellement pour prévenir la malnutrition. Différentes stratégies de distribution de ces produits ont déjà été mises en place ces dernières années au Niger, associées ou non à une protection de la sécurité alimentaire des ménages. Pourtant, peu d'informations sont actuellement disponibles pour les comparer en termes de prévention de la malnutrition modérée, de réduction de l'incidence de la malnutrition aiguë et de baisse de la mortalité chez les enfants de 6 à 23 mois.
Le transfert d'argent liquide aux ménages pendant la période de soudure associée ou non à la distribution de produits alimentaires nécessite également d'être documentée en particulier en terme d'impact sur la malnutrition des enfants.

Ainsi, il parait essentiel d'évaluer différentes stratégies de prévention de la malnutrition au sein d'un essai pragmatique afin de pouvoir comparer leur impact sur la santé des enfants de 6-23 mois dans une zone d'intervention au Niger.
Les résultats de cette étude permettront d'apporter des éléments objectifs aux partenaires /décideurs nationaux et internationaux pour orienter leurs choix d'interventions futures.

Les choix de ces différentes stratégies de distribution présupposent les hypothèses suivantes :

1. Les rations de suppléments nutritionnels (ASPE à 250 kcal/jour ou ASPE à 500 kcal/jour ou CSB++ à 800kcal/jour) distribuées en période de soudure protègent contre le développement de la MAG chez les enfants de 6 à 23 mois ;
2. La distribution d'ASPE avec un plus grand apport calorique (ASPE 500 kcal/jour versus ASPE 250 kcal/jour) pendant la période de soudure protège mieux les enfants contre le développement de la MAG.
3. Le support de la sécurité alimentaire des ménages (transfert d'argent liquide ou ration alimentaire de protection) associées aux rations de supplémentation pour les 6-23 mois protègent davantage contre la MAG que les rations de supplémentation seules ;
4. Une distribution prolongée de suppléments nutritionnels (ASPE à 250 kcal/jour ou ASPE à 500 kcal/jour ou CSB++ à 400-800kcal/jour) pendant et en dehors de la période de soudure diminuent la mortalité des enfants de 6 à 23 mois en les protégeant contre le développement de la MAG et prévient le retard de croissance ;
5. Une distribution préventive de suppléments alimentaires aux enfants de 6 à 23 mois (ASPE à 250 kcal/jour ou ASPE à 500 kcal/jour ou CSB++ à 400kcal/jour) plusieurs mois avant la période de soudure prévient l'augmentation de l'incidence de la MAG et la mortalité pendant la période de soudure ;
6. Le transfert d'argent liquide aux ménages, associé à une distribution de suppléments alimentaires aux les enfants de 6 à 23 mois pendant la période de soudure, a plus d'impact sur la prévention de la malnutrition de ces enfants que le transfert d'argent liquide seul.

3. Objectifs

3.1. Objectif principal

Mesurer l'incidence mensuelle et annuelle de la malnutrition aiguë globale et sévère des enfants âgés de 6 à 23 mois ciblés par différentes stratégies de prévention de la malnutrition de juin 2011 à novembre 2012 dans la région de Maradi, district sanitaire de Madarounfa :

A. ASPE à 500kcal/jour + transfert d'argent pendant 5 mois de soudure (juin – octobre 2011) puis ASPE à 500kcal/jour pendant 12 mois (novembre 2011 – octobre 2012) ;

B. CSB++ à 800 kcal/jour pendant 5 mois de soudure (juin – octobre 2011) puis CSB++ à 400kcal/jour pendant 7 mois (novembre 2011 – mai 2012) puis CSB++ à 800 kcal/jour pendant 5 mois de soudure (juin – octobre 2012) ;

C. ASPE à 500kcal/jour pendant 5 mois de soudure (juin – octobre 2011) puis ASPE à 250kcal/jour pendant 12 mois (novembre 2011 – octobre 2012) ;

D. ASPE à 250kcal/jour + transfert d'argent pendant 5 mois de soudure (juin – octobre 2011) puis ASPE à 250kcal/jour pendant 7 mois (novembre 2011 – mai 2012) puis ASPE à 500kcal/jour pendant 5 mois de soudure (juin – octobre 2012) ;

E. CSB++ à 800 kcal/jour + transfert d'argent pendant 5 mois de soudure (juin – octobre 2011) ;

F. CSB++ à 800 kcal/jour + ration de protection en vivres pendant 5 mois de soudure (juin – octobre 2011) ;

G. transfert d'argent pour les ménages comportant au moins un enfant de 6 à 23 mois pendant 5 mois de soudure (juin – octobre 2011).

3.2. Objectifs secondaires

1. Evaluer le nombre d'enfants de 6 à 23 mois admis au sein de programme nutritionnel (hospitalisation et/ou référence en CREN), parmi les villages sélectionnés ;
2. Estimer la mortalité des enfants de 6 à 23 mois dans les villages sélectionnés ;
3. Estimer l'importance du retard de croissance des enfants de 6 à 23 mois dans les villages sélectionnées ;
4. Décrire les pratiques alimentaires des enfants de 6 à 23 mois dans les villages sélectionnés.

4. Méthodes

4.1. Type d'étude

Il s'agit d'une étude interventionnelle prospective qui consiste en des distributions alimentaires et/ou d'argent liquide aux enfants âgés de 6 à 23 mois habitant les villages sélectionnés, accompagnées du suivi longitudinal de leur statut nutritionnel. Ces distributions et suivis seront mensuels pendant une période de 18 mois de juin 2011 à novembre 2012.

Cette étude s'appuie sur les principes d'un essai pragmatique. En effet, pour tenir compte des contraintes logistiques et des critères de faisabilité, le tirage au sort ne se fera pas sur l'individu. Le village reste la plus petite unité prise en compte dans les stratégies de distribution à large échelle type « blanket feeding ».

La stratégie utilisée pour l'intervention (distributions de vivres ou d'argent) sera la plus proche possible de celle classiquement utilisée par Forsani/MSF-OCP ou d'autres partenaires nationaux du Programme Alimentaire Mondial (PAM).

Les données recueillies sur les enfants (périmètre brachial, poids, taille) sont celles habituellement prises en compte dans les programmes nutritionnels au niveau national et international pour suivre le statut nutritionnel d'un enfant et correspondent aux critères de l'OMS publiés en 2005.

Le projet implique des acteurs intervenant habituellement dans la zone (agents sanitaires du MSP, ONG internationales et locales, PAM).

La mise en place de l'étude dans des conditions normales d'intervention permettra une meilleure applicabilité des résultats pour les interventions futures de nos partenaires.

4.2. Groupes d'intervention

Sept groupes d'enfants seront constitués (cf. annexe 2).

Le bras A de la cohorte est désigné comme le contrôle positif, car son intervention est considérée comme optimale (ASPE hautement énergétique pour l'enfant associé à une aide financière pour le ménage). Nous supposons que ce bras présentera les meilleurs résultats en termes de diminution de l'incidence de la MAS et de prévention de la MAM.

- Bras A. ASPE à 500kcal/jour + transfert d'argent pendant 5 mois de soudure (juin – octobre 2011) puis ASPE à 500kcal/jour pendant 12 mois (novembre 2011 – octobre 2012) ;
- Bras B. CSB++ à 800 kcal/jour pendant 5 mois de soudure (juin – octobre 2011) puis CSB++ à 400kcal/jour pendant 7 mois (novembre 2011 – mai 2012) puis CSB++ à 800 kcal/jour pendant 5 mois de soudure (juin – octobre 2012) ;
- Bras C. ASPE à 500kcal/jour pendant 5 mois de soudure (juin – octobre 2011) puis ASPE à 250kcal/jour pendant 12 mois (novembre 2011 – octobre 2012) ;
- Bras D. ASPE à 250kcal/jour + transfert d'argent pendant 5 mois de soudure (juin – octobre 2011) puis ASPE à 250kcal/jour pendant 7 mois (novembre 2011 – mai 2012) puis ASPE à 500kcal/jour pendant 5 mois de soudure (juin – octobre 2012) ;
- Bras E. CSB++ à 800 kcal/jour + transfert d'argent pendant 5 mois de soudure (juin – octobre 2011) ;
- Bras F. CSB++ à 800 kcal/jour + ration de protection en vivres pendant 5 mois de soudure (juin – octobre 2011) ;
- Bras G. transfert d'argent pour les ménages comportant au moins un enfant de 6 à 23 mois pendant 5 mois de soudure (juin – octobre 2011).

Les ASPE et CSB++ seront distribués par Epicentre mensuellement avec le même mode opératoire pour chacun des bras d'intervention.

Les ménages concernés par le transfert d'argent ou la distribution de ration alimentaire (bras A, D, E, F et G) seront tous les ménages hébergeant au moins un enfant de 6 à 23 mois inclus dans l'étude. Les transferts d'argent seront organisés en collaboration avec une institution de micro-finance (ASUSU) définie par le PAM.

Dans les bras A, D et E, chaque ménage recevra 25 000 FCFA (environ 38 euros) par mois pendant 5 mois (soit un total 125 000 FCFA, environ 190 euros). Ce montant est défini pour permettre d'acheter les biens alimentaires de base (céréales, légumineuses, huile) pouvant couvrir les besoins énergétiques d'un ménage de 7 personnes (sur la base d'un apport quotidien recommandé de 2100 kcal).

Dans le bras G, chaque ménage recevra 28 000 FCFA (environ 43 euros) par mois pendant 5 mois (soit un total 140 000 FCFA, environ 213 euros). Les 3000 FCFA supplémentaires visent à couvrir les besoins alimentaires de l'enfant de moins de 2 ans, ne recevant pas de suppléments alimentaires.

Dans le bras F, la ration de protection en vivres sera distribuée par Epicentre pendant 5 mois. Chaque ménage recevra le nombre de rations alimentaires égal au nombre d'enfants de ce ménage inclus dans l'étude. Elle sera composée de 50 kg de céréales, 7,5 kg de légumineuses et 2,5 kg d'huile.

Les groupes d'intervention bénéficiant d'une intervention pendant seulement 5 mois (bras E, F et G) seront suivis à 6 mois, 13 mois (avant le début de la période de soudure) et à 18 mois (après le début de soudure).

Chaque bras d'intervention recevra les mêmes messages de sensibilisation et conseils sur la croissance et l'alimentation des enfants. Seulement une partie spécifique concernant l'utilisation du produit distribué sera adaptée selon le groupe d'intervention.
Les interventions communes dont bénéficieront tous les enfants inclus dans l'étude :

- dépistage actif des cas de malnutrition aiguë sévère ;
- référence au CRENAS le plus proche en cas de malnutrition sévère ;
- accès à des consultations pédiatriques par un agent de santé au niveau d'une case de santé ou d'un centre de santé intégré soutenu par le projet commun Forsani/MSF-OCP ;
- sensibilisation et conseils sur la croissance et l'alimentation des enfants.

4.3. Zone de l'étude

L'étude sera menée dans le district sanitaire de Madarounfa, dans le sud de la région de Maradi (cf. annexe 3). Cette zone compte environ 405 000 habitants. Elle a été choisie comme zone d'intervention de l'étude pour plusieurs raisons. Tout d'abord, elle a été déclarée comme à risque d'insécurité alimentaire sévère par l'enquête du Système d'Alerte Précoce de janvier 2011 et plusieurs villages y ont été ciblés par les interventions du PAM. De plus, 5 aires de santé de ce district sont soutenues par le projet commun Forsani/MSF-OCP. Enfin, l'épidémiologie du paludisme est supposée comparable sur toute la zone très restreinte. Notre base de recherche Epicentre, située à Maradi, permettra de faciliter les interventions (cf. annexe 9).

4.4. Population cible

Seront recrutés tous les enfants âgés de 6 à 23 mois, habitant dans les villages sélectionnés.

Ils seront inclus la veille de la première distribution, et seront suivis mensuellement pendant toute la durée de l'étude.

La cohorte sera incrémentée au fur et à mesure de l'éligibilité des enfants nés récemment et atteignant l'âge de 6 mois au moment des distributions mensuelles. Les sorties de cohorte s'effectueront au fur et à mesure que les enfants inclus atteindront l'âge de 24 mois.

Pour les bras E, F et G bénéficiant d'une intervention pendant 5 mois, les suivis ultérieurs (à 6, 13 et 18 mois) cibleront les enfants ayant entre 6 et 23 mois au moment de la visite.

Les ménages ciblés par la distribution de ration de protection ou de transfert d'argent liquide seront les ménages auxquels sont rattachés les enfants inclus dans la cohorte.

4.5. Critères d’inclusion des enfants dans la cohorte

Dans chaque village sélectionné, les critères d’inclusion des enfants seront :

- une taille couchée supérieure à 60 cm et inférieure ou égale à 80 cm
- résidant dans le village sélectionné (résidence principale).

Le critère de taille est celui habituellement pris en compte pour identifier les enfants de 6 à 23 mois, bénéficiaires de distributions type « blanket feeding » au Niger.

Pour chacun des enfants sélectionnés, les consentements du chef de famille, de la mère ou du tuteur de l’enfant seront demandés. Un temps sera consacré avec chacun d’eux afin d’expliquer les objectifs et le déroulement de l’étude.

Le refus de participer à l’étude n’aura pas de conséquence sur le fait de recevoir ou non les interventions communes (dépistage actif de la MAS, référence en CRENAS si nécessaire, accès aux soins et sensibilisation).

4.6. Critères d’exclusion des enfants dans la cohorte

Les enfants sélectionnés ne seront pas inclus dans l’étude s’ils présentent un des critères suivants :

- Enfant dont le village sélectionné n’est pas la résidence principale ;
- Enfant dont l’allergie à l’arachide ou à n’importe quel autre produit ayant nécessité une prise en charge médicale en urgence, est connue ;
- Enfant souffrant d’une pathologie rendant la déglutition difficile ;
- Enfants dont le représentant légal refuse de participer à l’étude.

Les critères suivants ne sont pas des critères d’exclusion de l’étude. Dans les cas suivants, l’enfant ne reçoit plus les produits prévus lors de la distribution mais le suivi est maintenu. Il pourra à nouveau recevoir les produits une fois qu’il ne présentera plus ces critères :

- Enfant pris en charge dans un programma nutritionnel (CRENAS ou CRENI) ;
- Enfant dépisté malnutri sévère le jour de la distribution et référé en CRENAS.

4.7. Echantillonnage : nombre de sujets

Nous considérons le bras d'intervention A (ASPE à 500kcal/jour + transfert d'argent pendant 5 mois de soudure puis ASPE à 500kcal/jour pendant 12 mois) comme la cohorte de contrôle positif bénéficiant de la meilleure stratégie possible dans une optique de réduction de l'incidence de malnutrition. Nous considérons aussi le bras d'intervention G (transfert d'argent pour tous les ménages comportant au moins un enfant de 6 à 23 mois) comme étant la stratégie a priori la moins efficace.

Nous faisons l'hypothèse qu'après 6 mois de distributions ciblées, les enfants suivis dans le bras d'intervention G auront des rapports poids/taille en moyenne inférieurs de 20% aux enfants du bras d'intervention A (basés sur les résultats d'autres études Epicentre au Niger). En considérant un risque de première espèce de 5%, une puissance de 80%, un écart-type du rapport poids-taille de 1 dans chaque bras, une corrélation entre chaque mesure de 0,5 et un nombre de mesures par enfant au total égal à 12 en incluant l'inclusion, nous devons inclure 471 enfants dans chaque stratégie pour espérer mettre en évidence une telle différence. En admettant une proportion d'enfants absents ou perdus de vue égale à 10% de l'échantillon à chaque visite, nous devrons donc admettre au minimum 500 enfants par bras.

4.8. Critères de sélection des villages

Au sein du district sanitaire de Madarounfa, environ 50 villages répondant aux critères suivants seront inclus dans l'étude :

- villages appartenant aux aires de santé de TOFA, DAN ISSA, MADAROUNFA ou ANGOUALMATA. Ces aires de santé sont soutenues par le projet commun Forsani/MSF-OCP ou se trouvent à moins de 10 km d'un CRENAS soutenu par le projet commun Forsani/MSF-OCP;
- villages hors zone urbaine ;
- villages et hameaux présents sur la liste officielle du district sanitaire de Madarounfa ;
- villages situés à moins de 15 km du centre de santé intégré de rattachement ;
- villages dans lesquels aucune autre intervention de prévention de la malnutrition n'est prévue avant le début de l'étude ;
- villages et hameaux formant un groupement de 500 à 800 enfants de 6 à 23 mois.

Pour chacun des villages sélectionnés, le consentement signé du chef de village sera demandé. Un temps sera consacré avec les organisations communautaires du village (groupement de femmes par exemple), ainsi que les autorités administratives, traditionnelles et cultuelles afin d'expliquer les objectifs et le déroulement de l'étude.

Un recueil de données sur les caractéristiques démographiques et socio-économiques des villages sélectionnés sera effectué en début d'étude (cf. annexe 5). Tout évènement important affectant le village et se produisant pendant l'étude sera enregistré (inondation, épidémie, invasion d'insectes, etc.). Cela permettra de documenter les différences importantes entre les villages comparés.

4.9. Attribution du groupe d'intervention

L'attribution des groupes d'intervention se fera de façon aléatoire par groupement de villages d'environ 500 enfants de 6 à 23 mois (cf. annexe 4).

Néanmoins, nous prendrons en compte des considérations opérationnelles et éthiques pour placer notre étude dans une approche pragmatique. Nous intégrerons en effet deux contraintes dans notre méthodologie d'attribution des bras d'intervention :

- Nous distribuerons le même produit que celui prévu lors d'interventions concomitantes par d'autres partenaires sur une même aire de santé (distribution de Plumpy'Doz® sur Tofa par Forsani/MSF-OCP) ;
- Les bras d'intervention recevant une distribution sur 5 mois seulement seront distants (aires de santé différentes dans la mesure du possible) des groupements de villages recevant une distribution sur 17 mois.

Pour faciliter la comparaison des différentes stratégies d'intervention en terme d'impact sur la malnutrition, il est important de limiter les disparités entre les bras d'intervention au début de l'étude. Ainsi, la composition des groupes d'intervention ne sera définitivement validée qu'après avoir vérifié que les moyennes des Z-score ne diffèrent pas de plus de 10% entre les bras.

4.10. Critères de jugement

Critère principal

Le critère principal mesuré est le taux d'incidence mensuelle et annuelle de la malnutrition aiguë sévère et globale, chez les enfants entre 6 et 23 mois dans les villages sélectionnés ayant reçu une intervention préventive de la malnutrition.

Critères secondaires

- Taux de prévalence de la malnutrition aiguë sévère et globale chez les enfants entre 6 et 23 mois dans les villages sélectionnés ;
- Taux de décès des enfants inclus ;
- Taux d'admission dans les CREN des enfants inclus (CRENAS et CRENI) ;
- Taux de rechute et de guérison des enfants inclus et admis en CREN ;
- Retard de croissance après 18 mois de suivi ;
- Diversité alimentaire minimale : Proportion d'enfants recevant une alimentation venant d'au moins 4 groupes d'aliments différents [2] dans le jour précédent ; [3]
- Fréquence de repas minimale : Proportion d'enfants recevant de la nourriture solide ou semi-solide au moins 2 à 4 fois selon l'âge dans le jour précédent ; [2]
- Régime acceptable minimal : Proportion d'enfants ayant une diversité alimentaire minimale et une fréquence de repas minimale. [2]

---

[2] Groupes d'aliments : (1) Céréales, racines et tubercules ; (2) légumes et noix ; (3) produits laitiers ; (4) viande, poisson, volaille ; (5) œufs ; (6) légumes et fruits riches en vitamine A ; (7) autres fruits et légumes.

[3] World Health Organization. Indicators for assessing infant and young child feeding practices part 2: measurement. 2010.

Le taux de mortalité dans chacun des bras sera suivi mensuellement. Si un doublement du taux de mortalité est observé entre deux suivis, l'intervention sera immédiatement stoppée dans le bras concerné. Elle sera remplacée par celle prévue dans le bras contrôle positif (bras A).

Une première analyse intermédiaire des données sera réalisée après 5 mois d'intervention, c'est-à-dire à la fin de la période de soudure (données de juin à octobre 2011). Une seconde analyse intermédiaire sera réalisée en mai 2012 (données de novembre 2011 à mai 2012), c'est-à-dire avant la période de soudure 2012. Enfin, une analyse générale sera effectuée sur l'ensemble des 18 mois de l'étude (données de juin 2011 à novembre 2012).

4.11. Définitions

Dans l'étude nous utiliserons les définitions suivantes :

- **Ménage**

Dans le cadre des distributions de rations de protection en vivres et en argent liquide, nous considérerons le ménage comme étant l'ensemble des membres ;

- qui mettent en commun tout ou une partie de leurs ressources ;
- qui mangent le repas préparé sur un même feu ;
- qui reconnaissent l'autorité d'une même personne appelée chef de ménage ;
- qui vivent ensemble dans le même logement.

- **Malnutrition aiguë**

Malnutrition Aiguë Globale*	<-2 z-score (poids sur taille)<br>et/ou œdèmes bilatéraux<br>et/ou PB<125 mm
Malnutrition Aiguë Modérée*	<-2 z-score (poids sur taille)<br>et ≥-3 z-score du poids sur la taille
Malnutrition Aiguë Sévère*	<-3 z-score (poids sur taille)<br>et/ou œdèmes bilatéraux<br>et/ou PB<115 mm

* Normes OMS 2005

- **Critères de référence vers un programme nutritionnel prenant en charge les cas de MAS**

- Enfants de 65 cm à 80 cm :
    - o <-3 z-score
    - o et/ou œdèmes bilatéraux
    - o et/ou PB<115 mm

- Enfants > 60 cm et < 65 cm
    - o <-3 z-score
    - o et/ou œdèmes bilatéraux

- **Critère de sortie guéri de programme nutritionnel**

L'ensemble des critères devront être réunis :

- o >-3 z-score
- o Absence d'œdèmes depuis 7 jours minimum
- o Absence de pathologie associée ou de traitement antibiotique en cours (sauf anti-TB)
- o Vaccination rougeole à jour

- **Perdu de vue**

« Perdu de vue » est défini comme un enfant absent lors de 2 distributions consécutives et confirmé par une visite à son domicile. Une recherche systématique de tous les perdus de vue est prévue.

- **Abandon**

« Abandon » est défini comme un enfant dont le représentant légal a volontairement souhaité le retirer de l'étude.

- **Inconnu**

« Inconnu » est défini comme un enfant absent lors de 2 distributions consécutives, et non confirmé comme « abandon » ou « décédé », par une visite à son domicile.

5. Déroulement de l’étude

5.1. Phase préparatoire

- Constituer un document de procédures de l’étude pour les équipes ;
- Préparer les questionnaires et formulaires de consentement ;
- Concevoir un carnet de suivi individuel des enfants remis au représentant légal (renseignements concernant la participation aux différentes distributions et l’admission éventuelle en centre nutritionnel) ;
- Concevoir une fiche de référence vers les centres nutritionnels (renseignements concernant les enfants qui seront admis dans les programmes), intégré au carnet de suivi individuel ;
- Etablir la liste des villages éligibles ;
- Recruter et former les équipes d’enquête (en charge du recueil des données) ;
- Recruter et former les équipes de distribution (en charge de la sensibilisation des ménages, de l’organisation des distributions et de la dispensation des rations alimentaires) ;
- Former les différents acteurs et faire un rappel théorique et pratique des mesures anthropométriques et méthodes de mesures (même méthode quel que soit le groupe) ;
- Etablir un calendrier d’événements locaux pour estimer l’âge des enfants en mois ;
- Effectuer un pré-test des questionnaires sur le terrain ;
- Effectuer des visites dans les villages sélectionnés pour rencontrer les chefs de villages et autres personnes clés pour présenter l’étude et demander leur consentement ;
- Identifier, dans chaque village sélectionné, des aides communautaires (qui seront présents au moment des visites et distributions et pourront aider à l’organisation des distributions, à l’identification des ménages et à l’introduction des équipes au sein de ces ménages).

5.2. Inclusion des villages

Les villages sélectionnés feront l’objet d’une visite préalable ; chaque chef de village (accompagné du chef religieux, d’une représentante de la communauté des femmes et autres organisations locales) sera informé du passage des équipes d’évaluation et du but de ce projet. Un consentement écrit du chef de village sera effectué avant toute inclusion.

Un recueil de données des caractéristiques du village sera effectué (cf annexe 5). Les données recueillies seront :

- Caractéristiques générales : nombre d’habitants, nombre des ménages, mobilité des habitants ;
- Caractéristiques socio-économiques : accès et source d’eau potable, accès aux structures sanitaires, marchés alimentaires, axes routiers principaux.

5.3. Sélection des enfants

Pour les villages inclus, la participation à l'étude sera proposée à tous les ménages, comprenant au moins un enfant dont la taille couchée est supérieure à 60 cm et inférieure ou égale à 80 cm, considéré comme âgé entre 6 et 23 mois. Avant toute inclusion, un temps d'explication sur les objectifs et le déroulement de l'étude sera pris avec le chef de ménage ou, en son absence, avec le représentant légal de l'enfant.

Ensuite, le recueil du consentement du représentant légal de l'enfant sera effectué. Il s'agira du père s'il est présent, ou de la mère ou encore de l'adulte en charge de l'enfant. Une lettre d'information lui sera lue en français ou en Haoussa. Il lui sera ensuite demandé de donner son consentement écrit en signant (ou empreinte digitale) le formulaire de consentement éclairé (Cf. Annexes 6 et 7).

Le fait qu'un ménage refuse de participer à l'étude ne modifiera pas l'accès aux soins de l'enfant. Il lui sera proposé d'évaluer le statut nutritionnel de l'enfant et de le référer au CRENAS si nécessaire.

5.4. Inclusion des enfants

Environ 4500 enfants seront recrutés dans la cohorte.
Tous les enfants devenant éligibles au cours de l'étude seront inclus au fur et à mesure.

- Date de première inclusion : juin 2011 (quelques jours avant la 1ère distribution) ;
- Tout enfant mesurant entre 60 et 80cm, présent et résidant dans le village sélectionné ;
- Enregistrement par un numéro d'identification unique ;
- Remise d'un carnet de suivi individuel ;
- Collecte des données sur l'enfant et le ménage (questionnaire ménage et enfant);
- En cas de dépistage de cas de MAS l'enfant sera référé vers le CRENAS le plus proche ;
- En cas de suspicion de pathologie sévère, une sensibilisation de l'accompagnant sera effectuée concernant l'accès gratuit aux soins dans les structures sanitaires du district.

5.5. Distributions préventives

Les distributions d'ASPE, CSB++ et rations de protection en vivres des ménages seront effectuées pendant toute la durée de l'étude par des équipes d'Epicentre en collaboration avec la communauté.

Elles seront organisées au sein même des villages ou groupes de villages inclus dans l'étude.

Elles seront précédées par des séances de sensibilisation de la population en particulier concernant la population cible et le mode d'utilisation des aliments distribués. Ces activités seront menées avec la participation de représentant de la communauté.

Les rations ne seront dispensées qu'en présence de l'enfant inclus et sur présentation du carnet de suivi de l'enfant.

Les types de produit et les quantités distribués sont décrits dans le tableau 1.

Tableau 1. Description du conditionnement et des quantités journalière et mensuelle pour Plumpy'Doz®, Supplementary'Plumpy® et CSB++.

	**Plumpy'Doz®**	**Supplementary' Plumpy®**	**CSB++**
**Conditionnement**	Pot de 325g	Sachet individuel 92 g	Sac de 1,5 kg
**Quantité journalière de produit par enfant**	46g<br>= 3 cuillères à café	92g<br>= 1 sachet	200g ou 100g
**Valeur énergétique journalière**	247 kcal	500 kcal	820 kcal ou 410 kcal
**Quantité mensuelle distribuée**	4 pots	30 sachets	4 ou 2 sacs

La fréquence des distributions sera mensuelle pendant toute la durée de l'étude (excepté pour les bras d'intervention E, F et G pendant seulement 5 mois).

Les enfants qui entrent dans un programme nutritionnel au cours de l'étude (le jour des distributions ou entre deux distributions) ne reçoivent pas la dose préventive. Ils la recevront à nouveau lorsqu'ils sortiront du programme. L'admission dans un programme nutritionnel et sa sortie seront enregistrées dans le carnet de suivi individuel de l'enfant.

5.6. Fréquence de suivi des enfants

Le suivi des enfants se fera tous les mois au moment de chaque distribution préventive ou distribution d'argent.

En cas de non présentation d'un enfant au point de distribution, une équipe se rendra à son domicile le lendemain de la distribution. S'il est présent, elle vérifiera l'état de santé de l'enfant et prendra ses mesures anthropométriques. Il ne bénéficiera pas de distribution préventive. Si l'enfant n'est pas présent, les raisons de l'absence seront recherchées auprès de l'entourage.

Pour les groupes bénéficiant d'une intervention pendant seulement 5 mois (bras E, F et G), les suivis seront mensuels pendant les 5 premiers mois, puis se dérouleront au 6ème mois, 13ème mois (juin 2012 – début de la période de soudure) et 18ème mois (novembre 2012 – fin de la période de soudure). Ces suivis se feront au domicile de chaque enfant. Les mêmes questionnaires enfant et ménage seront utilisés.

5.7. Données collectées

Les données suivantes seront collectées à l'aide d'un questionnaire pour chaque enfant inclus et chaque mois :

- Caractéristiques générales de l'enfant :
  - Nom et prénom des deux parents ou représentant légal de l'enfant
  - Age[4] ;
  - Sexe ;
  - Village d'origine ;
  - Date de l'inclusion ;
  - Numéro d'identification unique.
- Evènements médicaux :
  - Inclus dans un programme nutritionnel (CRENAS ou CRENI)
  - Hospitalisation ou consultation médicale hors CRENI dans le mois précédent
  - Décès et cause
- Mesures anthropométriques
  - Poids
  - Taille
  - Périmètre brachial
  - Présence d'œdèmes bilatéraux

[4] Si la date de naissance n'est pas connue ou prouvée par un document officiel par la mère ou le représentant légal, l'âge de l'enfant sera estimé à l'aide d'un calendrier évènementiel local.

- Données concernant les actions de prévention de malnutrition :
  - Bénéficiaire ou non de la précédente distribution de suppléments alimentaires ;
  - Utilisation de la ration ;
- Raisons expliquant l’absence de l’enfant lors du suivi (hormis décès et hospitalisation) :
  - Voyage, déménagement, migration, déplacement de la famille pour raisons professionnelles
  - Tricherie (enfant non résidant dans un village sélectionné et exclu du suivi a posteriori)
  - Abandon, perdu de vue
- Alimentation de l’enfant
  - Allaitement maternel
  - Fréquence des repas solides ou semi-solides
  - Diversité de l’alimentation

Les données suivantes concernant le ménage seront recueillies à l’inclusion, puis tous les 2 mois jusqu’à la fin de la période de soudure (octobre 2011), à l’aide d’un questionnaire :

- Caractéristiques démographiques du ménage
- Alimentation du ménage
- Sources de revenu du ménage
- Vulnérabilité alimentaire du ménage
- Utilisation de la ration de protection en vivres ou argent

Les questionnaires seront rédigés en français et traduits en Haoussa pendant l’entretien. Ils seront testés avant le démarrage de l’étude pour vérifier leur pertinence et leur acceptabilité auprès de la population. Des modifications pourront y être apportées avant d’éditer une version finale.

6. Traitement des données

6.1. Saisie des données

Les données seront saisies par des opérateurs de saisie employés par Epicentre et préalablement formés sur le logiciel EpiData 3.1 (EpiData Association, Odense, Denmark). La réalisation du masque de saisie, la supervision de la saisie, ainsi que le nettoyage de la base de données seront réalisés par un gestionnaire de données secondé par un contrôleur de données employés par Epicentre.

La saisie des données se déroulera en continu tout au long de l'étude. Le contrôle de qualité sera réalisé par le gestionnaire de données sur 10% des questionnaires.

6.2. Analyse des données

Les bases de données seront analysées avec le logiciel STATA 10 (StataCorp, College Station, TX, USA) par Epicentre.

6.3. Plan d'analyse

L'analyse prendra en compte la durée d'exposition des enfants à l'intervention.

Elle consistera principalement en :

- Description des caractéristiques principales de la population de chaque bras à l'inclusion et à chaque visite ;
- Evaluation de l'association entre chaque stratégie et les incidences de MAG et MAS ainsi qu'avec la mortalité en modélisant chacun des critères par un modèle de Cox et en contrôlant les éventuels facteurs confondants au moyen de scores de propension ;
- Evaluation de l'association entre chaque stratégie et les premiers épisodes de retard de croissance en utilisant un modèle de Cox et en contrôlant les éventuels facteurs confondants au moyen de scores de propension ;
- Evolution du statut nutritionnel des enfants selon les groupes d'intervention : caractérisation par stratégie du nombre d'épisodes de MAS ou MAM par enfant pendant la durée de l'étude, caractérisation du nombre d'enfants originellement malnutris et dont la situation nutritionnelle s'est améliorée ;
- Description de la diversité alimentaire des enfants de la cohorte selon les groupes d'intervention : taux d'enfants ayant un régime acceptable minimal (cf. 4.10).
- Description du devenir des enfants de la cohorte : taux d'hospitalisation et d'inclusion dans un programme nutritionnel
- Analyse de survie : comparaison des taux de mortalité par stratégie avec la méthode de Kaplan-Meier

7. Limites attendues

Cette étude, comme toutes les études longitudinales similaires a des limitations qu'il convient de souligner. Les résultats devront être extrapolés avec précaution. Ils seront uniquement représentatifs des villages présentant les mêmes caractéristiques que les villages inclus dans le district de Madarounfa. Les résultats seront également liés aux participants, aux intervenants, au mode de distribution ainsi qu'aux activités complémentaires telles que la recherche active des perdus de vue ou la prise en charge des cas de MAS dans les structures de santé soutenues par Forsani/MSF-OCP. De plus, ils seront spécifiques de l'environnement sanitaire des années étudiées.

Le nombre d'indicateurs recueillis est limité pour des raisons éthiques et opérationnelles. Certaines informations pouvant avoir un impact sur l'incidence de la malnutrition, ne seront pas recueillies.

Des différences non quantifiables et non contrôlables entre les villages, les familles et les enfants pourraient exister et limiter la description de l'impact des interventions. Tout évènement important relatif aux villages, aux ménages ou aux enfants sera donc enregistré pour tenter de documenter ces différences.

De même, la mobilité de la population pourrait entrainer des difficultés de suivi pendant toute la durée de l'étude. Néanmoins, la taille de l'échantillon est suffisante et prend en compte une certaine proportion de perdus de vue.

Les autres biais principaux attendus pourraient être :

- les biais sur les mesures du PB, du poids et de la taille ; un système de contrôle de qualité de la prise des mesures sera mis en place régulièrement pour limiter les imprécisions.
- les biais sur l'âge des enfants ; l'utilisation du calendrier des évènements locaux permettra de limiter les biais ;

Enfin, d'autres interventions extérieures concomitantes dispensées aux enfants pendant la période de l'étude pourraient limiter la comparaison des stratégies préventives. Une bonne collaboration avec les partenaires locaux devra permettre d'échanger des informations importantes sur leurs projets et l'intervention Epicentre. En collaboration avec le Ministère de la Santé et dans le respect des considérations éthiques, nous tenterons de préserver les interventions initiales d'Epicentre dans la zone. Dans le cas contraire, nous documenterons les interventions concomitantes.

8. Considérations éthiques

8.1. Autorisations

Le protocole sera soumis pour accord au Comité Consultatif National d'Ethique du Niger, au Ministère de la Santé Publique du Niger et au Comité de Protection des Personnes (CPP) Ile de France XI. Les autorités sanitaires, administratives et traditionnelles de la région de Maradi et du district de Madarounfa seront également tenues informées.

L'étude sera conduite en accord avec les principes de la Déclaration d'Helsinki (cf. annexe 8) et les directives éthiques internationales pour la recherche biomédicale impliquant des sujets humains, éditée par le Conseil des Organisations Internationales des Sciences Médicales (CIOMS).

8.2. Consentement éclairé

Les inclusions dans l'étude seront faites de façon volontaire, après consentement éclairé. La procédure de consentement inclura un formulaire de consentement éclairé (Cf. annexes 6 et 7), traduit en Haoussa.

Une lettre d'information sera lue entièrement au chef de village et autres représentants du village en français ou en Haoussa. Après avoir répondu aux éventuelles questions sur l'étude, l'enquêteur demandera son accord au chef pour inclure son village dans l'étude. Si c'est le cas, il devra signer le formulaire de consentement éclairé (ou apposer une empreinte digitale si la signature ne peut être obtenue) en deux exemplaires. Le chef de village en conservera un exemplaire, le second sera archivé à Epicentre à Maradi.

Au niveau du ménage, une lettre d'information sera lue entièrement au parent ou au responsable légal de l'enfant en français ou en Haoussa. Après avoir répondu aux éventuelles questions sur l'étude, l'enquêteur demandera au parent ou au responsable légal de l'enfant s'il accepte la participation de son enfant à l'étude. Si c'est le cas, il devra signer le formulaire de consentement éclairé (ou d'apposer une empreinte digitale si la signature ne peut être obtenue) en deux exemplaires. Le parent en conservera un exemplaire, le second sera archivé à Epicentre à Maradi.

Avant chaque suivi de l'enfant, un consentement oral sera demandé au parent ou responsable légal de l'enfant. Chaque ménage sera libre de refuser de participer à l'étude à tout moment ou ne pas répondre à certaines questions du questionnaire. Si un ménage refuse de participer à l'étude, il lui sera tout de même proposé un dépistage actif de la MAS parmi les enfants du foyer, une référence en CRENAS si nécessaire, ainsi qu'un accès aux soins gratuits pour les enfants de moins de 5 ans.

8.3. Confidentialité

Les données concernant les enfants et les ménages inclus seront confidentielles et ne seront communiquées à personne en dehors de l'équipe de l'étude, sauf si cela est jugé nécessaire pour protéger la santé d'un patient. Ni les questionnaires, ni la base de données électronique ne contiendront de données personnelles concernant les participants.

La confidentialité sera garantie par un système de codage adapté. Les données nominales ne seront pas diffusées hors du site d'étude. Les dossiers des patients et toute information personnelle concernant les sujets participant à l'étude seront strictement réservés au personnel soignant et aux investigateurs directement impliqués dans l'étude.

8.4. Archivage

Tous les questionnaires, consentements signés et autres documents relatifs à l'étude seront archivés en sécurité dans un local spécifique à l'archivage des données, dont l'accès est limité au personnel d'Epicentre autorisé.

8.5. Bénéfices pour le patient

L'accord de participation à l'étude sera indépendant de l'accord de prise en charge dans un programme nutritionnel. Si un parent ou responsable légal refuse de participer à l'étude, son enfant pourra bénéficier de la prise en charge habituelle prévue pour les enfants sévèrement malnutris, et cette décision n'aura en aucun cas d'impact négatif sur son suivi.
Si l'enfant est inclus dans l'étude, il bénéficiera d'un suivi renforcé de son statut nutritionnel, et d'une prise en charge d'éventuelles pathologies intercurrentes plus précoces.

9. Calendrier de l'étude

Le calendrier prévisionnel de l'étude est décrit au tableau 2. Des modifications dans la planification des activités sont à prévoir en fonction des dates d'enregistrements et de distributions des partenaires du PAM et de Forsani/MSF-OCP.

Tableau 2 : Calendrier prévisionnel des activités liées à l'étude de mai 2011 à janvier 2013.

	2011								2012												2013
Mois	Mai	Juin	Juil.	Août	Sept	Oct	Nov	Déc	Jan	Fév	Mars	Avr	Mai	Juin	Juil	Août	Sept	Oct	Nov	Déc	Jan
Rédaction du protocole	■																				
Autorisations nationales		■																			
Autorisations locales		■																			
Recrutement du personnel		■																			
Sélection/inclusion des villages		■																			
Formation du personnel		■																			
Phase pilote		■																			
Inclusion des enfants		■																			
Sensibilisation de la population		■	■	■	■	■	■	■	■	■	■	■	■	■	■	■	■	■			
Distributions et suivi			■	■	■	■	■	■	■	■	■	■	■	■	■	■	■	■			
Suivi au sein des ménages (bras E, F et G)							■							■					■		
Saisie des données		■	■	■	■	■	■	■	■	■	■	■	■	■	■	■	■	■	■		
Analyses et rapports intermédiaires							■	■					■	■							
Analyses et rapport final																				■	■

10. Partenariat

Les partenaires opérationnels

L'étude sera menée principalement par Epicentre en collaboration avec la PAM, FORSANI et une institution de micro-finance ASUSU.

EPICENTRE sera responsable de :

- l'écriture du protocole ;
- l'identification des bénéficiaires (suppléments alimentaires, rations de protection en vivres et en argent) ;
- les distributions des suppléments alimentaires et des rations de protection en vivres;
- la collecte des données auprès des enfants et des ménages ;
- la supervision des procédures de l'étude ;
- la saisie et l'analyse des données ;
- l'écriture des rapports ;
- la valorisation scientifique des résultats.

Le PAM sera impliqué dans la rédaction du protocole. Il sera en charge de fournir et d'approvisionner Epicentre en intrants (suppléments alimentaires pour enfants et rations de protection en vivres) et l'institution de micro-finance ASUSU en fonds.

FORSANI prendra en charge les enfants sévèrement malnutris qui seront référés par les équipes Epicentre. Cette prise en charge sera effectuée au sein des structures de santé soutenues dans le cadre du programme commun FORSANI/MSF-OCP (CRENAS et CRENI).

ASUSU sera en charge de distribuer mensuellement l'argent liquide aux femmes des ménages identifiés comme bénéficiaires par Epicentre et rédigera un rapport d'activités conformément aux exigences du PAM.

Les partenaires financiers

MSF-OCP et le PAM seront impliqués dans le financement de l'étude.

11. Ressources

11.1. Ressources humaines

Un investigateur principal, basé à Epicentre Paris, est nommé pour toute la période de préparation et de déroulement de l'étude ; il assurera la supervision de l'étude en collaboration avec la coordination d'Epicentre au Niger. Il rédigera les rapports et articles scientifiques en collaboration avec les autres participants à l'étude

Un Assistant de Recherche Clinique (ARC) sera employé à plein temps par Epicentre au Niger pour tout le déroulement de l'étude. Il sera chargé de la supervision des activités au quotidien et de la formation continue des équipes. Il communiquera régulièrement avec les autorités locales sur le

déroulement du projet. Il informera l'investigateur principal de l'avancée de l'étude, et plus particulièrement lors de la moindre incertitude, difficulté, ou entorse au protocole. Il écrira par ailleurs un rapport mensuel détaillant les activités et les éventuels problèmes rencontrés.

Les inclusions, les distributions et les collectes de données seront effectués par des équipes d'assistants nutritionnels employés par Epicentre (40 personnes) et préalablement formés aux procédures de l'étude. Chaque équipe sera coordonnée par un chef d'équipe.

La saisie de données se déroulera au sein des bureaux d'Epicentre à Maradi par une équipe de 10 opérateurs de saisie, encadrée et supervisée par un gestionnaire de données expérimenté.

Un assistant logistique et un assistant administration/finances employés par Epicentre seront en charge de mettre en place toutes les procédures logistiques, administratives et financières pour le bon déroulement de l'étude. Ils seront encadrés et supervisés par un responsable logistique et un responsable administration/finances.

Les entrepôts de vivres seront sécurisés et gardés par du personnel Epicentre 24h/24. Leur gestion sera assurée par des magasiniers.

11.2. Ressources logistiques

Les équipes d'assistants nutritionnels se déplaceront entre les villages ou les sites de distribution à bord de véhicules de location.

Des entrepôts seront loués à Madarounfa, à Dan Issa et à Maradi pendant toute la durée de l'étude afin de conserver les intrants dans de bonnes conditions.

Les intrants seront transportés des stocks du PAM à Maradi vers les entrepôts Epicentre puis vers les sites de distribution par des camions ou des pick-up loués à cette occasion.

11.3. Ressources financières

L'étude sera financée par MSF-OCP et le PAM.

12. Communication des résultats

Deux rapports intermédiaires (décembre 2011 et juin 2012) puis un rapport final (janvier 2013) seront rédigés par Epicentre et communiqués au Comité Consultatif National d'Ethique du Niger, au Ministère de la Santé Publique du Niger, au PAM, à FORSANI et à MSF-OCP.

Epicentre veillera également à ce que les résultats soient communiqués aux autorités sanitaires locales (Direction Régionale de la Santé Publique et Equipe Cadre du District de Madarounfa).

Un article scientifique sera préparé et soumis pour publication dans les journaux scientifiques internationaux. Les résultats pourront également être présentés dans des conférences internationales.

13. Références

[1] UNICEF, *Tracking progress on child and maternal nutrition: A survival and development priority*, New-York, USA: UNICEF, 2009.

[2] D.L. Pelletier and E.A. Frongillo, “Changes in child survival are strongly associated with changes in malnutrition in developing countries.,” *The Journal of nutrition*, vol. 133, Jan. 2003, pp. 107-19.

[3] L.E. Caulfield, M. de Onis, M. Blössner, and R.E. Black, “Undernutrition as an underlying cause of child deaths associated with diarrhea, pneumonia, malaria, and measles.,” *The American journal of clinical nutrition*, vol. 80, Jul. 2004, pp. 193-8.

[4] J. Bryce, D. Coitinho, I. Darnton-Hill, D. Pelletier, and P. Pinstrup-Andersen, “Maternal and child undernutrition: effective action at national level,” *The Lancet*, vol. 371, Feb. 2008, pp. 510-526.

[5] *Déclaration commune de l’Organisation mondiale de la santé (OMS), du Programme alimentaire mondial (PAM), du Comité permanent de la nutrition du système des Nations Unies (CPN) et de le Fonds des Nations Unies pour l'enfance (UNICEF)*.

[6] FOOD AND AGRICULTURE ORGANIZATION OF THE UNITED NATIONS, “Undernourishment around the world in 2010,” 2010, pp. 8-11.

[7] Organisation des Nations Unies, “Rapport sur les objectifs du Millénaire,” 2010.

[8] United Nations Development Programme, “Human development report. The Real Wealth of Nations: Pathways to Human Development,” 2010.

[9] S.E. Wuehler and A. Biga Hassoumi, “Situational analysis of infant and young child nutrition policies and programmatic activities in Niger.,” *Maternal & child nutrition*, vol. 7 Suppl 1, Apr. 2011, pp. 133-56.

[10] S. Adu-Afarwuah, A. Lartey, K.H. Brown, S. Zlotkin, A. Briend, and K.G. Dewey, “Randomized comparison of 3 types of micronutrient supplements for home fortification of complementary foods in Ghana: effects on growth and motor development.,” *The American journal of clinical nutrition*, vol. 86, Aug. 2007, pp. 412-20.

[11] J.C. Phuka, K. Maleta, C. Thakwalakwa, Y.B. Cheung, A. Briend, M.J. Manary, and P. Ashorn, “Complementary feeding with fortified spread and incidence of severe stunting in 6- to 18-month-old rural Malawians.,” *Archives of pediatrics & adolescent medicine*, vol. 162, Jul. 2008, pp. 619-26.

[12] J.C. Phuka, K. Maleta, C. Thakwalakwa, Y.B. Cheung, A. Briend, M.J. Manary, and P. Ashorn, “Postintervention growth of Malawian children who received 12-mo dietary complementation with a lipid-based nutrient supplement or maize-soy flour.,” *The American journal of clinical nutrition*, vol. 89, Jan. 2009, pp. 382-90.

[13] E.H.I. Diop, N.I. Dossou, M.M. Ndour, A. Briend, and S. Wade, “Comparison of the efficacy of a solid ready-to-use food and a liquid, milk-based diet for the rehabilitation of severely malnourished children: a randomized trial.,” *The American journal of clinical nutrition*, vol. 78, Aug. 2003, pp. 302-7.

[14] A. Briend, “Possible use of spreads as a food diet for improving the diets of infants and young children.,” *Food and nutrition bulletin*, vol. 23, Sep. 2002, pp. 239-43.

[15] H. Sandige, M.J. Ndekha, A. Briend, P. Ashorn, and M.J. Manary, “Home-based treatment of malnourished Malawian children with locally produced or imported ready-to-use food.,” *Journal of pediatric gastroenterology and nutrition*, vol. 39, Aug. 2004, pp. 141-6.

[16] M.J. Manary, M.J. Ndkeha, P. Ashorn, K. Maleta, and A. Briend, “Home based therapy for severe malnutrition with ready-to-use food.,” *Archives of disease in childhood*, vol. 89, Jun. 2004, pp. 557-61.

[17] D.K. Matilsky, K. Maleta, T. Castleman, and M.J. Manary, “Supplementary feeding with fortified spreads results in higher recovery rates than with a corn/soy blend in moderately wasted children.,” *The Journal of nutrition*, vol. 139, Apr. 2009, pp. 773-8.

[18] C. Navarro-colorado, *A Retrospective Study of Emergency Supplementary Feeding Programmes*, 2007.

[19] M.P. Patel, H.L. Sandige, M.J. Ndekha, A. Briend, P. Ashorn, and M.J. Manary, “Supplemental feeding with ready-to-use therapeutic food in Malawian children at risk of malnutrition.,” *Journal of health, population, and nutrition*, vol. 23, Dec. 2005, pp. 351-7.

[20] V. Gaboulaud, N. Dan-Bouzoua, C. Brasher, G. Fedida, B. Gergonne, and V. Brown, “Could nutritional rehabilitation at home complement or replace centre-based therapeutic feeding programmes for severe malnutrition?,” *Journal of tropical pediatrics*, vol. 53, Feb. 2007, pp. 49-51.

[21] A. Briend, R. Lacsala, C. Prudhon, B. Mounier, Y. Grellety, and M.H. Golden, “Ready-to-use therapeutic food for treatment of marasmus.,” *Lancet*, vol. 353, May. 1999, pp. 1767-8.

[22] S. Collins and K. Sadler, “Outpatient care for severely malnourished children in emergency relief programmes: a retrospective cohort study.,” *Lancet*, vol. 360, Dec. 2002, pp. 1824-30.

[23] M.T. Ruel, P. Menon, J.-P. Habicht, C. Loechl, G. Bergeron, G. Pelto, M. Arimond, J. Maluccio, L. Michaud, and B. Hankebo, “Age-based preventive targeting of food assistance and behaviour change and communication for reduction of childhood undernutrition in Haiti: a cluster randomised trial,” *The Lancet*, vol. 371, Feb. 2008, pp. 588-595.

[24] I. Defourny, A. Minetti, G. Harczi, S. Doyon, S. Shepherd, M. Tectonidis, J.-H. Bradol, and M. Golden, “A large-scale distribution of milk-based fortified spreads: evidence for a new approach in regions with high burden of acute malnutrition.,” *PloS one*, vol. 4, Jan. 2009, p. e5455.

[25] S. Isanaka, N. Nombela, A. Djibo, M. Poupard, D. Van Beckhoven, V. Gaboulaud, P.J. Guerin, and R.F. Grais, “Effect of preventive supplementation with ready-to-use therapeutic food on the nutritional status, mortality, and morbidity of children aged 6 to 60 months in Niger: a

cluster randomized trial.," *JAMA : the journal of the American Medical Association*, vol. 301, Jan. 2009, pp. 277-85.

[26] M. a Ciliberto, H. Sandige, M.J. Ndekha, P. Ashorn, A. Briend, H.M. Ciliberto, and M.J. Manary, "Comparison of home-based therapy with ready-to-use therapeutic food with standard therapy in the treatment of malnourished Malawian children: a controlled, clinical effectiveness trial.," *The American journal of clinical nutrition*, vol. 81, Apr. 2005, pp. 864-70.

[27] K. Maleta, J. Kuittinen, M.B. Duggan, A. Briend, M. Manary, J. Wales, T. Kulmala, and P. Ashorn, "Supplementary feeding of underweight, stunted Malawian children with a ready-to-use food.," *Journal of pediatric gastroenterology and nutrition*, vol. 38, Feb. 2004, pp. 152-8.

[28] H. Kuusipalo, K. Maleta, A. Briend, M. Manary, and P. Ashorn, "Growth and change in blood haemoglobin concentration among underweight Malawian infants receiving fortified spreads for 12 weeks: a preliminary trial.," *Journal of pediatric gastroenterology and nutrition*, vol. 43, Oct. 2006, pp. 525-32.

[29] V.L. Flax, C. Thakwalakwa, J. Phuka, U. Ashorn, Y.B. Cheung, K. Maleta, and P. Ashorn, "Malawian mothers' attitudes towards the use of two supplementary foods for moderately malnourished children.," *Appetite*, vol. 53, Oct. 2009, pp. 195-202.

[30] L. Galpin, C. Thakwalakwa, J. Phuka, P. Ashorn, K. Maleta, W.W. Wong, and M.J. Manary, "Breast milk intake is not reduced more by the introduction of energy dense complementary food than by typical infant porridge.," *The Journal of nutrition*, vol. 137, Jul. 2007, pp. 1828-33.

[31] C. Hoppe, G.S. Andersen, S. Jacobsen, C. Mølgaard, H. Friis, P.T. Sangild, and K.F. Michaelsen, "The use of whey or skimmed milk powder in fortified blended foods for vulnerable groups.," *The Journal of nutrition*, vol. 138, Jan. 2008, p. 145S-161S.

[32] S. Adu-Afarwuah, A. Lartey, K.H. Brown, S. Zlotkin, A. Briend, and K.G. Dewey, "Home fortification of complementary foods with micronutrient supplements is well accepted and has positive effects on infant iron status in Ghana.," *The American journal of clinical nutrition*, vol. 87, Apr. 2008, pp. 929-38.

[33] A. Lartey, A. Manu, K.H. Brown, J.M. Peerson, and K.G. Dewey, "A randomized, community-based trial of the effects of improved, centrally processed complementary foods on growth and micronutrient status of Ghanaian infants from 6 to 12 mo of age.," *The American journal of clinical nutrition*, vol. 70, Sep. 1999, pp. 391-404.

[34] S. de Pee and M.W. Bloem, "Current and potential role of specially formulated foods and food supplements for preventing malnutrition among 6- to 23-month-old children and for treating moderate malnutrition among 6- to 59-month-old children.," *Food and nutrition bulletin*, vol. 30, Sep. 2009, pp. S434-63.

[35] J.L. Leroy, P. Gadsden, S. Rodríguez-Ramírez, and T.G. de Cossío, "Cash and in-kind transfers in poor rural communities in Mexico increase household fruit, vegetable, and micronutrient consumption but also lead to excess energy consumption.," *The Journal of nutrition*, vol. 140, Mar. 2010, pp. 612-7.

[36] Save The Children UK, *How cash transfers can improve the nutrition of the poorest children. Evaluation of a pilot safety net programme in southern Niger*, 2009.

[37] D. Sridhar and A. Duffield, *A Review of the Impact of Cash Transfer Programmes on Child Nutrition Status and some Implications for Save the Children UK programmes*, London: 2006.

[38] O. Attanasio, *The short-term impact of a conditional cash subsidy on child health and nutrition in Colombia*, 2005.

Annexes

Annexe 1. Composition en valeur énergétique et nutriments de différents compléments alimentaires

Annexe 2. Description des différents groupes d'intervention de l'étude

Annexe 3. Zone d'intervention de l'étude, Niger, Région de Maradi, district sanitaire de Madarounfa

Annexe 4. Localisation des zones d'intervention, District sanitaire de Madarounfa, aires de santé de Madarounfa, Dan Issa et Tofa

Annexe 5. Fiche village

Annexe 6. Lettre d'information et consentement éclairé pour le chef de village

Annexe 7. Lettre d'information et consentement éclairé pour le représentant légal de l'enfant

Annexe 8. Déclaration d'Helsinki de l'Association Médicale Mondiale

Annexe 9. Epicentre au Niger

Annexe 1. Composition en valeur énergétique et nutriments de différents compléments alimentaires

Produit	Plumpy'Nut®		Plumpy'Doz®		Supplementary'Plumpy®		Corn-Soy Blend Plus Plus CSB++	
Indication	Aliment thérapeutique de la malnutrition aiguë sévère à partir de 6 mois		Aliment de complément pour enfant 6-24 mois		Aliment de supplément pour enfant atteint de malnutrition aiguë modérée		Aliment de complément pour enfant 6-24 mois et enfant atteint de malnutrition aiguë modérée	
Ingrédients	Pâte d'arachide, sucre, huile végétale, lait écrémé en poudre, petit lait, maltodextrine, complexe vitamines et minéraux		Pâte d'arachide, sucre, huile végétale, lait écrémé en poudre, petit lait, maltodextrine, cacao, complexe vitamines et minéraux		Pâte d'arachide, sucre, huile végétale, petit lait, protéines extraites soja, maltodextrine, cacao, complexe vitamines et minéraux		Farine de mais, graines de soja décortiquées, sucre, huile végétale, lait écrémé en poudre, complexe vitamines et minéraux	
Date fiche technique	1996		2 février 2010		20 février 2009		30 mars 2011	
Valeur nutritionnelle	Par ration journalière<br>1 sachet (92g)	Par 100 g	Par ration journalière<br>3 cuillères (46g)	Par 100 g	Par ration journalière<br>1 sachet (92g)	Par 100 g	Par ration journalière<br>200g	Par 100 g
Kcal	501	545	247	587	500	550	820	410
Protéines (% énergie)	12,1	13,2	10	12	10	10	32	16
Lipides (% énergie)	58,6	63,7	58	60	60	60	18	9
Protéines g	12,5	13,6	5,9	13,3	12,5	15	24,8	12,4
Lipides g	32,8	35,7	16	36,2	32,9	38	7,6	3,8
Vit A µg	840	910	400	1037	840	1000	3328	1664
Vit D µg	14,7	16	-	-	15	22.4	8	4
Vit E mg	18,4	20	6	16.8	18.4	25	16.6	8.3
Thiamine (B1) mg	0,6	0,6	0,5	2,2	0,6	1,2	0,3	0,1

	Plumpy'Nut®		Plumpy'Doz®		Supplementary'Plumpy®		CSB++	
	Par ration journalière	Par 100 g	Par ration journalière	Par 100 g	Par ration journalière	Par 100 g	Par ration journalière	Par 100 g
**Riboflavine (B2) mg**	1,7	1,8	0,5	1.32	1,7	2	0.9	0.5
**Vit B6 mg**	0,6	0,6	0,5	1.32	0,6	0.7	3.4	1.7
**Vit B12 µg**	1,7	1,8	0,9	2.5	1.7	2	4	2
**Vit C mg**	48,8	53	30	162	49	132	200	100
**Acide Folique (B9) µg**	193,2	210	160	432	193	252	120	60
**Niacine (B3) mg**	4,9	5,3	6	14.8	5	5.8	9.6	4.8
**Vit K µg**	19,3	21	-	-	19,3	25	200	100
**Biotine (B8) µg**	59,8	65	-	-	60	72	-	-
**Acide Pantoth.(B5) mg**	2,9	3,1	2,0	.6	2.9	3.4	13.4	6.7
**Calcium mg**	276	300	387	920	276	330	260	130
**Phosphore mg**	276	300	275	653	276	330	400	200
**Potassium mg**	1022	1111	310	737	511	610	800	400
**Magnésium mg**	84,6	92	60	142	85	101	-	-
**Zinc mg**	12,9	14	9,0	21.3	12.9	15	10	5
**Cuivre mg**	1,7	1.8	0,3	0.7	1.6	2	-	-
**Sodium mg**	<267	<290	Non précisé	Non précisé	<267	<290	Non précisé	Non précisé
**Fer mg**	10,6	11,5	9	21.3	10.6	12.6	8	4
**Iode µg**	92	100	90	214	92	110	80	40
**Sélénium µg**	27,6	30	17	40.3	28	33	-	-

Annexe 2. Description des différents groupes d'intervention de l'étude

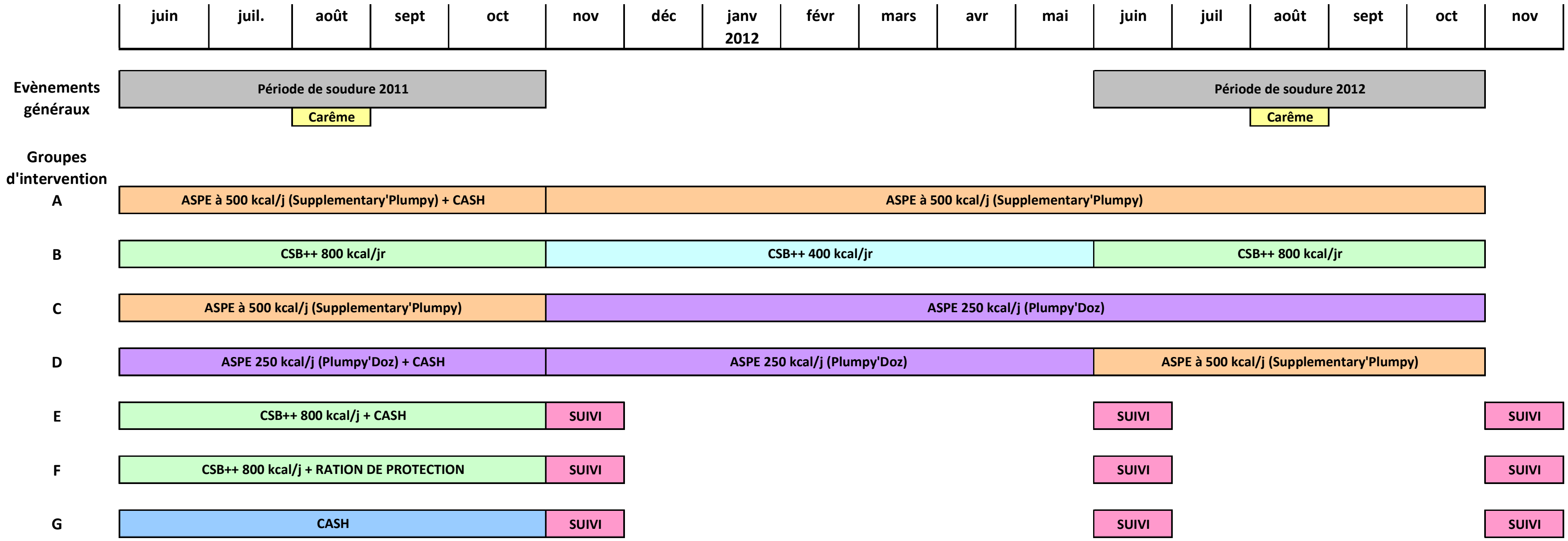

Annexe 3. Zone d'intervention de l'étude, Niger, Région de Maradi, district sanitaire de Madarounfa

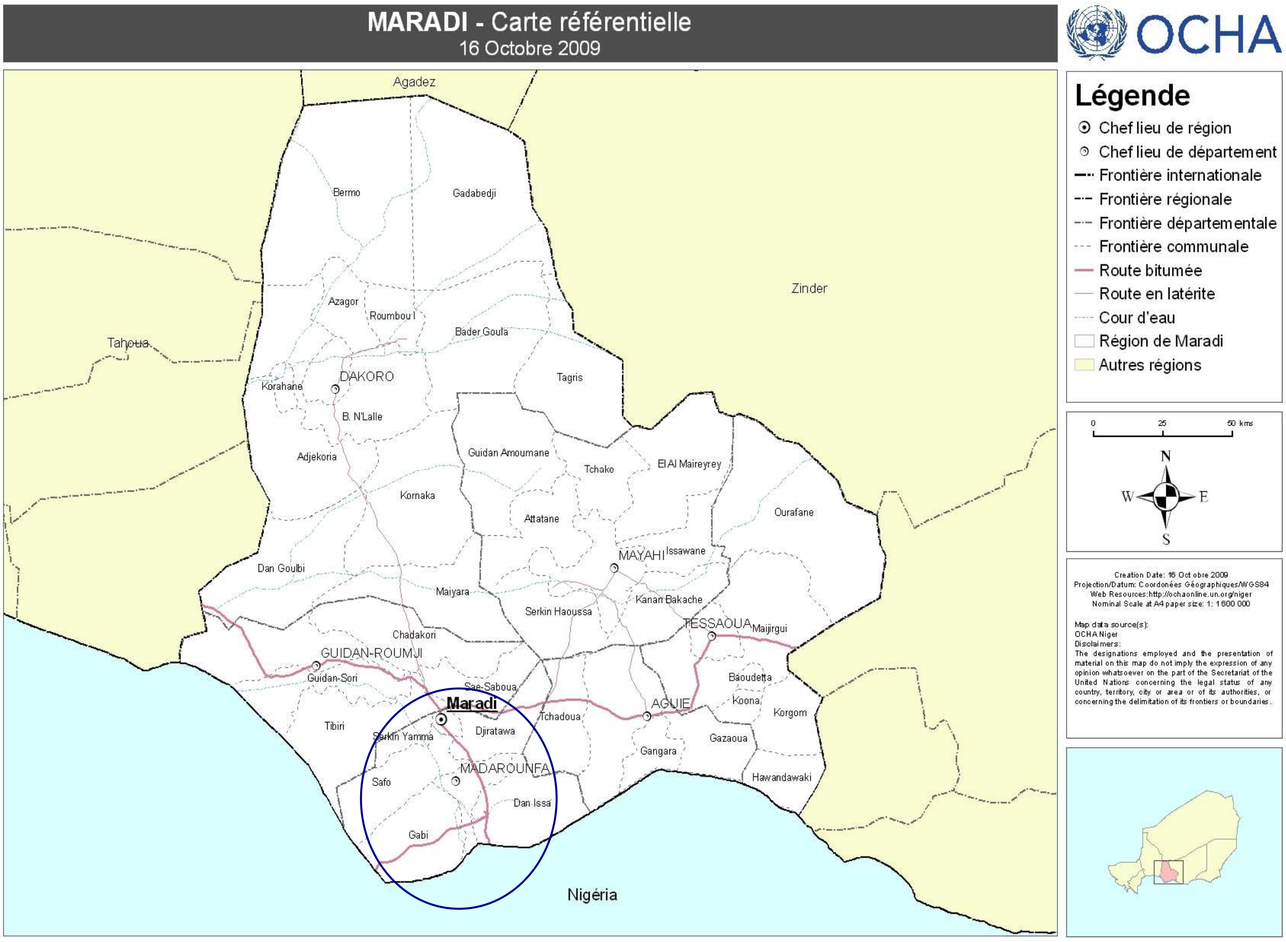

Annexe 4 : Localisation des zones d'intervention, District sanitaire de Madarounfa, aires de santé de Madarounfa, Dan Issa et Tofa

Les zones grisées correspondent aux groupements de villages dans lesquels auront lieu les interventions

.

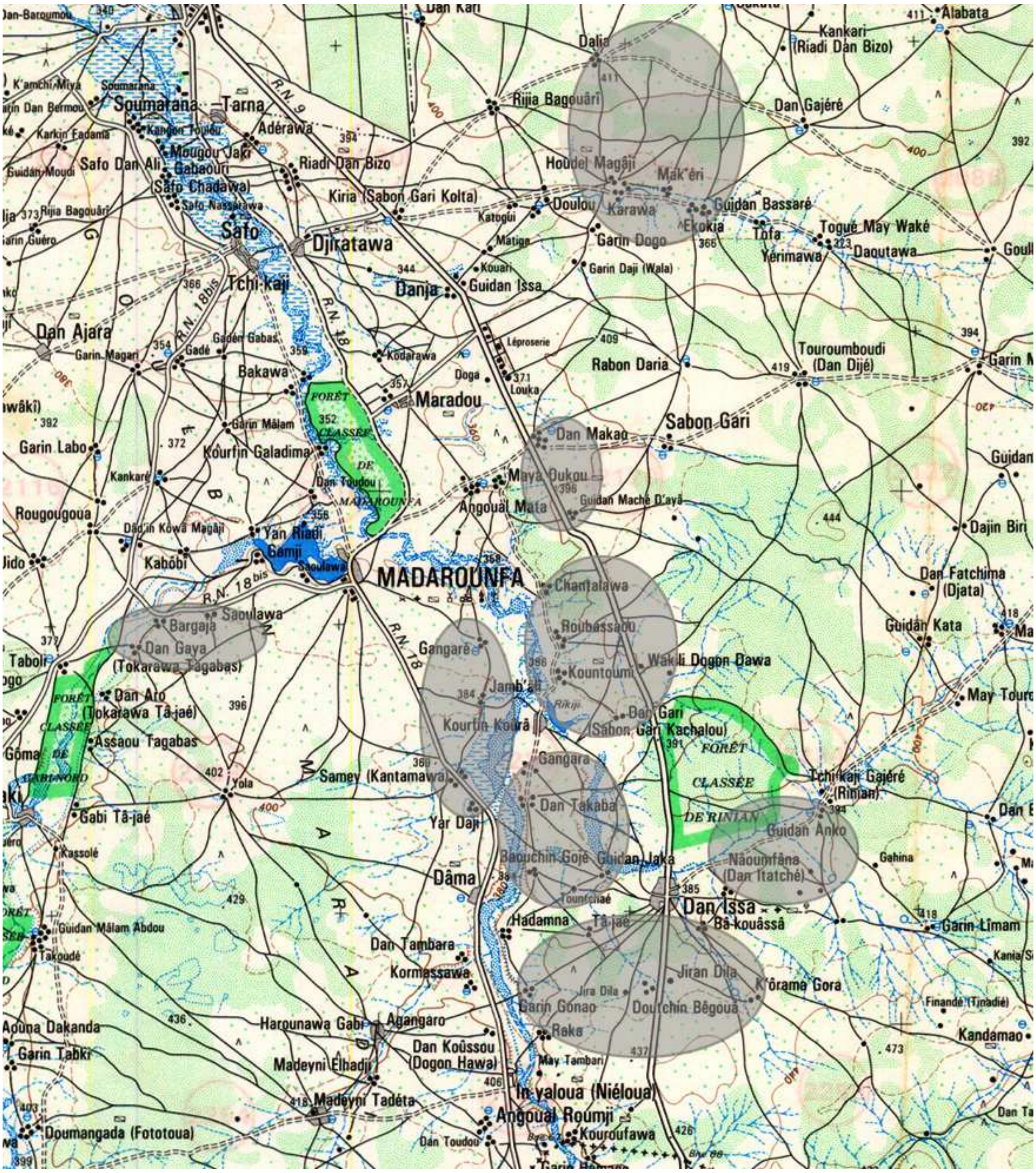

Annexe 5. Fiche village

Village :_______________ Aire de santé :__________ Commune : _________________

Nom du chef de village : ____________________________________

Date : ___/____/____

Caractéristiques relatives à la population
**Nombre d'habitants :** ________________
**Nombre de ménages** : ________________
**Nombre d'enfants :** <5 ans : _____________
<2 ans : _____________
**Principales sources des revenus des habitants (√) :**
❒ Ventes des produits des récoltes
❒ Petit commerce ou artisanat
❒ Elevage
❒ Travail journalier
❒ Exode
**Migration saisonnière des habitants : (√)**
❒ Importante : Plus de la moitié des habitants
❒ Moyenne : Entre un quart et la moitié
❒ Rare : Moins d'un quart des habitants

Caractéristiques relatives aux infrastructures
**Structure de santé** visitée habituellement :
______________________ distance : ______km
Distance de la **route principale** : _________ **km**
Distance du **marché** hebdomadaire : _______ **km**

Caractéristiques relatives à l'accès à l'eau et la nourriture
Accès à **l'eau potable** : ❒ Oui
❒ Non
Nombre de **puits/forages** : _________
Distance aux puits/forages : _______**km**
**Cultures** : ❒ Oui
❒ Non

Evènement important ayant affecté tout ou partie du village
**Evènement** : ____
**Date de l'évènement** : ____/_____/_____ ou **Période** : du ___/_____/_____ au ____/____/____
**Conséquences** agricoles, sanitaires et infrastructures:

Annexe 6. Lettre d'information et consentement éclairé pour le chef de village

Lettre d'information du chef du village
sur l'étude d'évaluation de différentes stratégies de prévention de la malnutrition

---

But de l'étude
Epicentre souhaite étudier différentes stratégies pour prévenir la malnutrition des jeunes enfants. Il existe en effet différents produits spécifiquement conçus pour répondre à leurs besoins, améliorer leur croissance et leur état de santé. De plus, il existe plusieurs moyens pour aider le ménage pendant la période de soudure, soit par de l'argent, soit par des vivres. Nous comparerons les différentes stratégies et verrons si l'action préventive proposée est efficace pour prévenir la malnutrition. En fonction des résultats, la stratégie apparaissant la plus efficace sera recommandée pour l'avenir.

Déroulement de l'étude
Nous souhaitons sélectionner tous les enfants vivant de façon permanente dans votre village et ayant une taille entre 60 et 80 cm, ce qui correspond à un âge entre 6 et 23 mois. Nous les suivrons pendant 18 mois.
Pour chacun des 50 villages inclus dans l'étude, la stratégie préventive sera choisie au hasard. Vous serez informé à l'avance de la stratégie choisie pour votre village.
Pendant la durée de l'étude, tous les enfants entre 60 et 80 cm recevront soit un aliment prêt à l'emploi à base de pâte d'arachide (Plumpy'Doz ou Supplementary'Plumpy), soit une farine enrichie (CSB++). Le produit pourra être modifié spécifiquement au cours du suivi.
Il se peut que votre village soit aussi bénéficiaire de distributions d'argent ou de vivres à l'intention des ménages ayant un enfant suivi dans l'étude. Si c'est le cas, il se peut qu'Epicentre intervienne dans votre communauté pendant seulement les 5 prochains mois.
Certains villages recevront uniquement de l'argent liquide pendant les 5 prochains mois spécifiquement pour les ménages avec un enfant entre 60 et 80 cm mais sans distribution d'aliments.
La santé des enfants inclus sera surveillée tous les mois pendant les distributions. Nous mesurerons leur poids, leur taille et leur périmètre brachial. Nous poserons également des questions à la mère ou au chef de ménage concernant la santé et l'alimentation de l'enfant. Il pourra être référé aux centres de réhabilitation nutritionnelle le plus proche en cas de malnutrition sévère. Nous irons également dans les ménages pour recueillir des informations sur leurs habitudes alimentaires et leurs sources de revenus.
Si un enfant est absent lors d'une distribution, une équipe passera à son domicile pour recueillir les informations sur l'enfant mais il ne recevra pas les aliments prévus.
Les nouveaux enfants qui atteignent la taille de 60cm au cours de l'étude pourront être inclus. Si un enfant atteint la taille de 80cm, il ne recevra plus de d'aliments et le suivi mensuel s'arrêtera.

Confidentialité
Nous vous assurons que les entretiens seront confidentiels. Le nom des enfants et parents ne seront pas utilisés lors de la saisie des données dans l'ordinateur, et personne en dehors des responsables de l'étude ne verra les informations du questionnaire ou les résultats.

Coût et paiement
Il n'y aura pas de compensation financière pour vous ou les familles participant à cette étude. L'ensemble du suivi et de la prise en charge des enfants est gratuit.

Libre choix de participation
Vous êtes libre d'accepter ou de refuser que votre village participe à cette étude, sans avoir à donner de raison. Vous avez également le droit de demander à faire sortir votre village de l'étude à tout moment.

Nous vous invitons à poser des questions au sujet de l'étude pour vous aider à décider d'y participer ou non.
Nous vous remercions très sincèrement pour votre collaboration.

En cas de problèmes ou de questions, merci de contacter :

Dr Issa Kanta	96 37 38 28
Abdoul-Aziz Mamaty	96 52 10 72
Bureau Epicentre à Maradi	20 41 02 82

Formulaire de consentement éclairé du chef de village

pour participer à l'étude d'évaluation de différentes stratégies de prévention de la malnutrition

---

Je soussigné (e)

Nom____Prénom____________________________

Chef du village de ______________________________

dans l'aire de santé de __________________________

déclare avoir lu la note d'information qui concerne l'évaluation de différentes stratégies de prévention de la malnutrition (ou quelqu'un me l'a lue).

J'ai compris les objectifs de l'étude et son déroulement. J'ai obtenu des réponses claires à toutes mes questions.

J'ai aussi compris que je peux refuser de faire participer mon village ou de retirer mon village de l'étude à tout moment sans avoir à fournir de raison et sans aucun préjudice ni reproche vis-à-vis de moi-même ou des habitants du village.

Je suis d'accord pour que mon village participe à l'étude sous les conditions présentées dans la note d'information.

Signature ou empreinte digitale : Date : _____ /_____/20__

Témoin du consentement éclairé<br>Je soussigné<br>Nom_____________________________________Prénom___________________________________<br>confirme que l'information a été correctement transmise et apparemment comprise par le chef de village et que le consentement éclairé a été donné en toute liberté.<br>Signature :

Personne recueillant le consentement

Je soussigné

Nom_____________________________________Prénom___________________________________

certifie avoir expliqué au(x) personne(s) mentionnée(s) ci-dessus la nature et les objectifs de l'étude, les bénéfices potentiels ainsi que les risques attendus relatifs à la participation à cette étude de recherche. J'ai répondu à toutes les questions qui m'ont été posées et j'ai assisté à la signature du document ci-dessus.

Signature: Date : _____ /_____/20__

Annexe 7. Lettre d'information et consentement éclairé pour le représentant légal de l'enfant

Lettre d'information du représentant légal de l'enfant
sur l'étude d'évaluation de différentes stratégies de prévention de la malnutrition

---

But de l'étude
Epicentre souhaite étudier différentes stratégies pour prévenir la malnutrition des jeunes enfants. Il existe en effet différents produits spécifiquement conçus pour répondre à leurs besoins, améliorer leur croissance et leur état de santé. De plus, il existe plusieurs moyens pour aider le ménage pendant la période de soudure, soit par de l'argent, soit par des vivres. Nous comparerons les différentes stratégies et verrons si l'action préventive proposée est efficace pour prévenir la malnutrition. En fonction des résultats, la stratégie apparaissant la plus efficace sera recommandée pour l'avenir. Nous avons sélectionné 50 villages pour l'étude et la stratégie préventive a été choisie au hasard.

Déroulement de l'étude (*à adapter en fonction du groupe d'intervention du village)*
Nous souhaitons sélectionner tous les enfants vivant de façon permanente dans votre village avec une taille entre 60 et 80 cm.

L'intervention qui aura lieu dans votre village est la suivante :

Bras A : Tous les mois, pendant 18 mois, votre enfant recevra un aliment prêt à l'emploi à base de pâte d'arachide (Supplementary'Plumpy). Nous vous donnerons 30 sachets à chaque distribution pour une consommation d'un sachet par jour. Pendant les 5 premiers mois (de juin à octobre), vous recevrez également de l'argent liquide (25 000 FCFA) pour votre ménage qu'il faudra aller récupérer auprès d'une banque (ASUSU).
La santé de votre enfant sera surveillée tous les mois. Nous mesurerons son poids, sa taille et son périmètre brachial. Nous vous poserons également des questions concernant sa santé et son alimentation. Il pourra être référé aux centres de réhabilitation nutritionnelle le plus proche en cas de malnutrition sévère. Nous nous rendrons également chez vous pour recueillir des informations sur vos habitudes alimentaires et vos sources de revenus. Si votre enfant est absent lors d'une distribution, une équipe passera à votre domicile pour recueillir les informations sur votre enfant mais vous ne recevrez pas les aliments prévus. Si votre enfant dépasse la taille de 80 cm avant la fin de l'étude, il ne recevra plus d'aliments et le suivi s'arrêtera.

Bras B : Tous les mois, pendant 18 mois, votre enfant recevra une farine enrichie (CSB++). Nous vous donnerons 3 ou 6kg à chaque distribution pour une consommation de 100 à 200g par jour. La santé de votre enfant sera surveillée tous les mois. Nous mesurerons son poids, sa taille et son périmètre brachial. Nous vous poserons également des questions concernant sa santé et son alimentation. Il pourra être référé aux centres de réhabilitation nutritionnelle le plus proche en cas de malnutrition sévère. Nous nous rendrons également chez vous pour recueillir des informations sur vos habitudes alimentaires et vos sources de revenus. Si votre enfant est absent lors d'une distribution, une équipe passera à votre domicile pour recueillir les informations sur votre enfant mais vous ne recevrez pas les aliments prévus. Si votre enfant dépasse la taille de 80 cm avant la fin de l'étude, il ne recevra plus d'aliments et le suivi s'arrêtera.

Bras C : Tous les mois, pendant 5 mois, votre enfant recevra un aliment prêt à l'emploi à base de pâte d'arachide (Supplementary'Plumpy). Nous vous donnerons 30 sachets à chaque distribution pour une consommation d'un sachet par jour. A partir de novembre, il recevra un autre produit prêt à l'emploi à base de pâte d'arachide (Plumpy'Doz). Nous vous donnerons alors 4 pots pour une consommation d'un pot par semaine. La santé de votre enfant sera surveillée tous les mois. Nous mesurerons son poids, sa taille et son périmètre brachial. Nous vous poserons également des questions concernant sa santé et son alimentation. Il pourra être référé aux centres de réhabilitation nutritionnelle le plus proche en cas de malnutrition sévère. Nous nous rendrons également chez vous pour recueillir des informations sur vos habitudes alimentaires et vos sources de revenus. Si votre enfant est absent lors d'une distribution, une équipe passera à votre domicile pour recueillir les informations sur votre enfant mais vous ne recevrez pas les aliments prévus. Si votre enfant dépasse la taille de 80 cm avant la fin de l'étude, il ne recevra plus d'aliments et le suivi s'arrêtera.

Bras D : Tous les mois, pendant 12 mois, votre enfant recevra un aliment prêt à l'emploi à base de pâte d'arachide (Plumpy'Doz). Nous vous donnerons alors 4 pots pour une consommation d'un pot par semaine. A partir de l'année prochaine, il recevra un autre aliment prêt à l'emploi à base de pâte d'arachide (Supplementary'Plumpy). Nous vous donnerons 30 sachets à chaque distribution pour une consommation d'un sachet par jour. Pendant les 5 premiers mois (de juin à octobre), vous recevrez également de l'argent liquide pour votre ménage qu'il faudra aller récupérer auprès d'une banque (ASUSU). La santé de votre enfant sera surveillée tous les mois. Nous mesurerons son poids, sa taille et son périmètre brachial. Nous vous poserons également des questions concernant sa santé et son alimentation. Il pourra être référé aux centres de réhabilitation nutritionnelle le plus proche en cas de malnutrition sévère. Nous nous rendrons également chez vous pour recueillir des informations sur vos habitudes alimentaires et vos sources de revenus. Si votre enfant est absent lors d'une distribution, une équipe passera à votre domicile pour recueillir les informations sur votre enfant mais vous ne recevrez pas les aliments prévus. Si votre enfant dépasse la taille de 80 cm avant la fin de l'étude, il ne recevra plus d'aliments et le suivi s'arrêtera.

Bras E : Tous les mois, pendant 5 mois, votre enfant recevra une farine enrichie (CSB++). Nous vous donnerons 6 kg à chaque distribution pour une consommation de 200g par jour. Vous recevrez également de l'argent liquide pour votre ménage (25 000 FCFA) qu'il faudra aller récupérer auprès d'une banque (ASUSU). La santé de votre enfant sera surveillée tous les mois pendant les 5 premiers mois. Puis une équipe Epicentre passera à votre domicile en novembre 2011, puis en juin 2012 et en novembre 2012. Nous mesurerons le poids de votre enfant, sa taille et son périmètre brachial. Nous vous poserons également des questions concernant sa santé et son alimentation. Il pourra être référé aux centres de réhabilitation nutritionnelle le plus proche en cas de malnutrition sévère. Nous nous rendrons également chez vous pour recueillir des informations sur vos habitudes alimentaires et vos sources de revenus. Si votre enfant est absent lors d'une distribution, une équipe passera à votre domicile pour recueillir les informations sur votre enfant mais vous ne recevrez pas les aliments prévus. Si votre enfant dépasse la taille de 80 cm avant la fin de l'étude, il ne recevra plus d'aliments et le suivi s'arrêtera.

Bras F : Tous les mois, pendant 5 mois, votre enfant recevra une farine enrichie (CSB++). Nous vous donnerons 6 kg à chaque distribution pour une consommation de 200g par jour. Vous recevrez également une ration de vivres pour votre ménage. La santé de votre enfant sera surveillée tous les mois pendant les 5 premiers mois. Puis une équipe Epicentre passera à votre domicile en novembre 2011, puis en juin 2012 et en novembre 2012. Nous mesurerons le poids de votre enfant, sa taille et son périmètre brachial. Nous vous poserons également des questions concernant sa santé et son alimentation. Il pourra être référé aux centres de réhabilitation nutritionnelle le plus proche en cas de malnutrition sévère. Nous nous rendrons également chez vous pour recueillir des informations sur vos habitudes alimentaires et vos sources de revenus. Si votre enfant est absent lors d'une distribution, une équipe passera à votre domicile pour recueillir les informations sur votre enfant mais vous ne recevrez pas les aliments prévus. Si votre enfant dépasse la taille de 80 cm avant la fin de l'étude, il ne recevra plus d'aliments et le suivi s'arrêtera.

Bras G : Tous les mois, pendant 5 mois, vous recevrez de l'argent liquide pour votre ménage (28 000 FCFA) qu'il faudra aller récupérer auprès d'une banque (ASUSU). La santé de votre enfant sera surveillée tous les mois pendant les 5 premiers mois. Puis une équipe Epicentre passera à votre domicile en novembre 2011, puis en juin 2012 et en novembre 2012. Nous mesurerons le poids de votre enfant, sa taille et son périmètre brachial. Nous vous poserons également des questions concernant sa santé et son alimentation. Il pourra être référé aux centres de réhabilitation nutritionnelle le plus proche en cas de malnutrition sévère. Nous nous rendrons également chez vous pour recueillir des informations sur vos habitudes alimentaires et vos sources de revenus. Si votre enfant est absent lors d'une distribution, une équipe passera à votre domicile pour recueillir les informations sur votre enfant mais vous ne recevrez pas les aliments prévus. Si votre enfant dépasse la taille de 80 cm avant la fin de l'étude, il ne recevra plus d'aliments et le suivi s'arrêtera.

Confidentialité
Nous vous assurons que les entretiens seront confidentiels. Le nom de votre enfant et le vôtre ne seront pas utilisés lors de la saisie des données dans l'ordinateur, et personne en dehors des responsables de l'étude ne verra les informations du questionnaire ou les résultats.

Coût et paiement
Vous ne recevrez pas d'argent pour participer à cette étude. L'ensemble du suivi et de la prise en charge de votre enfant est gratuit.

Libre choix de participation
Vous êtes libre d'accepter ou de refuser que votre enfant participe à cette étude, sans avoir à donner de raison. Vous avez également le droit de demander à faire sortir votre enfant de l'étude à tout moment.

Nous vous invitons à poser des questions au sujet de l'étude pour vous aider à décider d'y participer ou non.

Nous vous remercions très sincèrement pour votre collaboration.

Nous nous engageons à tenir confidentielles toutes les données recueillies sur votre famille.

Dr Issa KANTA

En cas de problèmes ou de questions, merci de contacter :

Dr Issa Kanta	96 37 38 28
Abdoul-Aziz Mamaty	96 52 10 72
Bureau Epicentre à Maradi	20 41 02 82

Formulaire de consentement éclairé du représentant légal de l'enfant
pour participer à l'étude d'évaluation de différentes stratégies de prévention de la malnutrition

---

Je soussigné (e)

Nom____Prénom_________________________________

Père ❒ Mère ❒ Autre ❒ Préciser :……………………………………………………………………………….

de l'enfant :

Nom____________________________________Prénom___________________________

déclare avoir lu la note d'information qui concerne l'évaluation de différentes stratégies de prévention de la malnutrition (ou quelqu'un me l'a lue).

J'ai compris les objectifs de l'étude et son déroulement. J'ai obtenu des réponses claires à toutes mes questions.

J'ai aussi compris que je peux refuser de faire participer mon enfant ou de retirer mon enfnat de l'étude à tout moment sans avoir à fournir de raison et sans aucun préjudice ni reproche vis-à-vis de moi-même ou de mon enfant.

Je suis d'accord pour que mon enfant participe à l'étude sous les conditions présentées dans la note d'information.

Signature ou empreinte digitale : Date : _____ /_____/20___

Témoin du consentement éclairé

Je soussigné

Nom_______________________________________Prénom___________________________________

confirme que l'information a été correctement transmise et apparemment comprise par la personne citée ci-dessus et que le consentement éclairé a été donné en toute liberté.

Signature :

Personne recueillant le consentement

Je soussigné

Nom_______________________________________Prénom___________________________________

certifie avoir expliqué au(x) personne(s) mentionnée(s) ci-dessus la nature et les objectifs de l'étude, les bénéfices potentiels ainsi que les risques attendus relatifs à la participation à cette étude de recherche. J'ai répondu à toutes les questions qui m'ont été posées et j'ai assisté à la signature du document ci-dessus.

Signature: Date : _____ /_____/20___

Annexe 8. Déclaration d'Helsinki de l'Association Médicale Mondiale

Principes éthiques applicables à la recherche médicale impliquant des êtres humains

Adoptée par la 18e Assemblée générale de l'AMM, Helsinki, Finlande, Juin 1964 et amendée par les
29e Assemblée générale de l'AMM, Tokyo, Octobre 1975
35e Assemblée générale de l'AMM, Venise, Octobre 1983
41e Assemblée générale de l'AMM, Hong Kong, Septembre 1989
48e Assemblée générale de l'AMM, Somerset West (Afrique du Sud), Octobre 1996
52e Assemblée générale de l'AMM, Edimbourg, Ecosse, Octobre 2000
53e Assemblée générale de l'AMM, Washington, Etats Unis, 2002 (ajout d'une note de clarification pour le paragraphe 29)
55e Assemblée générale de l'AMM, Tokyo, Japon 2004 (ajout d'une note de clarification concernant le paragraphe 30)
59e Assemblée générale de l'AMM, Séoul, Corée, Octobre 2008

INTRODUCTION

L'Association Médicale Mondiale (AMM) a élaboré la Déclaration d'Helsinki comme un énoncé de principes éthiques applicables à la recherche médicale impliquant des êtres humains, y compris la recherche sur du matériel biologique humain et sur des données identifiables.

La Déclaration est conçue comme un tout indissociable. Aucun paragraphe ne peut être appliqué sans tenir compte de tous les autres paragraphes pertinents.

Cette Déclaration s'adresse principalement aux médecins. L'AMM invite cependant les autres participants à la recherche médicale impliquant des êtres humains à adopter ces principes.

Le devoir du médecin est de promouvoir et de sauvegarder la santé des patients, y compris celles des personnes impliquées dans la recherche médicale. Le médecin consacre son savoir et sa conscience à l'accomplissement de ce devoir.

La Déclaration de Genève de l'AMM engage les médecins en ces termes : "La santé de mon patient prévaudra sur toutes les autres considérations" et le Code International d'Ethique Médicale déclare qu'un "médecin doit agir dans le meilleur intérêt du patient lorsqu'il le soigne".

Le progrès médical est basé sur la recherche qui, en définitive, doit comprendre des études impliquant des êtres humains. Des possibilités appropriées de participer à la recherche médicale devraient être offertes aux populations qui y sont sous- représentées.

Dans la recherche médicale impliquant des êtres humains, le bien-être de chaque personne impliquée dans la recherche doit prévaloir sur tous les autres intérêts.

L'objectif premier de la recherche médicale impliquant des êtres humains est de comprendre les causes, le développement et les effets des maladies et d'améliorer les interventions préventives, diagnostiques et thérapeutiques (méthodes, procédures et traitements). Même les meilleures interventions courantes doivent être évaluées en permanence par des recherches portant sur leur sécurité, leur efficacité, leur pertinence, leur accessibilité et leur qualité.

Dans la pratique médicale et la recherche médicale, la plupart des interventions comprennent des risques et des inconvénients.

La recherche médicale est soumise à des normes éthiques qui promeuvent le respect de tous les êtres humains et qui protègent leur santé et leurs droits. Certaines populations faisant l'objet de recherches sont particulièrement vulnérables et ont besoin d'une protection spéciale. Celles-ci incluent les personnes qui, d'elles-mêmes, ne sont pas en mesure de donner ou de refuser leur consentement et celles qui peuvent être vulnérables à la coercition ou à des influences indues.

Dans la recherche médicale impliquant des êtres humains, les médecins devraient tenir compte des normes et standards éthiques, légaux et réglementaires applicables dans leur propre pays ainsi que des normes et standards internationaux. Les protections garanties par la présente Déclaration aux personnes impliquées dans la recherche ne peuvent être restreintes ou exclues par aucune disposition éthique, légale ou réglementaire, nationale ou internationale.

PRINCIPES APPLICABLES A TOUS LES TYPES DE RECHERCHE MEDICALE

Il est du devoir des médecins participant à la recherche médicale de protéger la vie, la santé, la dignité, l'intégrité, le droit à l'auto-détermination, la vie privée et la confidentialité des informations des personnes impliquées dans la recherche.

La recherche médicale impliquant des êtres humains doit se conformer aux principes scientifiques généralement acceptés, se baser sur une connaissance approfondie de la littérature scientifique, sur d'autres sources pertinentes d'informations et sur des expériences appropriées en laboratoire et, le cas échéant, sur les animaux. Le bien être des animaux utilisés dans la recherche doit être respecté.

Une prudence particulière s'impose dans la conduite de recherches susceptibles de nuire à l'environnement.

La conception et la conduite de toutes les études impliquant des êtres humains doivent être clairement décrites dans un protocole de recherche. Ce protocole devrait contenir une déclaration sur les enjeux éthiques en question et indiquer comment les principes de la présente Déclaration ont été pris en considération. Le protocole devrait inclure des informations concernant le financement, les promoteurs, les affiliations institutionnelles, d'autres conflits d'intérêts potentiels, les incitations pour les personnes impliquées dans la recherche et les mesures prévues pour soigner et/ou dédommager celles ayant subis un préjudice en raison de leur participation à l'étude. Le protocole devrait mentionner les dispositions prévues après l'étude afin d'offrir aux personnes impliquées un accès aux interventions identifiées comme bénéfiques dans le cadre de l'étude ou à d'autres soins ou bénéfices appropriés.

Le protocole de recherche doit être soumis à un comité d'éthique de la recherche pour évaluation, commentaires, conseils et approbation avant que l'étude ne commence. Ce comité doit être indépendant du chercheur, du promoteur et de toute autre influence indue. Il doit prendre en considération les lois et réglementations du ou des pays où se déroule la recherche, ainsi que les normes et standards internationaux, mais ceux-ci ne doivent pas permettre de restreindre ou exclure l'une des protections garanties par la présente Déclaration aux personnes impliquées dans la recherche. Le comité doit avoir un droit de suivi sur les études en cours. Le chercheur doit fournir au comité des informations sur le suivi, notamment concernant tout évènement indésirable grave. Aucune modification ne peut être apportée au protocole sans évaluation et approbation par le comité.

La recherche médicale impliquant des êtres humains doit être conduite uniquement par des personnes scientifiquement qualifiées et expérimentées. La recherche impliquant des patients ou des volontaires en bonne santé nécessite la supervision d'un médecin ou d'un autre professionnel de santé qualifié et compétent. La responsabilité de protéger les personnes impliquées dans la recherche doit toujours incomber à un médecin ou à un autre professionnel de santé et jamais aux personnes impliquées dans la recherche même si celles-ci ont donné leur consentement.

La recherche médicale impliquant une population ou une communauté défavorisée ou vulnérable se justifie uniquement si la recherche répond aux besoins et priorités sanitaires de cette population ou communauté et si, selon toute vraisemblance, les résultats de la recherche seront bénéfiques à cette population ou communauté.

Toute recherche médicale impliquant des êtres humains doit préalablement faire l'objet d'une évaluation soigneuse des risques et des inconvénients prévisibles pour les personnes et les

communautés impliquées dans la recherche, par rapport aux bénéfices prévisibles pour elles et les autres personnes ou communautés affectées par la pathologie étudiée.

Tout essai clinique doit être enregistré dans une banque de données accessible au public avant que ne soit recruté la première personne impliquée dans la recherche.

Les médecins ne sont pas autorisés à participer à une recherche impliquant des êtres humains sans avoir la certitude que les risques inhérents ont été correctement évalués et pourront être gérés de manière satisfaisante. Les médecins doivent cesser immédiatement une étude dès que les risques s'avèrent dépasser les bénéfices potentiels ou dès l'instant où des résultats positifs et bénéfiques ont été démontrés.

Une recherche médicale impliquant des êtres humains ne peut être conduite que si l'importance de l'objectif dépasse les risques et inconvénients inhérents pour les personnes impliquées dans la recherche.

La participation de personnes capables à une recherche médicale doit être un acte volontaire. Bien qu'il puisse être opportun de consulter les membres de la famille ou les responsables de la communauté, aucune personne capable ne peut être impliquée dans une étude sans qu'elle ait donné librement son consentement.

Toutes les précautions doivent être prises pour protéger la vie privée et la confidentialité des informations personnelles concernant les personnes impliquées dans la recherche, et pour minimiser l'impact de l'étude sur leur intégrité physique, mentale et sociale.

Dans la recherche médicale impliquant des personnes capables, toute personne pouvant potentiellement être impliquée dans la recherche doit être correctement informé des objectifs, des méthodes, des sources de financement, de tout éventuel conflit d'intérêts, des affiliations institutionnelles du chercheur, des bénéfices escomptés et des risques potentiels de l'étude, des désagréments qu'elle peut engendrer et de tout autre aspect pertinent de l'étude. La personne pouvant potentiellement être impliquée dans la recherche doit être informé de son droit de refuser de participer à l'étude ou de s'en retirer à tout moment sans mesure de rétorsion. Une attention particulière devrait être accordée aux besoins d'informations spécifiques de chaque personne pouvant potentiellement être impliquée dans la recherche ainsi qu'aux méthodes adoptées pour fournir les informations. Lorsque le médecin ou une autre personne qualifiée en la matière a la certitude que la personne concernée a compris les informations, il doit alors solliciter son consentement libre et éclairé, de préférence par écrit. Si le consentement ne peut pas être donné par écrit, le consentement non écrit doit être formellement documenté en présence d'un témoin.

Pour la recherche médicale utilisant des tissus ou des données d'origine humaine, les médecins doivent normalement solliciter le consentement pour le prélèvement, l'analyse, le stockage et/ou la réutilisation. Il peut se présenter des situations où il est impraticable, voire impossible d'obtenir le consentement ou que cela mettrait en péril la validité de la recherche. Dans de telles situations, la recherche peut être entreprise uniquement après évaluation et approbation d'un comité d'éthique de la recherche.

Lorsqu'il sollicite le consentement éclairé d'une personne pour sa participation à une recherche, le médecin devrait être particulièrement attentif lorsque cette dernière est dans une relation de dépendance avec lui ou pourrait donner son consentement sous la contrainte. Dans ce cas, le consentement éclairé devrait être sollicité par une personne qualifiée en la matière et complètement indépendante de cette relation.

Lorsque la recherche implique des personnes incapables, le médecin doit solliciter le consentement éclairé de leur représentant légal. Les personnes incapables ne doivent pas être inclues dans une étude qui n'a aucune chance de leur être bénéfique sauf si cette étude vise à améliorer la santé de la population qu'elles représentent, qu'elle ne peut pas être réalisée avec des personnes capables et qu'elle ne comporte que des risques et des inconvénients minimes.

Lorsqu'une personne considérée comme incapable est en mesure de donner son assentiment concernant sa participation à la recherche, le médecin doit solliciter cet assentiment en complément du consentement de son représentant légal. Le refus de la personne pouvant potentiellement être impliquée dans la recherche devrait être respecté.

La recherche impliquant des personnes physiquement ou mentalement incapables de donner leur consentement, par exemple des patients inconscients, peut être menée uniquement si l'état physique ou mental empêchant de donner un consentement éclairé est une caractéristique nécessaire de la population sur laquelle porte cette recherche. Dans de telles circonstances, le médecin devrait solliciter le consentement éclairé du représentant légal. En l'absence d'un représentant légal et si la recherche ne peut pas être retardée, l'étude peut être lancée sans le consentement éclairé. Dans ce cas, le protocole de recherche doit mentionner les raisons spécifiques d'impliquer des personnes dont l'état les rend incapables de donner leur consentement éclairé et l'étude doit être approuvée par un comité d'éthique de la recherche. Le consentement pour maintenir la personne concernée dans la recherche devrait, dès que possible, être obtenu de la personne elle-même ou de son représentant légal.

Les auteurs, rédacteurs et éditeurs ont tous des obligations éthiques concernant la publication des résultats de recherche. Les auteurs ont le devoir de mettre à la disposition du public les résultats de leurs recherches sur les êtres humains. Ils ont la responsabilité de fournir des rapports complets et précis. Ils devraient se conformer aux directives acceptées en matière d'éthique pour la rédaction de rapports. Les résultats aussi bien négatifs et non concluants que positifs devraient être publiés ou rendus publics par un autre moyen. La publication devrait mentionner les sources de financement, les affiliations institutionnelles et les conflits d'intérêts. Les rapports de recherche non-conformes aux principes de la présente Déclaration ne devraient pas être acceptés pour publication.

PRINCIPES ADDITIONNELS POUR LA RECHERCHE MEDICALE ASSOCIEE A DES SOINS MEDICAUX

Le médecin peut associer la recherche médicale à des soins médicaux uniquement dans la mesure où la recherche se justifie par sa valeur potentielle en matière de prévention, de diagnostic ou de traitement et si le médecin a de bonnes raisons de penser que la participation à l'étude ne portera pas atteinte à la santé des patients concernés.

Les bénéfices, les risques, les inconvénients, ainsi que l'efficacité d'une nouvelle intervention doivent être testés et comparés à ceux de la meilleure intervention courante avérée, sauf dans les circonstances suivantes :

L'utilisation de placebo, ou le fait de ne pas administrer de traitement, est acceptable lorsqu'il n'existe pas d'intervention courante avérée ; ou l'utilisation d'un placebo afin de déterminer l'efficacité ou la sécurité d'une intervention est nécessaire pour des raisons de méthodologie incontournables et scientifiquement fondées, et les patients recevant le placebo ou aucun traitement ne courent aucun risque de préjudices graves ou irréversibles. Le plus grand soin doit être apporté afin d'éviter tout abus de cette option.

A la fin de l'étude, les patients impliqués ont le droit d'être informés des conclusions de l'étude et de profiter de tout bénéfice en résultant, par exemple, d'un accès aux interventions identifiées comme bénéfiques dans le cadre de l'étude ou à d'autres soins ou bénéfices appropriés. Le médecin doit fournir des informations complètes au patient sur la nature des soins liés à la recherche. Le refus d'un patient de participer à une étude ou sa décision de s'en retirer ne doit jamais interférer avec la relation patient-médecin. Dans le cadre du traitement d'un patient, faute d'interventions avérées ou faute d'efficacité de ces interventions, le médecin, après avoir sollicité les conseils d'experts et avec le consentement éclairé du patient ou de son représentant légal, peut recourir à une intervention non avérée si, selon son appréciation professionnelle, elle offre une chance de sauver la vie, rétablir la santé ou alléger les souffrances du patient. Dans toute la mesure du possible, cette intervention

devrait faire l'objet d'une recherche pour en évaluer la sécurité et l'efficacité. Dans tous les cas, les nouvelles informations devraient être enregistrées et, le cas échéant, rendues publiques.

Annexe 9. Epicentre au Niger

Epicentre mène des activités de recherche depuis 1989 au Niger, dans la région de Maradi. En partenariat avec le Ministre de la Sante, les ONG et les sections de MSF, des partenaires locaux, les autorités locales et d'autres agences internationales, Epicentre développe des projets de recherche répondant aux besoins sanitaires de la population. Epicentre a une base de recherche à Maradi (incluant un département logistique, administratif, un laboratoire hébergé par le Centre Hospitalier Régional (CHR) de Maradi, un département de saisie et gestion des données), ainsi qu'une activité de coordination, de représentation et de collaboration avec les partenaires à Niamey. Epicentre peut, en reposant sur ces structures et son personnel permanents, mener des projets dans diverses régions du Niger, sur des thèmes aussi variés que les maladies infectieuses, la vaccination, la malnutrition et la santé mentale.
